# Fluorine‐Induced *Pseudo*‐Anomeric Effects in Methoxycyclohexanes through Electrostatic 1,3‐Diaxial Interactions

**DOI:** 10.1002/chem.202003058

**Published:** 2020-08-18

**Authors:** Bruno A. Piscelli, William Sanders, Cihang Yu, Nawaf Al Maharik, Thomas Lebl, Rodrigo A. Cormanich, David O'Hagan

**Affiliations:** ^1^ Chemistry Institute University of Campinas Monteiro Lobato Street Campinas, Sao Paulo 13083-862 Brazil; ^2^ School of Chemistry University of St Andrews North Haugh St Andrews KY16 9ST UK; ^3^ Department of Chemistry Faculty of Science An-Najah National University Nabulus West Bank, Palestine P.O. Box 7 Palestine

**Keywords:** anomeric effects, computational chemistry, conformational analysis, fluorination, medicinal chemistry

## Abstract

We report counter‐intuitive axial preferences in non‐stereochemically biased, selectively fluorinated methoxycyclohexanes. These pseudo‐anomeric effects are apparent when electronegative CF_2_ groups are placed at the C‐2, C‐4 and C‐6 positions of the cyclohexane ring to render the C‐3/5 axial hydrogen atoms electropositive. The electrostatic interaction between these axial hydrogen atoms and the ‐OMe oxygen is stabilising. The effect is explored using high‐level ab initio and DFT calculations in the framework of NBO, QTAIM and NCI analysis across a range of derivatives, and experimentally (^19^F{^1^H}‐NMR at −80 °C) for some illustrative examples. The effect is significant in energy terms for a weak interaction, and illustrates a new stereoelectronic aspect attributed to selective fluorine substitution in organic chemistry.

## Graphical abstract

**Fluorine chemistry**: Selective fluorination in methoxycyclohexanes induces an electrostatic 1,3‐diaxial interaction favouring a counter‐intuitive axial preference.

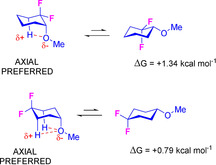

It is well‐known that 2‐methoxypyran **1** displays an ‘anomeric’ or ‘Edward–Lemieux’ effect^1^, ^2^ where ring interconversion favours the axial (**1**_ax_) over equatorial (**1**_eq_) conformer (gas phase Δ*G*=≈0.8–0.9 kcal mol^−1^ experimentally), an effect that diminishes in increasingly polar solvents to **1**_ax_=**1**_eq_ parity in water.^3^ The electrostatic versus stereoelectronic origin of the anomeric effect has received considerable attention (Figure 1).^4^, ^5^

**Figure 1:**
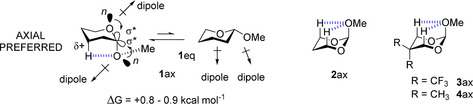
Superimposition of some origins of the anomeric effect in 2‐methoxypyran **1** and 1,3‐diaxial CH⋅⋅⋅*n* interactions (dashed lines) in dioxolanes **2** and **3**.

The first interpretations for the anomeric effect argued destabilising dipole–dipole repulsion between the oxygen lone pairs in the equatorial anomer.^1^, ^6^ For example anomer **1**_ax_ is less polar than **1**_eq_ due to an antiparallel alignment of opposing dipoles. This is in line with diminishing anomeric stabilisation in solvents of increasing polarity. A ‘double bond/no bond’ analysis emerged a decade later which was in line with bond lengths emerging from crystallography and theory.^6^ This argued, for example, that in pyran **1_ax_**, hyperconjugative interactions between a lone pair of the ring oxygen and the σ*_C−O_ antibonding orbital of the methoxyl group (*endo* anomeric effect) stabilises **1**_ax_. Subsequently a reciprocal *exo*‐anomeric was argued recognising hyperconjugation between the methoxyl oxygen and the σ*_C−O_ orbital of the ring oxygen.^7^, ^8^, ^9^ This too is consistent with a diminished anomeric effect in more polar solvents. More recent assessments indicate that there are other important forces operating too. Takahashi *et al*.,^10^, ^11^ have recognised the importance of coloumbic/electrostatic 1,3‐diaxial CH⋅⋅⋅*n*(lone pair) interactions stabilising **1**_ax_ and also the axial conformers of 1,3‐dioxolanes such as **2**_ax_, and have argued that such effects contribute to anomeric stabilisation in carbohydrates too. Wiberg *et al*.,^12^ have long contributed to the discussion and summed up the complexity of the various originating factors in a paper entitled ‘*The anomeric effect: It′s complicated’*. In that paper, evidence is presented to support a significant role for 1,3‐diaxial CH⋅⋅⋅*n* stabilisation, for example by exemplifying a significantly increased anomeric preference for 1,3‐dioxolanes **3** over **4**, due to the remote electronegative trifluoromethyl groups of **3** which increase polarisation of the axial hydrogens. Thus, the more recent discussion has tended to raise the profile of Coulombic forces and de‐emphasise that of the hyperconjugative rationale when exploring the origin of the anomeric effect. In this paper we show how the difluoromethylene group (CF_2_) can act as a surrogate *endo*‐O or can be placed as a substituent to promote axial stability in *cyclohexane* systems, by fully exploiting electrostatic 1,3‐diaxial CH⋅⋅⋅*n*_O_ interactions.

The high electronegativity of fluorine has resulted in a rich literature, particularly in medicinal and bioorganic chemistry, using fluorine to tune electronic profiles.^13^ In that context there is a tendency to try to establish surrogate motifs e.g. ‐CF_2_H for hydroxyl (OH)^14^ or vinyl fluoride for amide,13b although all such replacements have limitations. The direct replacement of difluoromethylene (‐CF_2_‐) for oxygen (‐*O*‐) has had some currency. The flagship arena for this replacement has been the difluoromethylene phosphonate (RCF_2_P(O)OH_2_) motif as a hydrolytically stable phosphate (ROP(O)OH_2_) analogue, a replacement that has been successfully explored in a range of circumstances.^15^ Also there is an active discussion in medicinal chemistry on using fluorine to influence molecular conformation of bioactives and particularly to access molecular conformers of varying Log Ps for dynamic transport to navigate membranes and respond to different intracellular environments as a drug journeys in vivo.^16^, ^17^

In the context of such a replacement it was previously reported that an *exo*‐anomeric effect was partially (≈50 %) restored in difluoromethylcyclohexane **6** relative to pyran **5** (Figure 2).^18^ The pendant OMe group prefers a *gauche* rather than an antiperiplanar conformation in each case indicating that the electronegativity of the fluorines re‐established the influence of the ether oxygen. This was not observed for the corresponding CH_2_ (cyclohexane) analogue. NBO analysis of **6** indicated lone pair donation from the exocyclic oxygen into the σ*_C−C(F2)_ antibonding orbital, and a weaker back donation of a lone pair of the axial fluorine into the exocyclic σ*_C−O_ orbital. The study demonstrated too that the solution structure of the maltose analogue **7** was also influenced by an *exo*‐anomeric preference, an effect that was lost when the CF_2_ group was replaced by CH_2_. Understanding fluorine effects is a subject of wide interest, and given this isolated study on regaining the *exo*‐anomeric effect, it seemed appropriate to explore the *endo*‐anomeric effect itself with an *endo* CF_2_. This required a simpler molecule, one where ring interconversion is not biased by the stereochemistry associated with the additional hydroxyl functionality found with the equatorial substituents in **5** and **6**. Therefore in this study a focus was placed on analogues of 2‐methoxypyran **1** where the oxygen atoms are replaced sequentially by difluoromethylene for **8** and **9** and then a double replacement in **10**. Indeed an axial preference for **8** has already been reported.^19^ Our study extended to cyclohexane **11**, where the fluorines are located at C4 remote from the methoxyl substituent.

**Figure 2:**
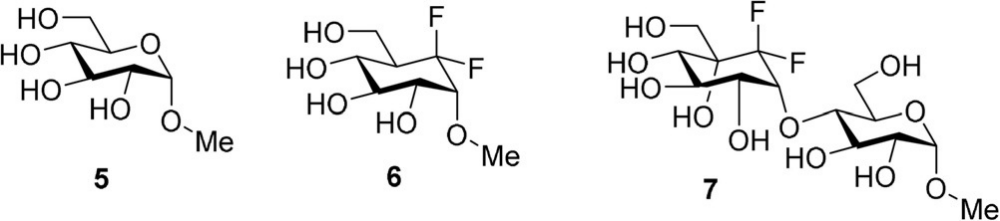
An *exo*‐anomeric effect has been argued for difluoromethylene cyclohexanes **6** and **7**.

Theory analysis was carried out in the gas phase and the resultant free energy differences (Δ*G* = *eq*−*ax*) for anomers of **8**–**11** are summarised in Figure 3. It emerged that **8** (Δ*G* = +1.34 kcal mol^−1^) and unexpectedly **11** (Δ*G* = +0.79 kcal mol^−1^) have an axial preference. On the other hand, compounds **9** and **10** have an equatorial preference.

**Figure 3:**
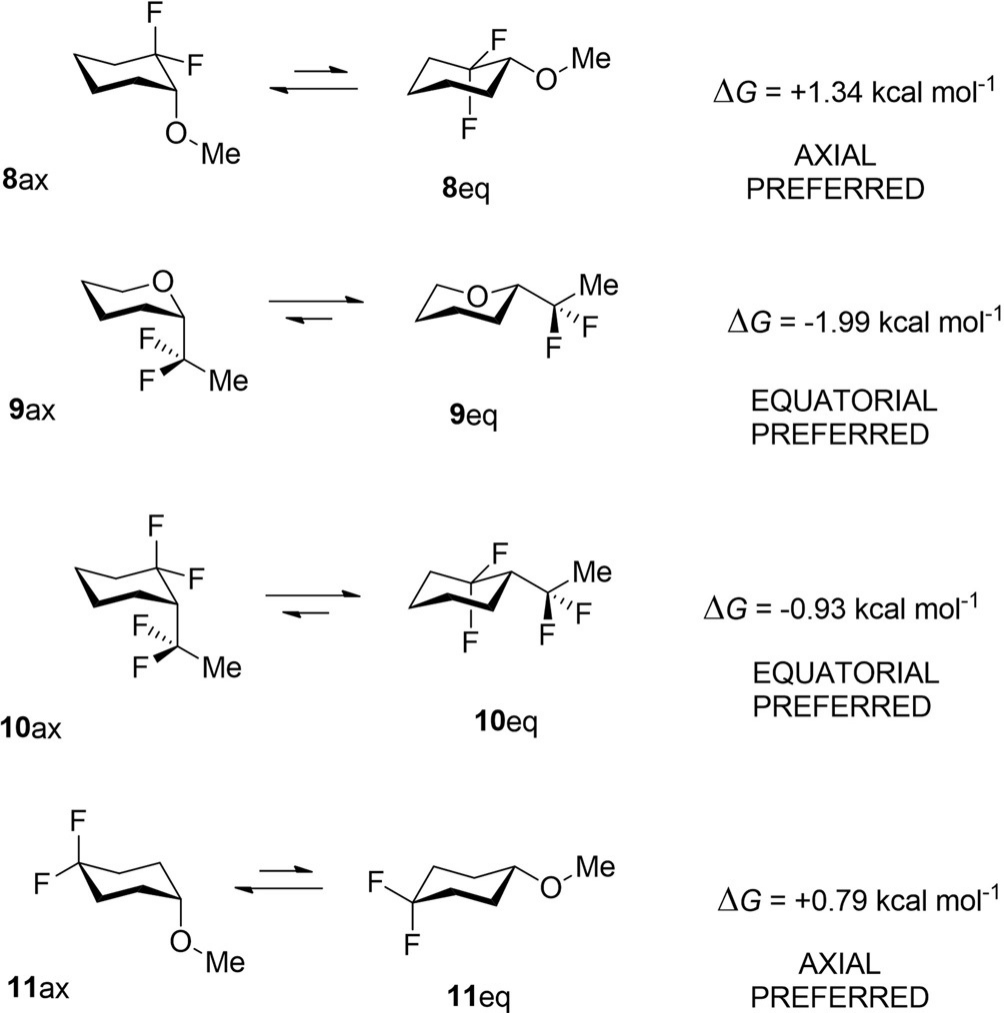
Calculated M06‐2X/aug‐cc‐pVTZ, free energy differences (Δ*G*=*eq*−*ax*) for **8**–**11** in the gas phase.

In order to support these studies experimentally, samples of compounds **8**, **10** and **11** were prepared by synthesis (see Figure S1), and their solution conformations explored by ^1^H and ^19^F{^1^H} NMR (Figure 4). In the case of **10** (see the Supporting Information) the equatorial conformer dominated^20^ as predicted by theory. For **8** and **11** at room temperature, ring interconversion was too rapid to resolve the axial and equatorial conformers; however, when the samples were cooled to −80 °C, then the individual AB‐signals of the fluorines in each conformer, were resolved. Hexane was selected as the least polar NMR solvent available to most closely approximate the gas phase calculations, and samples were also run in more polar dichloromethane. For cyclohexane **8** only the axial anomer **8**_ax_ is observed in hexane. However, in DCM both anomers are apparent with a slight bias in favour of **8**_eq_. It is well‐known that more polar conformers will be better accommodated in a more polar solvent,^21^ and this is the case here where calculated molecular dipole moments in dichloromethane (*μ“*; Table S21 in the Supporting Information) indicate that **8**_eq_ (*μ*=3.38 D) is more polar than **8**_ax_ (*μ*=1.86 D). However, the result that **8**_ax_ is exclusively observed in hexane is consistent with an inherent axial bias as suggested by gas‐phase calculations (97 % population at the M06‐2X/aug‐cc‐pVTZ level; Table S21 in the Supporting Information). The anomer ratio in DCM is reproduced when the calculations are carried out in a solvent dielectric continuum. NMR experiments were carried out for **11** at low temperature (−80 °C) in both DCM and hexane. It proved difficult to assign the signals for the **11**_ax_ and **11**_eq_ conformers by standard NMR methods. The alpha C1 hydrogen signal was very broad and featureless in the ^1^H NMR spectrum at low temperature, and no useful coupling constant information could be extracted to secure assignments. The ^19^F{^1^H} NMR AB‐signals were readily resolved; however, the remote nature of the fluorine atoms to the anomeric centre did not offer any discriminating coupling constants to differentiate conformers. In the end, assignments were made by theory, calculating the relative ^19^F NMR chemical shifts of the anomers at the mPW1PW91/6‐31G(d) level,^22^ and this data is summarised in Figure S5 in the SI. Both of the fluorine signals for the AB‐system for **11**_ax_ have chemical shifts within the signals of the AB system for **11** equiv. The theory outcome matches experiment very closely and aided the assignment of the chemical shifts of the anomers of both compounds **8** and **11**. From this assignment it is clear that the axial conformer dominates in hexane in a ratio of **11**_ax_/**11**_eq_≈10:1, which is in excellent agreement with the calculated Gibbs free energy of 1.34 kcal mol^−1^ in the gas phase (ax:eq=88.7 %:11.3 %; Table S21). This ratio is reduced to **11**_ax_:**11**_eq_≈3:2 in dichloromethane, which is again in excellent agreement with the calculated Gibbs free energies of 0.27 kcal mol^−1^ (ax:eq=66.9 %/33.1 %; Table S21). Interestingly in this case it is actually the more polar conformer **11**_ax_, (*μ*=3.14) which dominates in hexane and the less polar **11**_eq_ (*μ*=2.85 D) which increases in the more polar solvent. This is counter‐intuitive, but consistent with the **11**_ax_ being stabilised by local *intramolecular* electrostatic interactions, which will be stronger in hexane and weakened by increasing the polarity of the solvent.

**Figure 4:**
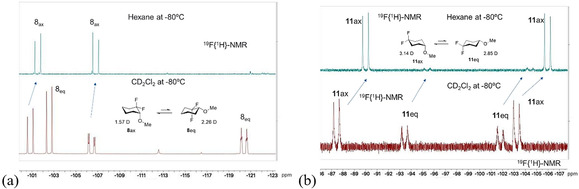
^19^F{^1^H} NMR spectra of **8** and **11** recorded in hexane or DCM at −80 °C. In each case the axial conformer **8**_ax_ or **11**_ax_ dominates in hexane. The more polar equatorial conformers emerge in the more polar solvent (DCM).

The axial preference for **11** cannot be accounted for by *n*_O_→σ*_C−C(F2)_ hyperconjugation due to the remoteness of the fluorines. Also dipole/dipole relaxation is inconsistent with the preference of **11**_ax_ over **11**_eq_, as **11**_ax_ is the more polar of the two anomers. In order to explore further and assess a role for 1,3‐diaxial C−O_ax_^*δ*−^⋅⋅⋅H_ax_^*δ*+^−C interactions, theory studies on a wider range of methoxycyclohexane combinations were carried out. These explored for example, placing CF_2_ groups at C‐2 and C‐4 as in **12** and C‐2, C‐4 and C6 as in **13**. The outcomes are presented in full in Table S21 (see Supporting Information) and selected examples are highlighted in Figure 5. In cases such as **12** and **13** the location of the CF_2_ groups significantly increases the axial preference and particularly so for cyclohexane **13**, where the predicted magnitude of the axial preference (Δ*G*=+ 3.32 kcal mol^−1^) is striking. The arrangement of three electronegative CF_2_ groups in **13** maximises the electropositive nature of the C‐3 axial hydrogens, increasing intramolecular electrostatic stabilisation. When CF_2_ groups are placed at C‐3, and C‐3 & C‐5 as in **14** and **15** respectively, then these systems revert to a strong equatorial preference due to electrostatic repulsions between 1,3‐diaxial O and F atoms which significantly destabilise axial conformers **14**_ax_ and **15**_ax_.

**Figure 5:**
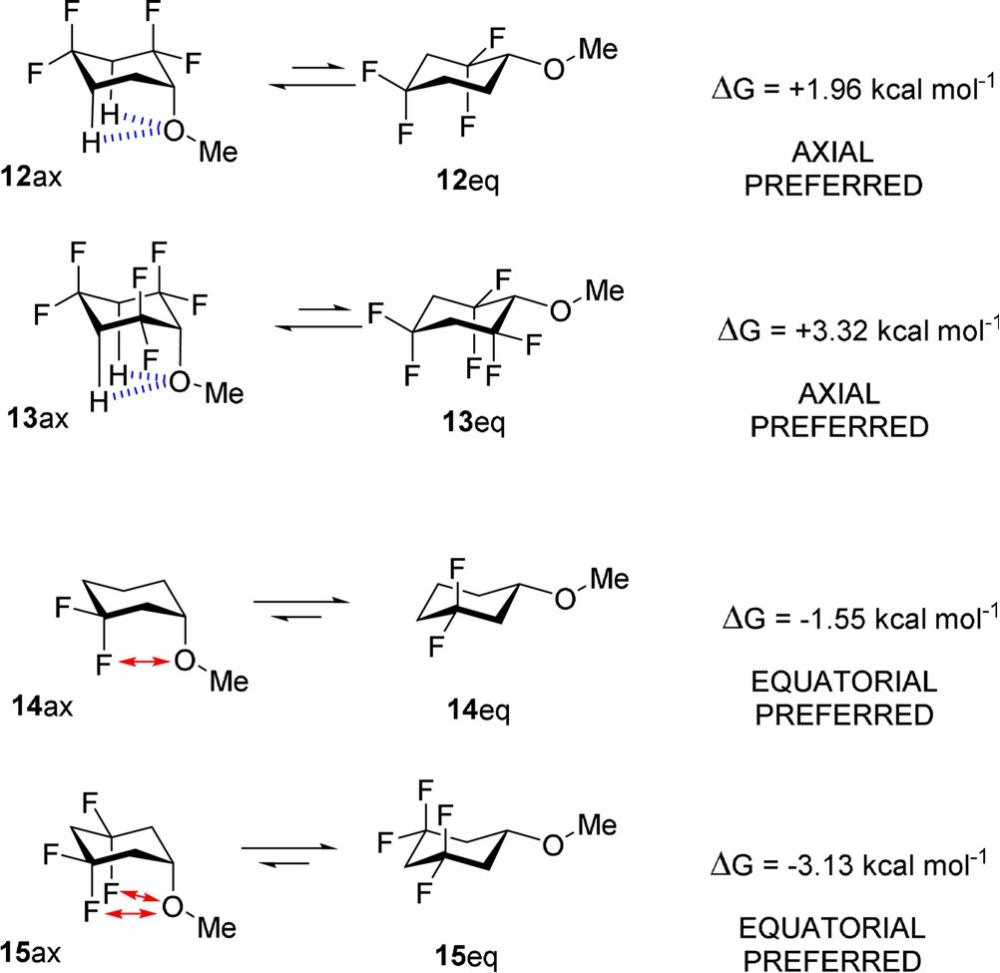
Calculated axial/equatorial energy preferences for **12**–**15** in the gas phase, and pictorial representation for electrostatic attraction (dashed lines) and repulsion (↔ ) in the axial conformers calculated at the M06‐2X/aug‐cc‐pVTZ level.

That electrostatic interactions are the most important driving force for the preference for the axial geometry was evidenced by deconstructing the total energy Δ*E*(T) of each conformer into its Lewis Δ*E*(L) and non‐Lewis Δ*E*(NL) contributions by NBO analysis^23^ and the results are summarized in the Table S1 (see in Supporting Information). The Δ*E*(L) contribution represents the hypothetical energy of the conformer without hyperconjugative stabilization, and Δ*E*(NL) the energy of stabilization by hyperconjugation. The Δ*E*(L) energy, which represents classical steric/electrostatic interactions, showed a higher weight than Δ*E*(NL) for the preference of the axial geometry. Thus, the total steric energy of each conformer was obtained by Natural Steric Analysis Δ*E*(NSA)^24^ and the total electrostatic energy by Natural Coulomb Energy analysis Δ*E*(NCE),^25^ which uses the classical Coulomb equation (*E*_NCE_=$\Sigma_{A,B}$ $Q_{A}Q_{B}$ /$R_{AB}$ ) and atomic charges derived from the Natural Population Analysis (NPA).

Accordingly, the Δ*E*(NCE) energy showed a higher contribution than Δ*E*(NSA) for axial stabilisation, mainly for molecules that had the ability to form strong C‐O_ax_^*δ*−^⋅⋅⋅H_ax_^*δ*+^‐C interactions, for example, **13**_ax_ has a total 28.3 kcal mol^−1^ higher electrostatic stabilization than **13**_eq_. The total electrostatic stabilization was further deconstructed to atom‐atom electrostatic interactions and those that make the axial geometry more stable are shown to be mainly H_ax_^*δ*+^−O^*δ*−^ attractive interactions (see Tables S2–S19 in the Supporting Information for full details). Such interactions were further studied using the quantum theory of atoms in molecules (QTAIM)^26^ and noncovalent interaction (NCI)^27^ methods (Table S20 and Figures S1–S3 in the Supporting Information).

Although QTAIM does not show a bond critical point for these 1,3‐diaxial interactions (Figure S1 in Supporting Information), all of the atomic properties used by QTAIM to characterize a conventional hydrogen bond (the so called Popelier criteria^26^) such as the atomic charge *q*(A), atomic energy *E*(A), first intramolecular atomic dipole moment *μ*_1_(A) and the atomic volume *V(*A) are fulfilled to indicate non‐classical C−O_ax_^*δ*−^⋅⋅⋅H_ax_^*δ*+^−C stabilising long range interactions (Table S20 in Supporting Information). The NCI method shows isosurfaces that indicate the formation of stabilising C−O_ax_^*δ*−^⋅⋅⋅H_ax_^*δ*+^−C interactions (Figures S2 and S3 in Supporting Information).

Of note are the calculated NPA charges and electrostatic interactions for representative molecules **8**_ax_–**15**_ax_ as illustrated in Figure 6. For **8**_ax_, the local electrostatic C−O_ax_^*δ*−^⋅⋅⋅H_ax_^*δ*+^‐C stabilising interaction is −15.5 kcal mol^−1^ (axial preference 0.49 kcal mol^−1^) and for **13**_ax_ this increases to −18.1 kcal mol^−1^, the system studied here with the highest axial preference (3.32 kcal mol^−1^). Similar trends are found for **11**_ax_ and **12**_ax_. These electrostatic interactions become stronger with the number of electronegative CF_2_ groups vicinal to the axial hydrogen atoms.

**Figure 6:**
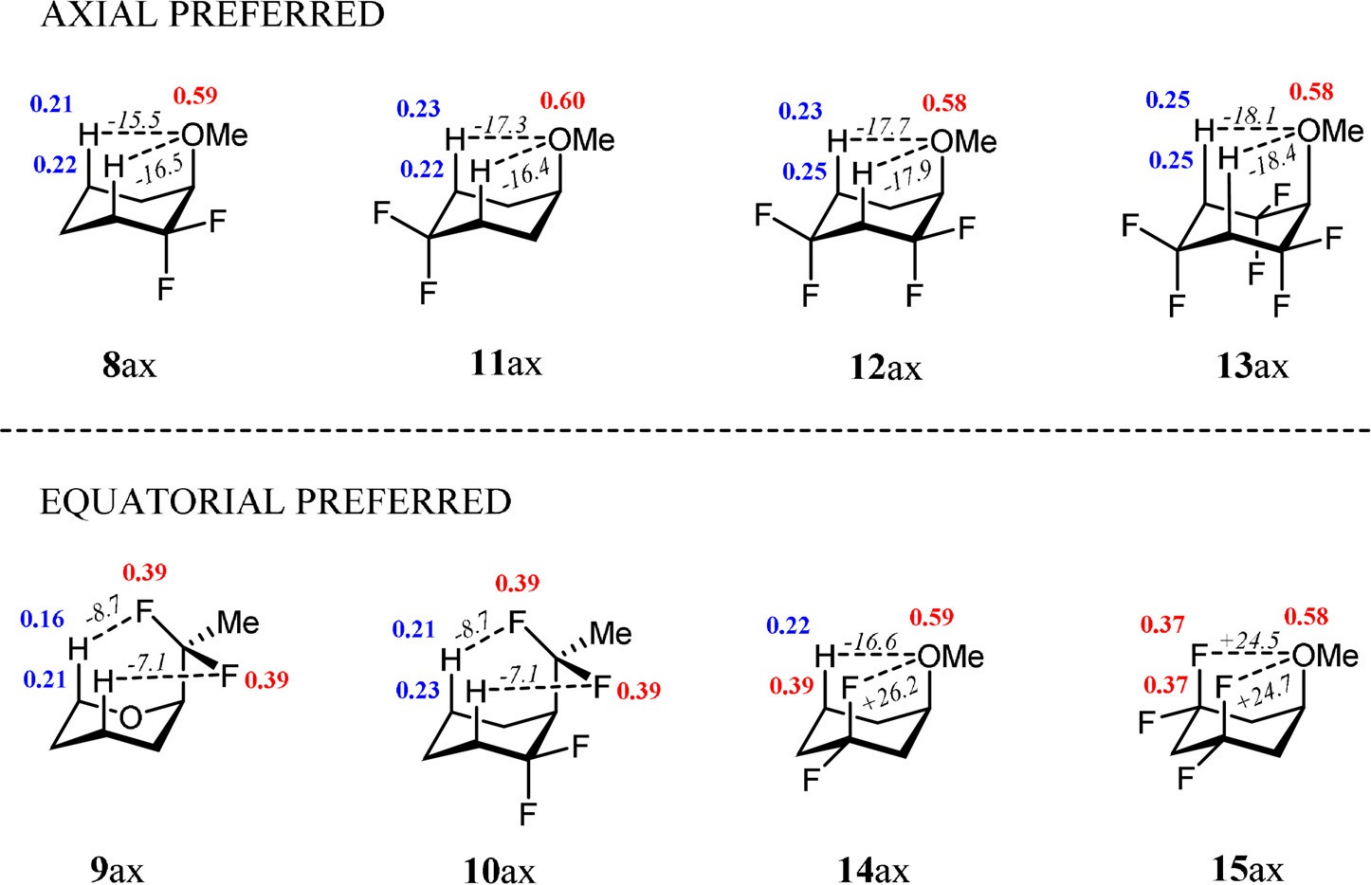
Calculated NPA atomic charges in atomic units (blue=positive; red=negative) at the M06‐2X/aug‐cc‐pVTZ level and with electrostatic interaction energies in kcal mol^−1^ (in italics, negative represent stabilising and positive represent destabilising) for **8**_ax_–**15**_ax_.

They also weaken with increasing polarity as evinced by calculations simulating solvents of increasing dielectric constants such as cyclohexane, chloroform, dichloromethane and acetone (Table S21 in Supporting Information). Consistent with this analysis, the axial preference decreases with increasing media polarity, even though e.g., **11**_ax_ is more polar than **11**_eq_.

On the other hand, **9**_ax_, **10**_ax_, **14**_ax_ and **15**_ax_ have either smaller local electrostatic stabilising interactions between the O^*δ*‐^_ax_ or F^*δ*‐^_ax_ and H^*δ*+^_ax_ atoms, such as −7.1 to −8.7 kcal mol^−1^ in **9**_ax_ and −6.9 to −11.4 kcal mol^−1^ in **10**_ax_ and, consequently, a preference for an equatorial geometry (by 2.55 and 1.57 kcal mol^−1^, respectively) or for **14**_ax_ and **15**_ax_ where they have strong C−F_ax_^*δ*−^⋅⋅⋅O_ax_^*δ*−^ destabilising electrostatic interactions (Figure 6).

As previously discussed, carbohydrate analogues **6** and **7** display an *exo*‐anomeric effect which was supported by Natural Bond Orbital (NBO) analyses and NMR spectroscopy. A similar NBO analysis was explored for **8**_ax_ and **11**_ax_. Rotational energy profiles (Figure 7 a,c) around the MeO−CH bond for the axial conformer **8**_ax_ indicates a narrow *gauche* preference (<CF_2_‐C‐*O*‐Me≈45°) for **8**_ax_. The outcome for **8**_ax_ mirrors that for carbohydrate analogues **6** and **7**,^18^ arising from lone pair donation from the exocyclic oxygen into the σ*_C−C(F2)_ antibonding orbital. This hyperconjugative interaction for **8**_ax_ is illustrated in the orbital image in Figure 7 b. The absence of such an effect in **11**_ax_ is clear and consistent with the remote location of the fluorine atoms.

**Figure 7:**
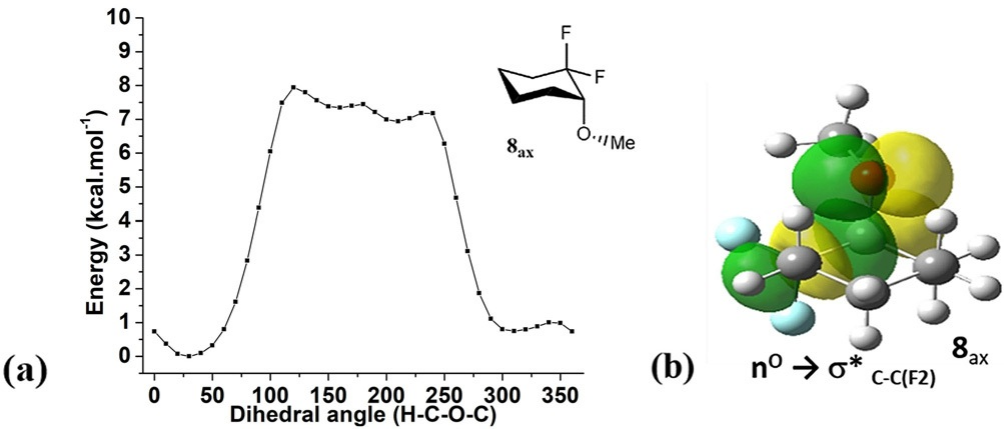
(a) Rotational energy profiles calculated at the M06‐2X/aug‐cc‐pVTZ for **8**_ax_ reporting relative energies as a result of rotation around the MeO−CH bond shows a narrow *gauche* preference (≈45°). (b) The orbital image illustrates a hyperconjugative interaction supporting an *exo*‐anomeric conformation in **8**_ax_, stabilised by lone pair donation from the exocyclic OMe oxygen into the σ*_C−C(F2)_ antibonding orbital.

In conclusion we have been able to demonstrate pseudo‐anomeric effects by placing difluoromethylene groups vicinal to axial hydrogen atoms at C‐3 in methyoxycyclohexanes and in cases, such as **8**, **12** and **13**, the axial preferences are similar or up to three times that of prototype **1**. These observations add to the discussion on factors that contribute to the anomeric effect itself and they clearly support a role for intramolecular 1,3‐diaxial attraction between polarised C−H(_ax_)^*δ*+^ hydrogen atoms and the anomeric oxygen ^*δ*−^OR. NBO does offer some support for *exo*‐hyperconjugative interactions in **8**_ax_. Given that **11**_ax_, which cannot accommodate such hyperconjugation has more than half the free energy (≈0.8 kcal mol^−1^) preference for the axial conformer than **8**_ax_ (1.34 kcal mol^−1^), then it is clear that although hyperconjugation plays a role, electrostatic forces dominate.

The observations here illustrate another aspect of the stereoelectronic influence of fluorine in small organic molecules and offers a design feature for influencing molecular conformation in organic materials or bioactives, and they contribute to the ongoing discussion on the origins of the anomeric effect.

## Conflict of interest

The authors declare no conflict of interest.

## Supporting information

As a service to our authors and readers, this journal provides supporting information supplied by the authors. Such materials are peer reviewed and may be re‐organized for online delivery, but are not copy‐edited or typeset. Technical support issues arising from supporting information (other than missing files) should be addressed to the authors.

Supplementary
