# Supplementary material for: Fluorine‐Induced Pseudo‐Anomeric Effects in Methoxycyclohexanes through Electrostatic 1,3‐Diaxial Interactions
Source: Chemistry. 2020 Aug 18;26(52):11989–94. doi: 10.1002/chem.202003058 (PMC7540582; doi:10.1002/chem.202003058)
Supplement: Supplementary file 1 — Supplementary [file CHEM-26-11989-s001.pdf]

Chemistry–A European Journal

Supporting Information

Fluorine-Induced *Pseudo*-Anomeric Effects in Methoxycyclohexanes through Electrostatic 1,3-Diaxial Interactions

Bruno A. Piscelli,[a] William Sanders,[b] Cihang Yu,[b] Nawaf Al Maharik,[b, c] Thomas Lebl,[b] Rodrigo A. Cormanich,*[a] and David O'Hagan*[b]

Table of Contents

1 Experimental Procedures **4**

Experimental Details 4

Protocol for synthesis of cyclohexane 8 4

^{1}H NMR cyclohexane 8 at room temperature. 5

^{19}F{^{1}H}-NMR (CD_2Cl_2) spectrum of cyclohexane 8 at -78ºC 6

^{19}F{^{1}H}-NMR variable temperature spectra of cyclohexane 8 in CDCl3 7

^{1}H-NMR of 10 at Room temperature in $CDCl_3$. 9

^{19}F{^{1}H}-NMR ($CDCl_3$) of 10 at room temperature (only one anomer apparent) 10

Protocol for synthesis of cyclohexane 11 11

^{1}H-NMR of 11 at -80oC in CD_2Cl_2 12

^{19}F{1H}-NMR (CD_2Cl_2 -80ºC) of 11 13

Computational details 14

2 Results and Discussion **15**

Table S1: Gas phase calculated total relative energy (ΔE), total relative enthalpy energy (ΔH) and total relative Gibbs free energy (ΔG) obtained at M06-2X/aug-cc-pVTZ theoretical level for compounds 1-15, in kcal mol^{-1}. Negative energy values represent axial preference, and the positive ones equatorial preference. 15

Table S2: NBO analysis relative energies (in kcal mol^{-1}) obtained at the M06-2X/aug-cc-pVTZ for compounds 1-15 . Negative values represent a preference for the axial conformer and positive ones for the equatorial conformer. 16

Table S3. Atom-atom electrostatic interactions (kcal mol^{-1}) obtained at M06-2X/aug-cc-pVTZ level using NPA charges for 1_{ax}. 17

Table S4. Atom-atom electrostatic interactions (kcal mol^{-1}) obtained at M06-2X/aug-cc-pVTZ level using NPA charges for 1_{eq}. 19

Table S5. Atom-atom electrostatic interactions (kcal mol^{-1}) obtained at M06-2X/aug-cc-pVTZ level using NPA charges for 8_{ax}. 21

Table S6. Atom-atom electrostatic interactions (kcal mol^{-1}) obtained at M06-2X/aug-cc-pVTZ level using NPA charges for 8_{eq}. 22

Table S7. Atom-atom electrostatic interactions (kcal mol^{-1}) obtained at M06-2X/aug-cc-pVTZ level using NPA charges for 9_{ax}. 24

Table S8. Atom-atom electrostatic interactions (kcal mol^{-1}) obtained at M06-2X/aug-cc-pVTZ level using NPA charges for 9_{eq}.. 26

Table S9. Atom-atom electrostatic interactions (kcal mol^{-1}) obtained at M06-2X/aug-cc-pVTZ level using NPA charges for 10_{ax}.. 27

Table S10. Atom-atom electrostatic interactions (kcal mol^{-1}) obtained at M06-2X/aug-cc-pVTZ level using NPA charges for 10_{eq}.. 29

Table S11. Atom-atom electrostatic interactions (kcal mol^{-1}) obtained at M06-2X/aug-cc-pVTZ level using NPA charges for 11_{ax}.. 30

Table S12. Atom-atom electrostatic interactions (kcal mol^{-1}) obtained at M06-2X/aug-cc-pVTZ level using NPA charges for 11_{eq}.. 32

Table S13. Atom-atom electrostatic interactions (kcal mol^{-1}) obtained at M06-2X/aug-cc-pVTZ level using NPA charges for 12_{ax}.. 33

Table S14. Atom-atom electrostatic interactions (kcal mol^{-1}) obtained at M06-2X/aug-cc-pVTZ level using NPA charges for 12_{eq}.. 35

Table S15. Atom-atom electrostatic interactions (kcal mol^{-1}) obtained at M06-2X/aug-cc-pVTZ level using NPA charges for 13_{ax}.. 36

Table S16. Atom-atom electrostatic interactions (kcal mol^{-1}) obtained at M06-2X/aug-cc-pVTZ level using NPA charges for 13_{eq}.. 38

Table S17. Atom-atom electrostatic interactions (kcal mol^{-1}) obtained at M06-2X/aug-cc-pVTZ level using NPA charges for 14_{ax}.. 39

Table S18. Atom-atom electrostatic interactions (kcal mol^{-1}) obtained at M06-2X/aug-cc-pVTZ level using NPA charges for 14_{eq}.. 41

Table S19. Atom-atom electrostatic interactions (kcal mol^{-1}) obtained at M06-2X/aug-cc-pVTZ level using NPA charges for 15_{ax}.. 42

Table S20. Atom-atom electrostatic interactions (kcal mol^{-1}) obtained at M06-2X/aug-cc-pVTZ level using NPA charges for 15_{eq}.. 44

Table S21. Popelier criterea parameters from QTAIM calculations run at the M06-2X/aug-cc-pVTZ wavefunction density for the molecules with axial hydrogens in positions 3 and/or 5 for compounds 1-15.. 45

Figure S1. QTAIM molecular graphs for molecules 1-15. bond critical points (green spheres), ring critical points (red spheres) and cage critical points (blue shperes)................................ 46

Figure S2. Isosurfaces from NCI for molecules 1-15, using reduced density gradient (RDG) = 0.5 and blue-green-red color scale ranging from $-0.02 < \mathrm{sign}(\lambda_2)\rho(\mathrm{r}) < +0.02$ au.................. 47

Figure S3: Reduced RDG versus $\mathrm{sign}(\lambda_2)\rho$ plots for compounds 1-15. 48

Table S22. Calculated total relative energy ΔE and relative Gibbs energy values in kcal mol^{-1} and ax/eq population percentages obtained at DLPNO-CCSD(T) and M06-2X/aug-cc-pVTZ[levels for compounds 1-15. Relative Gibbs free energies (kcal mol^{-1}) and population percentages are also shown in different solvents using the polarizable continuum model at the M06-2X/aug-cc-pVTZ level. Dipole moments (μ) calculated in each medium at the M06-2X/aug-cc-pVTZ

level are also given in Debyes. Negative energy values represent axial preference, and the positive ones equatorial preference. 49

Figure S4. Potential energy curves (kcal mol^{-1}) for the rotation of the H-C-O-C dihedral angle from 0-360º in steps of 10º for molecules 1-15 calculated at the M06-2X/aug-cc-pVTZ level. 52

Figure S5. Calculated (in italics) and experimental (in bold) ^{19}F-NMR chemical shits in ppm for the anomers 8_{ax} and 8_{eq} and 11_{ax} and $11_{eq.}$ The calculated values were obtained at the mPW1PW91/6-31G(d) level by the GIAO method and corrected using a scaling factor of 0.84. 53

Table S25. Cartesian coordinates of the optimized geometries for the axial and equatorial geometries of compounds 1-15 obtained at the M06-2X/aug-cc-pVTZ level in the gas phase. 56

3 References 59

1 Experimental Procedures

Experimental Details

Protocol for synthesis of cyclohexane 8

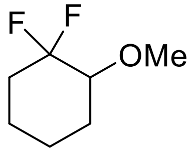

8

DAST (3 cm^3) was added dropwise to a solution of 2-methoxycyclohexanone (10 mmol) in DCM (30 cm^3) and the reaction stirred at room temperature for 5 min. Once the addition was complete HF/pyridine (2 cm^3) was added dropwise with stirring, over 5 min. The reaction vessel was then left to stir for 90 min monitoring progress by TLC. Once the reaction had completed the resulting solution was poured over aqueous sodium carbonate and then additional solid sodium carbonate was added until the pH of the mixture was basic (universal indicator paper). The mixture was extracted into DCM (x 3) and the combined organic layers were washed with 1M HCl (aq). The organic layer was dried over solid anhydrous $MgSO_4$ and the solvent removed under reduced pressure. The product was purified over silica gel eluting with petroleum ether and diethyl ether (95:5 respectively) to yield the cyclohexane **8** as a colourless liquid (946 mg, 63 %).

The product had traces of by-product 1-fluoro-2-methoxycyclohex-1-ene, which were not removed by chromatography. Therefore ozone was bubbled through a cooled (-78°C) solution of the mixture (100 mg) in DCM (10ml) until a persistent blue colour was observed. Dimethyl sulfide (2.3 cm^3) was added and the reaction left to stir for 2 h. The resulting solution was concentrated and purified over silica gel using petroleum ether and diethyl ether (95:5 respectively) as the eluent to give

cyclohexane **8** as a colourless liquid (69 mg, 69 %).^{1}H NMR (500 MHz $CDCl_3$,): 1.5-1.7 (m, 4H), 1.75 (m, 3H), 2.00 (m, 1H), 3.4 (m, 1H), 3.5 (s, 3H); $^{19}F\{^{1}H\}$-NMR(470 MHz $CDCl_3$, -78ºC); -100.84 (d, 244.4Hz, $1F_{OMe\ ax}$), -102.54 (d, 230.3Hz, $1F_{OMe\ eq}$), -106.44 (m.d, 244.4Hz, $1F_{OMe\ eq}$), -120.44 (d, 235.0Hz, $1F(_{OMe\ ax})$.

Also see VT spectra below.

^{1}H NMR cyclohexane 8 at room temperature.

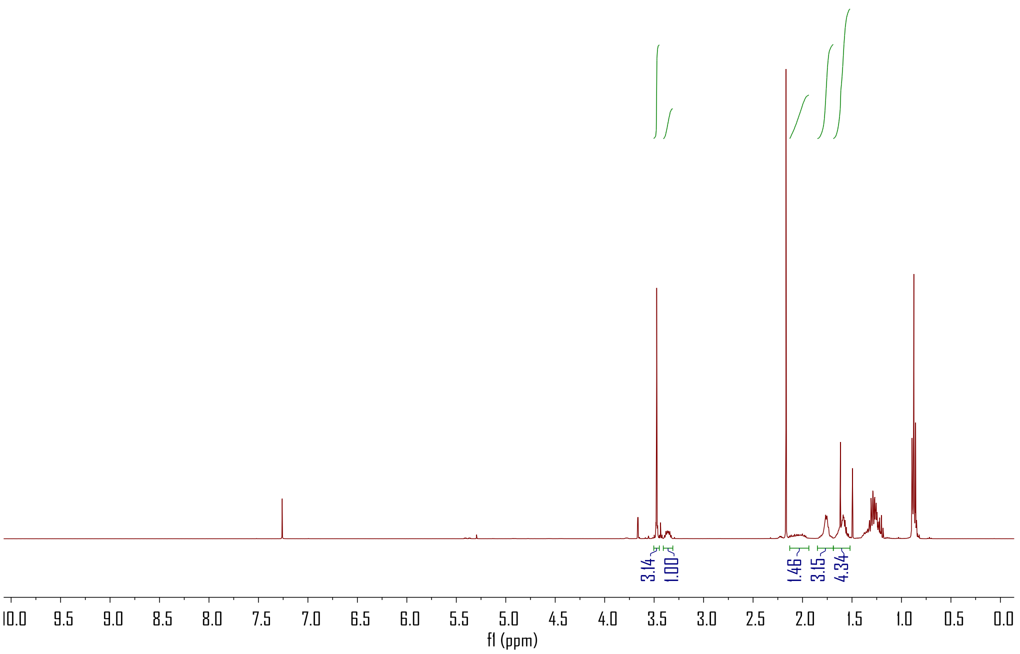

*singlet peak at 2.16 is acetone residue, peak at 0.8-1.3 are pentane residues

$^{19}F\{^1H\}$-NMR (CD_2Cl_2) spectrum of cyclohexane 8 at -78ºC

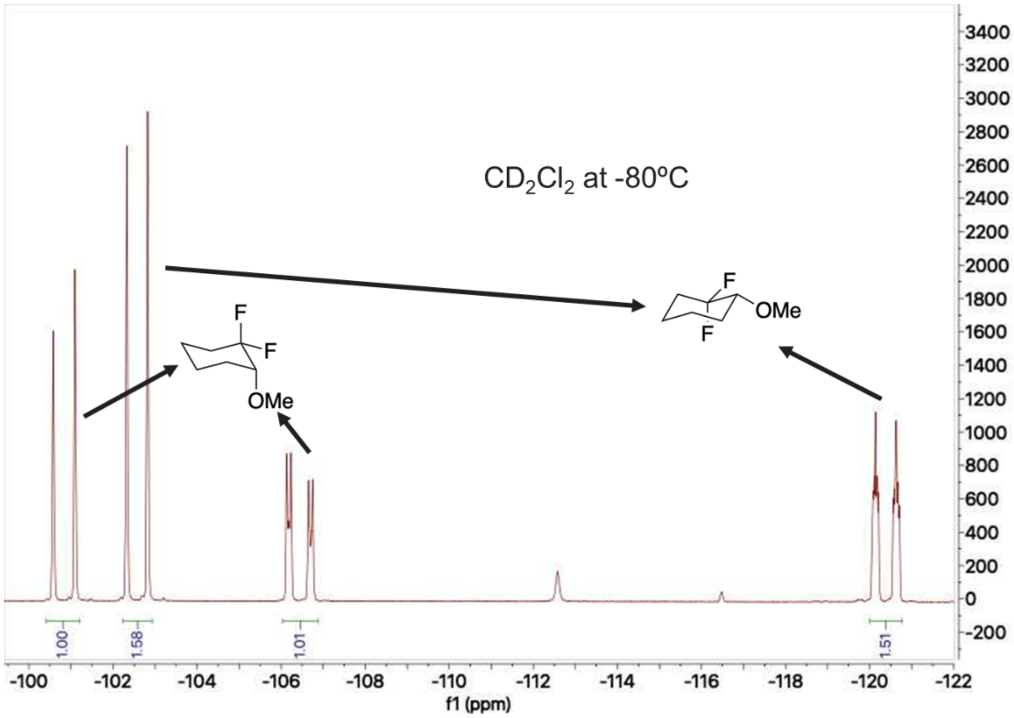

Assignments were made using Bruker-DAISY simulation software (see below).

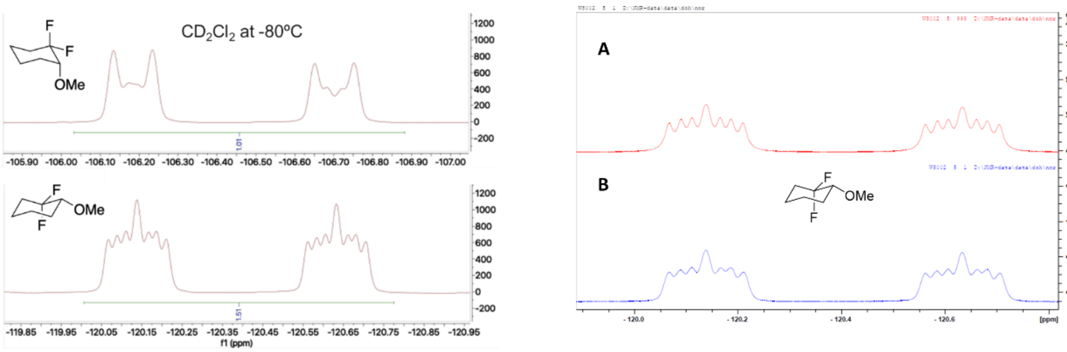

(Left) ^{19}F-NMR Peak patterns in experimental NMR of **8**. (Right) Assignment of **8eq** using Bruker DAISY software. Smulated signal pattern (**B**) matched experimental pattern (**A**)

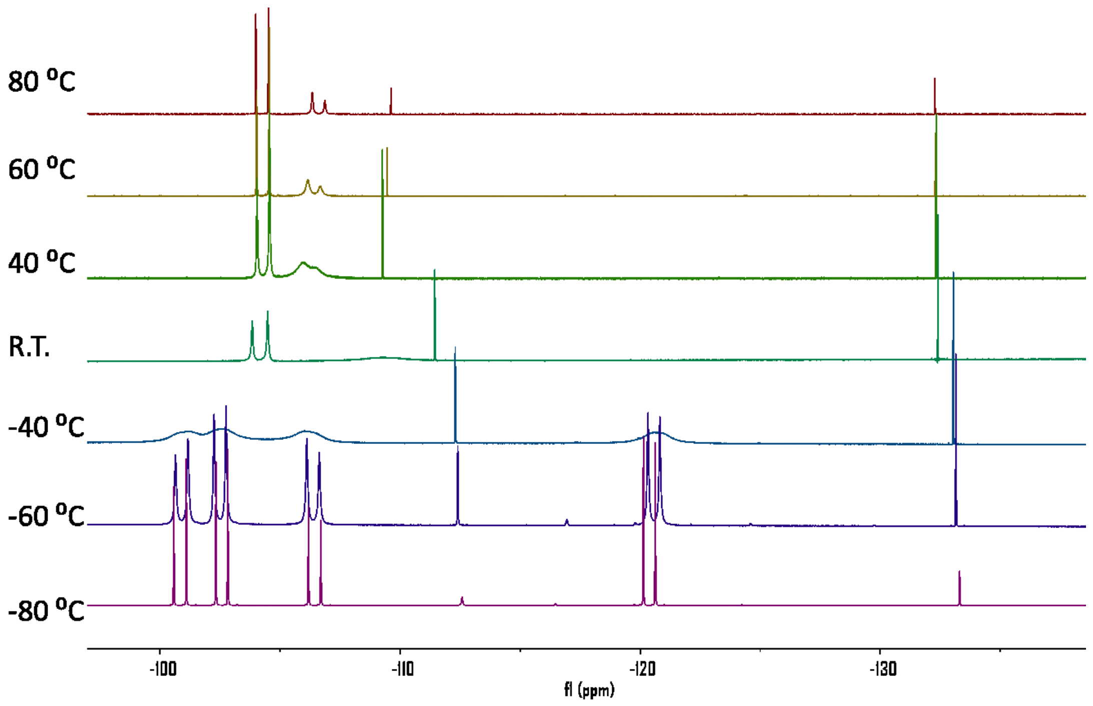

$^{19}F\{^1H\}$-NMR variable temperature spectra of cyclohexane 8 in CDCl3

Protocol for synthesis of cyclohexane **10**

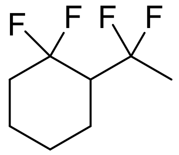

10

Xtalfluor E (3.4 g, 14.85 mmol) was placed in a Teflon flask under an Argon atmosphere. 2-Acetylcyclohexanone (1 g, 7.13 mmol), dichloromethane (3 mL) and $Et_3N.HF$ (1ml, 6.13 mmol) were subsequently added. After 24 hrs stirring at room temperature, DAST (4.7mL, 35.67 mmol) was added and the reaction was stirred at ambient for 72 hrs. The mixture was poured into ice-water and extracted with pentane (50 mL x 3), dried over $MgSO_4$, and most of pentane was

removed under reduced pressure (600 mm Hg pressure) at 30 °C. Purification of the orange residue over silica gel using pentane and then pentane:diethyl ether (98:2) gave a sample of 2-(1,1-difluoroethyl)cyclohexanone **10** (178 mg, 15%). ^{1}H NMR (500 MHz $CDCl_3$,): 2.02-2.31 (m, 3H), 1.74 (tt, *J* = 19.7, 1.7 Hz, 3H), 1.52-1.89 (m, 6H); δ ^{1}H{^{19}F} 2.24 (dd, *J* = 12.5, 4.1 Hz, 1H), 2.16 (dm, 13.8 Hz, 1H), 2.03-2.08 (m, 1H), 1.77-1.89 (m, 2H), 1.74 (s, 3H), 1.53-1.71 (m, 3H); ^{13}C NMR (125 MHz, $CDCl_3$): δ 122.9 (t, *J* = 242.0 Hz), 122.2 (tt, *J* = 243.3, 4.5 Hz), 50.2 (m, *J* = 18.2, 17.7 Hz), 35.24 (t, *J* = 24.0 Hz), 23.82, 23.63 (q, *J* = 5.6 Hz), 22.80 (tt, *J* = 27.8, 3.4 Hz), 22.53 (d, J = 9.2 Hz); **^{19}F{^{1}H} NMR** (282 MHz, $CDCl_3$) δ -84.75 (1F, ddd, *J* = 252.2, 16.9, 3.4 Hz), -90.41(1F, ddd, *J* = 252.2, 13.4, 3.4 Hz), -92.55 (1F, ddd, *J* = 238.6, 16.2, 3.8 Hz), -92.55 (1F, brd, *J* = 238.6 Hz); HRMS (ESI, +ve) *m/z* calculated for $C_8H_{12}F_4Na$ [M+Na$^+$] 207.07673, found 207.07580.

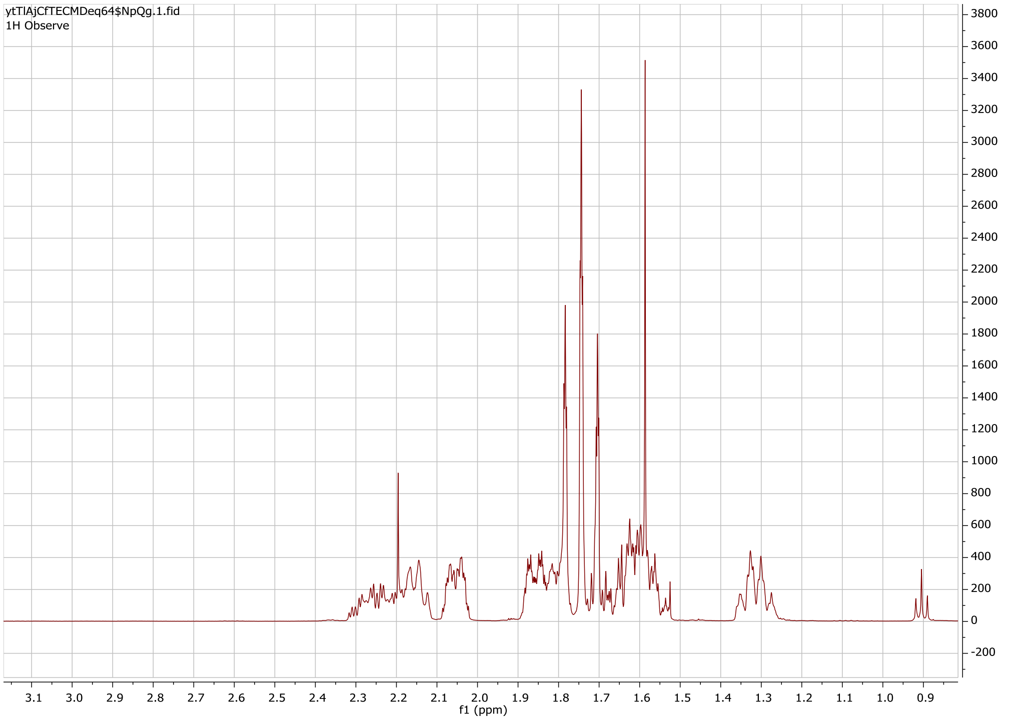

^{1}H-NMR of 10 at Room temperature in $CDCl_3$.

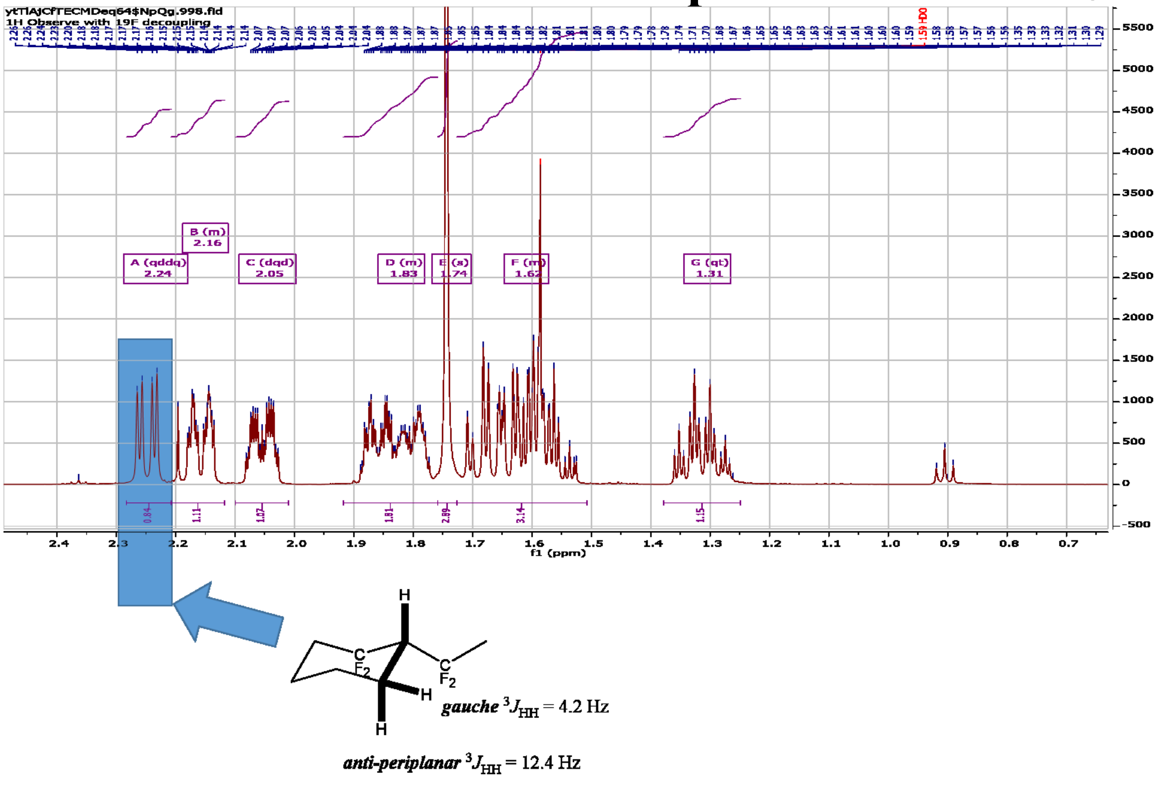

^{1}H-{^{19}F}NMR of **10** at Room temperature in $CDCl_3$.

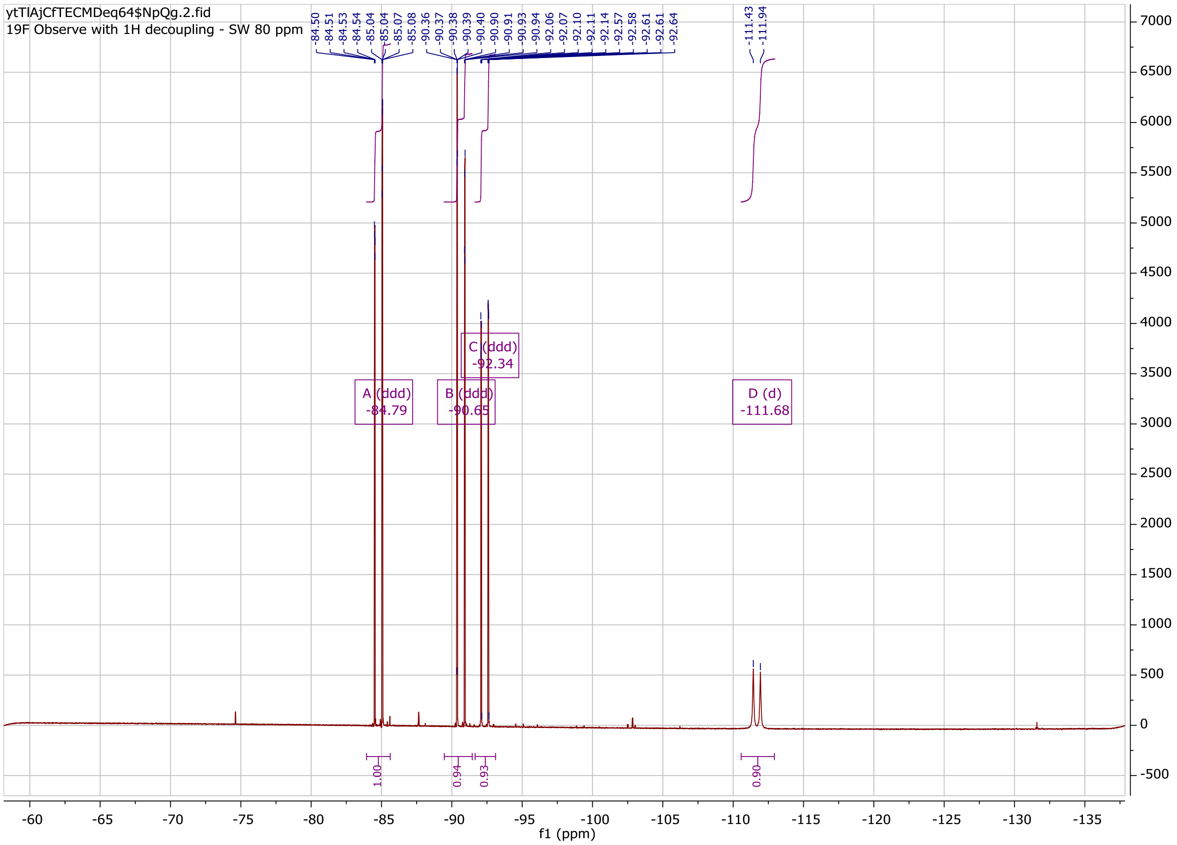

$^{19}F\{^{1}H\}$-NMR ($CDCl_3$) of 10 at room temperature (only one anomer apparent)

Protocol for synthesis of cyclohexane 11

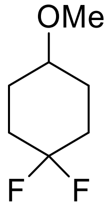

11

Deoxo-Fluor (6 cm^3) was added dropwise over 5 min to a solution of 4-methoxycyclohexanone (10 mmol) in toluene (5 cm^3) and the reaction was left to stir for 90 min monitoring progress by TLC. At completion the resulting solution was poured over aqueous sodium carbonate with additional solid sodium carbonate added until the pH of the mixture was basic monitoring using universal indicator paper. The product was extracted into DCM (x 3) and the combined organic layers washed with 1M HCl (aq), dried over solid anhydrous $MgSO_4$ and the solvent removed at reduced pressure. The product was purified over silica gel eluting with petroleum ether and diethyl ether (95:5 respectively) to yield cyclohexane **11** as a colourless liquid (1.02 g, 68 %).

The product had traces of 1-fluoro-4-methoxycyclohex-1-ene which were not removed by chromatography. Therefore ozone was bubbled through a cooled (-78°C) solution of the mixture (100 mg) in DCM (10ml) until a persistent blue colour was observed.

Dimethyl sulfide (2.3 cm^3) was added and the reaction left to stir for 2 h. The resulting solution was concentrated and purified over silica gel using petroleum ether and diethyl ether (95:5 respectively) as the eluent to give cyclohexane **11** as a colourless liquid (76 mg, 76 %).

^1H NMR (500 MHz CD_2Cl_2); 1.75 (m, 6H), 1.85 (m, 2H), 3.25 (s, 3H), 3.3 (m, 1H); $^{19}F\{^1H\}$-NMR (470MHz at CD_2Cl_2 -78°C); -103.93 (d, 230.0Hz, $1F_{OMe\ ax}$), -102.50 (d, 235.0Hz, $1F_{OMe\ eq}$), -94.14

(d, 230.3Hz, 1F$_{OMe\ eq}$), -88.14 (d, 230Hz, 1F($_{OMe\ ax}$). (see calculated spectra **Figure S5** for assignments)

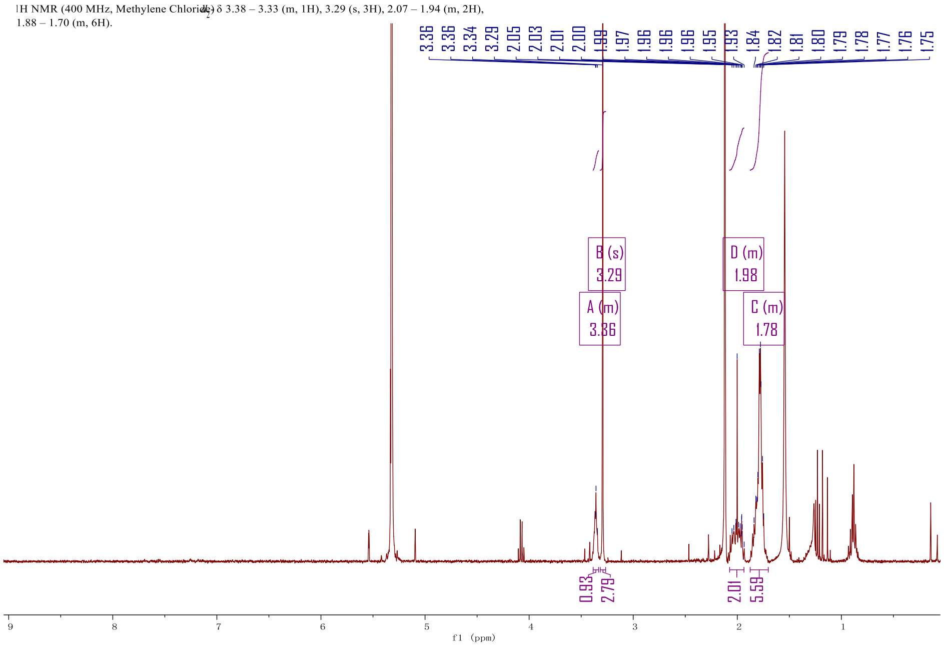

^{1}H-NMR of 11 at -80oC in CD_2Cl_2

^{19}F NMR (470 MHz, Methylene Chloride-d_2) δ -88.14 (d, J = 231.3 Hz), -94.12 (d, J = 230.7 Hz), -102.50 (d, J = 235.3 Hz), -103.93 (d, J = 231.6 Hz).

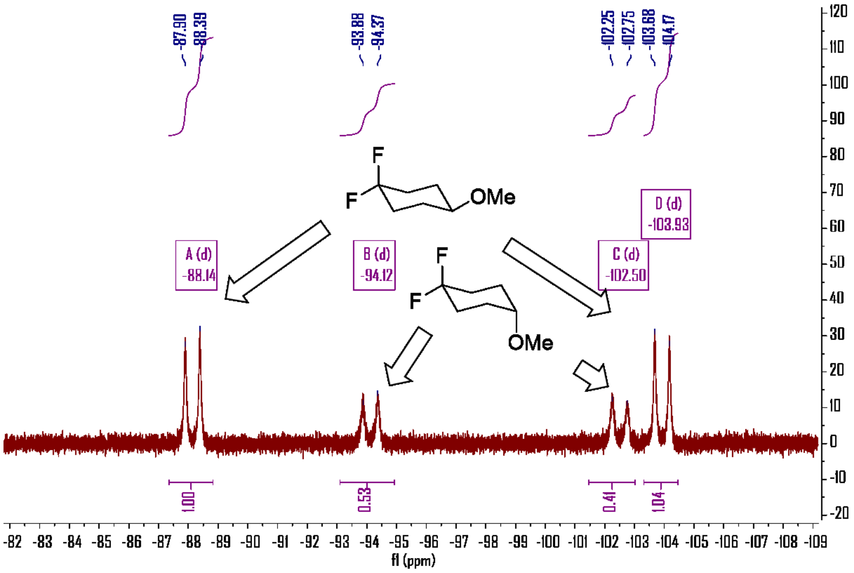

$^{19}F\{1H\}$-NMR (CD_2Cl_2 -80°C) of 11

(see **Figure S5** for calculated assignments as below)

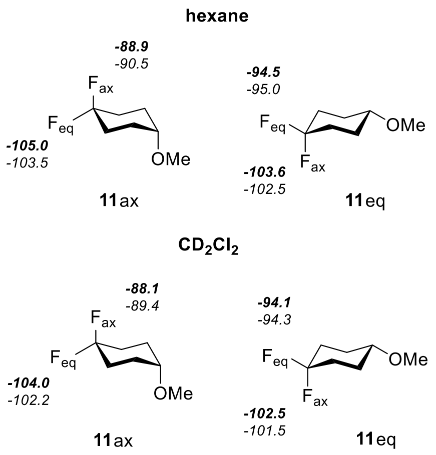

Computational details

All compounds were optimised at the M06-2X/aug-cc-pVTZ and harmonic frequency calculations were carried out at the same level in order to identify each conformer as true energy minima, showing no imaginary frequencies using the Gaussian16 Rev C.01 program.[1] The same harmonic frequency calculations were used to obtain enthalpies and Gibbs free energies at standard pressure and temperature for gas phase calculations. Optimisation and harmonic frequency calculations using the PCM solvent implicit model were run at the -80ºC experimental temperature. DLPNO-CCSD(T)/def2-TZVP single point calculations were ran using ORCA 4.2.1 package of programs.[2] NBO calculations, including the NPA, NCE and NSA analysis were done at the M06-2X/aug-cc-pVTZ level using the NBO 7.0 program.[3] QTAIM topological analysis was done on the electron densities obtained from the M06-2X/aug-cc-pVTZ optimised geometries through the AIMALL 19.10.12 program using 10^{-6} au envelope for integrations.[4] The quality of the integral properties were confirmed by the integrated values of the Laplacian of the charge density in each atom (that should be zero for an ideal integration), which were always lower than10^{-3} au. The NCI calculations were performed on the same M06-2X/aug-cc-pVTZ electron densities using the NCIPLOT 3.0 program.[5]

2 Results and Discussion

Table S1: Gas phase calculated total relative energy (Δ*E*), total relative enthalpy energy (Δ*H*) and total relative Gibbs free energy (Δ*G*) obtained at M06-2X/aug-cc-pVTZ theoretical level for compounds 1-15, in kcal mol^{-1}. Negative energy values represent axial preference, and the positive ones equatorial preference.

Compound	*ΔE*	*ΔH*	*ΔG*
**1**	-1.39	-1.21	-1.11
**8**	-1.36	-1.30	-1.34
**9**	1.95	2.12	2.26
**10**	1.06	1.18	0.93
**11**	-1.08	-1.00	-0.79
**12**	-2.28	-2.17	-1.96
**13**	-3.81	-3.83	-3.32
**14**	0.80	0.83	1.55
**15**	2.53	2.50	3.13

Table S2: NBO analysis relative energies (in kcal mol^{-1}) obtained at the M06-2X/aug-cc-pVTZ for compounds 1-15 . Negative values represent a preference for the axial conformer and positive ones for the equatorial conformer.

Compound	ΔE(T)	ΔE(L)	ΔE(NL)	ΔE(NCE)	ΔE(SX)
**1**	-1.39	-4.11	2.72	-0.41	1.39
**8**	-1.36	-9.16	7.80	-8.84	1.61
**9**	1.95	5.17	-3.23	-1.25	1.57
**10**	1.06	-0.09	1.15	-8.02	2.97
**11**	-1.08	-3.73	2.65	-3.50	3.38
**12**	-2.28	-10.61	8.33	-14.71	1.66
**13**	-3.81	-14.50	10.69	-28.34	0.81
**14**	0.80	1.01	-0.21	1.37	2.43
**15**	2.53	5.84	-3.31	5.54	0.62

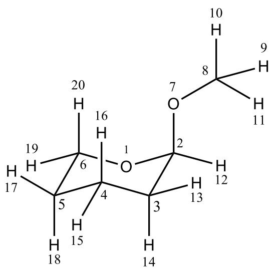

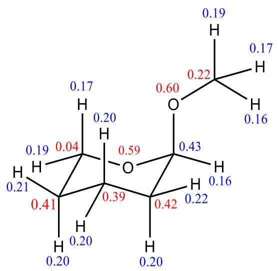

Table S3. Atom-atom electrostatic interactions (kcal mol^{-1}) obtained at M06-2X/aug-cc-pVTZ level using NPA charges for 1_{ax}.

	O1	C2	C3	C4	C5	C6	O7	C8	H9	H10	H11	H12	H13	H14	H15	H16	H17	H18	H19	H20
O1	-	-	-	-	-	-	-	-	-	-	-	-	-	-	-	-	-	-	-	-
C2	-60.19	-	-	-	-	-	-	-	-	-	-	-	-	-	-	-	-	-	-	-
C3	34.45	-39.94	-	-	-	-	-	-	-	-	-	-	-	-	-	-	-	-	-	-
C4	26.87	-22.36	36.20	-	-	-	-	-	-	-	-	-	-	-	-	-	-	-	-	-
C5	33.79	-20.78	23.60	35.59	-	-	-	-	-	-	-	-	-	-	-	-	-	-	-	-
C6	6.04	-2.63	2.15	2.28	3.98	-	-	-	-	-	-	-	-	-	-	-	-	-	-	-
O7	50.48	-60.77	35.62	26.41	23.69	3.04	-	-	-	-	-	-	-	-	-	-	-	-	-	-
C8	15.65	-13.56	8.59	6.83	6.88	0.94	31.28	-	-	-	-	-	-	-	-	-	-	-	-	-
H9	-13.37	9.24	-6.01	-5.08	-5.57	-0.83	-16.54	-11.77	-	-	-	-	-	-	-	-	-	-	-	-
H10	-9.64	8.17	-6.01	-5.04	-4.97	-0.64	-18.27	-12.70	6.03	-	-	-	-	-	-	-	-	-	-	-
H11	-10.29	9.02	-5.82	-4.38	-4.41	-0.58	-15.68	-11.09	5.32	5.70	-	-	-	-	-	-	-	-	-	-
H12	-15.43	20.31	-10.09	-5.85	-5.63	-0.69	-15.05	-4.48	2.98	2.73	3.63	-	-	-	-	-	-	-	-	-
H13	-12.65	14.39	-27.83	-12.86	-8.63	-0.81	-16.70	-4.11	2.74	3.02	2.85	4.38	-	-	-	-	-	-	-	-
H14	-14.77	13.67	-26.21	-12.24	-10.13	-0.90	-12.23	-3.38	2.45	2.38	2.41	4.34	8.20	-	-	-	-	-	-	-
H15	-10.40	8.43	-13.29	-24.32	-13.01	-0.86	-10.27	-2.86	2.12	2.17	1.89	2.45	5.72	5.55	-	-	-	-	-	-
H16	-12.25	10.45	-13.41	-24.37	-13.16	-1.07	-15.16	-3.84	2.80	2.95	2.35	2.76	5.91	4.51	7.86	-	-	-	-	-
H17	-12.08	7.65	-8.42	-12.36	-26.21	-1.39	-9.63	-2.94	2.41	2.22	1.88	2.19	3.42	3.67	5.49	5.66	-	-	-	-
H18	-14.23	8.55	-9.94	-11.81	-24.83	-1.33	-9.21	-2.79	2.26	2.02	1.87	2.51	3.69	5.05	5.34	4.35	7.61	-	-	-
H19	-18.27	8.16	-6.86	-7.03	-11.90	-2.48	-9.72	-3.34	3.09	2.34	2.14	2.41	2.74	3.09	2.93	3.34	5.06	4.93	-	-
H20	-15.73	8.88	-7.12	-7.76	-10.62	-2.19	-12.81	-4.07	3.74	2.87	2.31	2.36	2.91	2.81	2.95	4.31	4.60	3.52	5.77	-

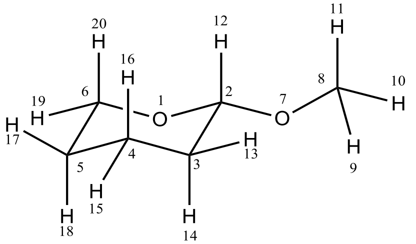

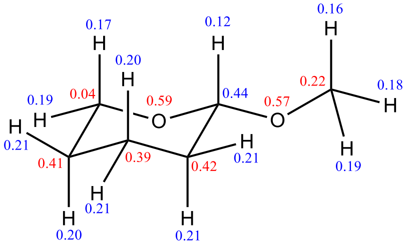

	O1	C2	C3	C4	C5	C6	O7	C8	H9	H10	H11	H12	H13	H14	H15	H16	H17	H18	H19	H20
O1	-	-	-	-	-	-	-	-	-	-	-	-	-	-	-	-	-	-	-	-
C2	-61.61	-	-	-	-	-	-	-	-	-	-	-	-	-	-	-	-	-	-	-
C3	34.83	-41.25	-	-	-	-	-	-	-	-	-	-	-	-	-	-	-	-	-	-
C4	26.94	-22.98	36.18	-	-	-	-	-	-	-	-	-	-	-	-	-	-	-	-	-
C5	34.12	-21.44	23.68	35.57	-	-	-	-	-	-	-	-	-	-	-	-	-	-	-	-
C6	5.28	-2.36	1.87	1.97	3.46	-	-	-	-	-	-	-	-	-	-	-	-	-	-	-
O7	49.50	-60.42	34.13	19.95	19.22	2.02	-	-	-	-	-	-	-	-	-	-	-	-	-	-
C8	15.86	-13.96	8.62	5.98	6.17	0.69	29.83	-	-	-	-	-	-	-	-	-	-	-	-	-
H9	-9.77	8.41	-6.02	-4.24	-4.33	-0.46	-17.43	-12.67	-	-	-	-	-	-	-	-	-	-	-	-
H10	-14.20	9.91	-6.30	-4.63	-5.11	-0.60	-16.55	-12.32	6.29	-	-	-	-	-	-	-	-	-	-	-
H11	-10.04	9.05	-5.70	-4.17	-4.24	-0.48	-14.60	-10.80	5.55	5.41	-	-	-	-	-	-	-	-	-	-
H12	-11.49	15.76	-7.77	-5.58	-5.11	-0.58	-11.00	-3.43	2.08	2.38	2.71	-	-	-	-	-	-	-	-	-
H13	-12.56	14.61	-27.63	-12.66	-8.55	-0.70	-15.37	-4.02	2.94	2.80	2.75	3.39	-	-	-	-	-	-	-	-
H14	-15.61	14.64	-27.34	-12.61	-10.48	-0.81	-15.64	-3.99	2.91	3.01	2.50	2.74	8.44	-	-	-	-	-	-	-
H15	-10.71	8.87	-13.62	-24.78	-13.34	-0.76	-8.73	-2.68	1.97	2.08	1.86	2.18	5.78	5.85	-	-	-	-	-	-
H16	-11.42	10.05	-12.54	-22.61	-12.34	-0.86	-8.91	-2.81	1.99	2.15	2.06	2.89	5.45	4.36	7.52	-	-	-	-	-
H17	-12.12	7.87	-8.43	-12.29	-26.25	-1.20	-7.57	-2.55	1.82	2.12	1.79	2.02	3.38	3.78	5.59	5.30	-	-	-	-
H18	-14.70	8.96	-10.16	-12.07	-25.57	-1.18	-8.78	-2.81	2.02	2.36	1.87	2.01	3.72	5.31	5.60	4.17	7.79	-	-	-
H19	-18.58	8.52	-6.98	-7.11	-12.12	-2.19	-8.37	-3.04	2.08	2.79	2.09	2.12	2.75	3.26	3.04	3.17	5.13	5.13	-	-
H20	-14.69	8.62	-6.67	-7.18	-9.89	-1.75	-7.52	-2.70	1.79	2.28	2.01	2.58	2.68	2.73	2.80	3.72	4.26	3.36	5.45	-

Table S4. Atom-atom electrostatic interactions (kcal mol^{-1}) obtained at M06-2X/aug-cc-pVTZ level using NPA charges for 1_{eq}.

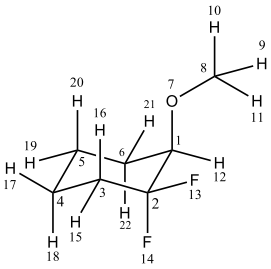

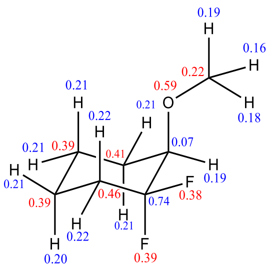

Table S5. Atom-atom electrostatic interactions (kcal mol^{-1}) obtained at M06-2X/aug-cc-pVTZ level using NPA charges for 8_{ax}.

	C1	C2	C3	C4	C5	C6	O7	C8	H9	H10	H11	H12	F13	F14	H15	H16	H17	H18	H19	H20	H21	H22
C1	-	-	-	-	-	-	-	-	-	-	-	-	-	-	-	-	-	-	-	-	-	-
C2	11.72	-	-	-	-	-	-	-	-	-	-	-	-	-	-	-	-	-	-	-	-	-
C3	-4.33	-75.13	-	-	-	-	-	-	-	-	-	-	-	-	-	-	-	-	-	-	-	-
C4	-3.17	-38.78	38.91	-	-	-	-	-	-	-	-	-	-	-	-	-	-	-	-	-	-	-
C5	-3.76	-33.47	23.78	33.46	-	-	-	-	-	-	-	-	-	-	-	-	-	-	-	-	-	-
C6	-6.40	-40.34	20.96	20.95	34.72	-	-	-	-	-	-	-	-	-	-	-	-	-	-	-	-	-
O7	-10.08	-60.74	30.20	21.73	26.47	33.46	-	-	-	-	-	-	-	-	-	-	-	-	-	-	-	-
C8	-2.18	-16.74	8.22	5.81	6.54	8.06	30.07	-	-	-	-	-	-	-	-	-	-	-	-	-	-	-
H9	1.47	10.83	-5.19	-3.86	-4.42	-5.79	-15.36	-10.65	-	-	-	-	-	-	-	-	-	-	-	-	-	-
H10	1.37	11.34	-6.15	-4.50	-5.09	-5.84	-18.24	-12.39	5.68	-	-	-	-	-	-	-	-	-	-	-	-	-
H11	1.59	14.75	-7.05	-4.68	-4.90	-5.84	-17.03	-11.92	5.46	6.33	-	-	-	-	-	-	-	-	-	-	-	-
H12	4.11	21.74	-8.25	-6.20	-7.05	-11.64	-17.80	-5.46	4.40	3.34	4.10	-	-	-	-	-	-	-	-	-	-	-
F13	-3.89	-69.41	24.80	13.48	12.05	14.01	26.94	9.24	-5.82	-6.19	-9.74	-9.16	-	-	-	-	-	-	-	-	-	-
F14	-3.99	-70.18	25.12	17.37	14.66	18.26	21.37	6.50	-4.59	-4.58	-5.68	-9.60	22.75	-	-	-	-	-	-	-	-	-
H15	1.55	26.05	-31.11	-13.24	-8.38	-7.66	-11.07	-3.32	2.15	2.54	2.97	3.28	-11.01	-11.19	-	-	-	-	-	-	-	-
H16	1.93	26.37	-31.29	-13.45	-10.49	-9.03	16.5	-4.47	2.67	3.48	3.82	3.71	-11.14	-8.87	9.38	-	-	-	-	-	-	-
H17	1.27	15.01	-14.74	-24.86	-12.58	-8.11	-9.55	-2.68	1.78	2.16	2.17	2.62	-5.91	-6.91	6.09	6.25	-	-	-	-	-	-
H18	1.44	17.93	-14.24	-23.93	-12.21	-9.75	-9.28	-2.62	1.81	2.02	2.14	3.02	-6.37	-9.85	6.01	4.90	7.94	-	-	-	-	-
H19	1.44	13.12	-9.09	-12.44	-24.82	-12.94	-10.43	-2.81	1.97	2.25	2.12	3.00	-5.07	-6.31	3.55	4.07	5.72	5.59	-	-	-	-
H20	1.79	15.47	-11.26	-12.42	-24.68	-12.94	-15.54	-3.73	2.43	3.03	2.71	3.39	-6.05	-6.38	4.02	5.87	5.75	4.51	8.06	-	-	-
H21	2.42	15.47	-8.28	-8.02	-12.89	-26.52	-16.21	-4.18	3.17	3.13	2.89	5.33	-6.09	-7.20	3.20	3.83	3.44	3.79	5.88	5.92	-	-
H22	2.37	18.95	-9.62	-9.85	-12.74	-25.99	-12.46	-3.42	2.54	2.49	2.60	5.36	-6.72	-10.58	3.78	3.92	3.86	5.42	5.82	4.72	8.49	-

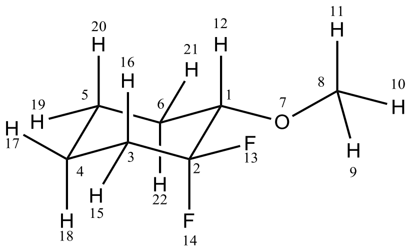 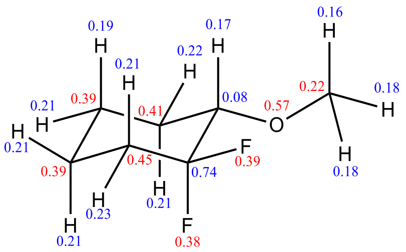

Table S6. Atom-atom electrostatic interactions (kcal mol^{-1}) obtained at M06-2X/aug-cc-pVTZ level using NPA charges for 8_{eq}.

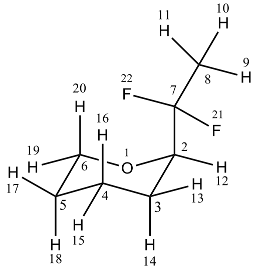

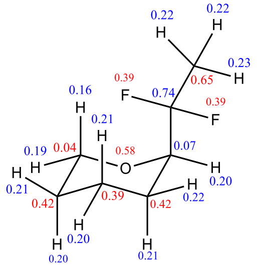

	O1	C2	C3	C4	C5	C6	C7	C8	H9	H10	H11	H12	H13	H14	H15	H16	H17	H18	H19	H20	F21	F22
O1	-	-	-	-	-	-	-	-	-	-	-	-	-	-	-	-	-	-	-	-	-	-
C2	-10.01	-	-	-	-	-	-	-	-	-	-	-	-	-	-	-	-	-	-	-	-	-
C3	33.27	-6.72	-	-	-	-	-	-	-	-	-	-	-	-	-	-	-	-	-	-	-	-
C4	26.29	-3.74	-36.13	-	-	-	-	-	-	-	-	-	-	-	-	-	-	-	-	-	-	-
C5	33.21	-3.91	-23.50	-35.60	-	-	-	-	-	-	-	-	-	-	-	-	-	-	-	-	-	-
C6	5.48	[illegible]	[illegible]	[illegible]	[illegible]	-	-	-	-	-	-	-	-	-	-	-	-	-	-	-	-	-
C7	-58.55	11.72	-40.48	-29.12	-26.78	-3.19	-	-	-	-	-	-	-	-	-	-	-	-	-	-	-	-
C8	44.06	-6.20	23.44	18.61	19.15	2.44	-106.59	-	-	-	-	-	-	-	-	-	-	-	-	-	-	-
H9	-16.94	1.96	-7.64	-6.50	-7.20	-1.00	26.31	-45.65	-	-	-	-	-	-	-	-	-	-	-	-	-	-
H10	-11.12	1.57	-6.75	-5.56	-5.61	-0.67	26.00	-44.84	9.58	-	-	-	-	-	-	-	-	-	-	-	-	-
H11	-13.97	1.94	-7.36	-5.62	-5.86	-0.72	25.52	-44.26	9.52	9.31	-	-	-	-	-	-	-	-	-	-	-	-
H12	-19.53	4.50	-13.21	-7.62	-7.40	-0.83	23.72	-15.61	4.69	4.04	5.74	-	-	-	-	-	-	-	-	-	-	-
H13	-12.81	2.53	-28.79	-13.40	-8.97	-0.78	20.51	-11.71	3.65	3.54	3.68	5.86	-	-	-	-	-	-	-	-	-	-
H14	-14.23	2.36	-26.45	-12.52	-10.17	-0.83	14.65	-9.56	3.17	2.78	3.17	5.93	8.68	-	-	-	-	-	-	-	-	-
H15	-10.24	1.43	-13.36	-24.53	13.13	-0.80	11.70	-7.89	2.74	2.43	2.45	3.23	5.96	5.80	-	-	-	-	-	-	-	-
H16	[illegible]	[illegible]	[illegible]	[illegible]	[illegible]	[illegible]	[illegible]	[illegible]	[illegible]	[illegible]	[illegible]	3.57	6.29	4.68	6.09	-	-	-	-	-	-	-
H17	-11.95	1.30	-8.46	-12.53	-26.50	-1.30	11.04	-8.27	3.15	2.51	2.52	2.88	3.59	3.73	5.57	5.86	-	-	-	-	-	-
H18	-14.14	1.47	-9.93	-11.90	-25.00	-1.24	10.78	-7.97	2.96	2.34	2.54	3.37	3.83	5.07	5.41	4.47	7.72	-	-	-	-	-
H19	-18.07	1.39	-6.85	-7.11	-12.02	-2.32	11.51	-9.71	4.12	2.75	3.01	3.15	2.87	3.09	2.97	3.44	5.17	4.99	-	-	-	-
H20	-15.32	1.48	-7.10	-7.79	-10.61	-2.02	14.21	-11.09	4.77	3.14	3.05	2.99	3.04	2.82	2.97	4.39	4.61	3.53	5.80	-	-	-
F21	20.89	-4.03	18.49	12.74	11.12	1.19	-69.17	35.75	-8.95	-11.28	-10.77	-10.60	-11.33	-7.09	-5.54	-7.09	-4.72	-4.68	-4.55	-5.10	-	-
F22	24.88	-3.98	19.29	17.19	15.14	1.71	-70.09	36.42	-11.43	-11.37	-8.83	-8.07	-10.02	-6.84	-6.79	-11.43	-6.57	-5.70	-6.05	-8.76	23.15	-

	C1	C2	C3	C4	C5	C6	O7	C8	H9	H10	H11	H12	F13	F14	H15	H16	H17	H18	H19	H20	H21	H22
C1	-	-	-	-	-	-	-	-	-	-	-	-	-	-	-	-	-	-	-	-	-	-
C2	12.69	-	-	-	-	-	-	-	-	-	-	-	-	-	-	-	-	-	-	-	-	-
C3	-4.69	-73.28	-	-	-	-	-	-	-	-	-	-	-	-	-	-	-	-	-	-	-	-
C4	-3.48	-38.38	38.72	-	-	-	-	-	-	-	-	-	-	-	-	-	-	-	-	-	-	-
C5	-4.06	-32.70	23.42	33.34	-	-	-	-	-	-	-	-	-	-	-	-	-	-	-	-	-	-
C6	[illegible]	[illegible]	[illegible]	[illegible]	34.40	-	-	-	-	-	-	-	-	-	-	-	-	-	-	-	-	-
O7	-10.77	-57.20	22.73	17.67	19.89	32.69	-	-	-	-	-	-	-	-	-	-	-	-	-	-	-	-
C8	-2.38	-17.25	7.26	5.36	5.71	7.93	29.07	-	-	-	-	-	-	-	-	-	-	-	-	-	-	-
H9	1.66	14.78	-6.09	-4.33	-4.34	-5.71	-16.34	-11.80	-	-	-	-	-	-	-	-	-	-	-	-	-	-
H10	1.48	11.03	-5.02	-3.88	-4.19	-5.73	-17.33	-12.15	6.15	-	-	-	-	-	-	-	-	-	-	-	-	-
H11	1.65	11.94	-5.38	-3.90	-4.20	-5.53	-14.78	-10.60	5.40	5.54	-	-	-	-	-	-	-	-	-	-	-	-
H12	4.10	19.78	-9.22	-6.63	-7.90	-10.64	-15.79	-4.75	3.22	3.00	4.02	-	-	-	-	-	-	-	-	-	-	-
F13	-4.29	-68.96	24.79	13.64	12.04	14.05	25.32	9.74	-9.52	-6.04	-7.11	-8.59	-	-	-	-	-	-	-	-	-	-
F14	-4.26	-68.37	24.37	16.94	14.03	17.46	26.28	7.87	-7.14	-5.40	-5.02	-6.63	22.44	-	-	-	-	-	-	-	-	-
H15	[illegible]	[illegible]	[illegible]	[illegible]	[illegible]	[illegible]	[illegible]	[illegible]	[illegible]	[illegible]	2.34	3.43	-11.34	-11.28	-	-	-	-	-	-	-	-
H16	1.99	24.50	-29.15	-12.77	-9.87	-8.55	-9.77	-3.29	2.70	2.27	2.58	4.62	10.68	-8.21	9.04	-	-	-	-	-	-	-
H17	1.39	14.84	-14.59	-25.01	-12.48	-8.11	-7.53	-2.40	1.95	1.77	1.78	2.83	-5.98	-6.76	6.22	5.91	-	-	-	-	-	-
H18	1.62	18.16	-14.42	-24.61	-12.40	-1-	-8.94	-2.69	2.21	1.98	1.89	2.91	-6.56	-9.80	6.23	4.74	8.14	-	-	-	-	-
H19	1.60	13.21	-9.20	-12.75	-25.12	-13.20	-8.92	-2.63	2.00	2.00	1.92	3.16	-5.21	-6.22	3.69	3.94	5.85	5.83	-	-	-	-
H20	1.79	14.12	-10.38	-11.58	-22.61	-11.92	-8.91	-2.70	2.03	1.98	2.09	4.12	-5.66	-5.73	3.80	5.18	5.34	4.28	7.64	-	-	-
H21	2.67	15.38	-8.27	-8.11	-12.84	-26.65	-15.92	-4.04	2.79	3.08	2.88	4.96	-6.17	-6.98	3.28	3.66	3.47	3.91	6.04	5.48	-	-
H22	2.65	18.97	-9.68	-10.05	-12.83	-26.36	-16.01	-3.91	2.92	2.92	2.58	3.99	-6.83	-10.20	3.91	3.77	3.93	5.65	6.04	4.43	8.72	-

Table S7. Atom-atom electrostatic interactions (kcal mol^{-1}) obtained at M06-2X/aug-cc-pVTZ level using NPA charges for 9_{ax}.

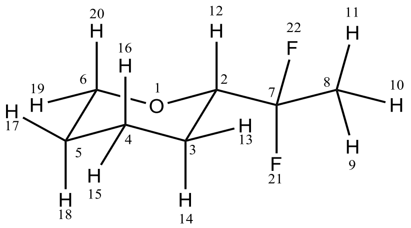
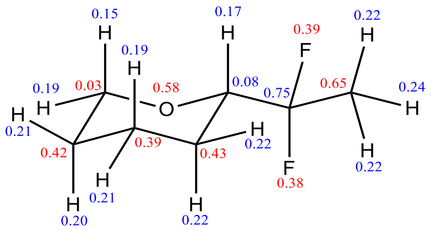

	O1	C2	C3	C4	C5	C6	C7	C8	H9	H10	H11	H12	H13	H14	H15	H16	H17	H18	H19	H20	F21	F22
O1	-	-	-	-	-	-	-	-	-	-	-	-	-	-	-	-	-	-	-	-	-	-
C2	-10.75	-	-	-	-	-	-	-	-	-	-	-	-	-	-	-	-	-	-	-	-	-
C3	33.88	-7.27	-	-	-	-	-	-	-	-	-	-	-	-	-	-	-	-	-	-	-	-
C4	26.00	-4.03	35.69	-	-	-	-	-	-	-	-	-	-	-	-	-	-	-	-	-	-	-
C5	33.43	-3.82	23.75	35.24	-	-	-	-	-	-	-	-	-	-	-	-	-	-	-	-	-	-
C6	4.04	-0.33	1.46	1.52	2.72	-	-	-	-	-	-	-	-	-	-	-	-	-	-	-	-	-
C7	-61.20	12.75	-41.58	-24.71	-24.41	-2.01	-	-	-	-	-	-	-	-	-	-	-	-	-	-	-	-
C8	44.52	-6.64	23.66	16.58	17.66	1.54	-107.66	-	-	-	-	-	-	-	-	-	-	-	-	-	-	-
H9	-11.31	1.68	-6.82	-4.85	-5.11	-0.42	26.27	-44.78	-	-	-	-	-	-	-	-	-	-	-	-	-	-
H10	-18.03	2.18	-7.97	-5.86	-6.65	-0.61	27.57	-47.21	9.90	-	-	-	-	-	-	-	-	-	-	-	-	-
H11	-13.32	2.02	-7.25	-5.33	-5.63	-0.50	25.19	-43.17	9.10	9.59	-	-	-	-	-	-	-	-	-	-	-	-
H12	-16.02	4.03	-11.17	-7.89	-7.29	-0.64	19.95	-13.15	3.38	4.15	4.71	-	-	-	-	-	-	-	-	-	-	-
H13	-12.75	2.68	-28.68	-13.02	-8.92	-0.57	19.70	-11.39	3.42	3.67	3.57	5.08	-	-	-	-	-	-	-	-	-	-
H14	-15.42	2.62	-27.86	-12.76	-10.78	-0.65	19.50	-11.32	3.38	3.94	3.30	4.00	8.96	-	-	-	-	-	-	-	-	-
H15	-10.49	1.58	-13.64	-24.56	-13.43	-0.60	11.05	-7.58	2.29	2.68	2.43	3.13	6.04	6.02	-	-	-	-	-	-	-	-
H16	-11.19	1.78	-12.49	-22.29	-12.34	-0.67	11.23	-7.85	2.30	2.73	2.64	4.15	5.65	4.45	7.53	-	-	-	-	-	-	-
H17	-11.95	1.41	-8.48	-12.23	-26.52	-0.95	9.71	-7.36	2.17	2.77	2.40	2.90	3.54	3.90	5.66	5.32	-	-	-	-	-	-
H18	-14.43	1.60	-10.25	-11.99	-25.78	-0.93	11.23	-8.13	2.40	3.12	2.51	2.89	3.91	5.50	5.65	4.18	7.89	-	-	-	-	-
H19	-18.28	1.52	-6.99	-7.05	-12.20	-1.72	10.79	-8.92	2.51	3.69	2.87	3.01	2.87	3.34	3.07	3.17	5.19	5.18	-	-	-	-
H20	-14.47	1.53	-6.66	-7.12	-9.95	-1.38	9.61	-7.70	2.12	2.92	2.67	3.63	2.79	2.79	2.83	3.73	4.31	3.39	5.48	-	-	-
F21	25.77	-4.18	19.00	11.51	11.71	0.90	-68.92	35.21	-11.00	-11.44	-8.34	-6.57	-8.85	-11.02	-5.44	-4.96	-4.66	-5.81	-5.06	-4.07	-	-
F22	21.12	-4.32	18.92	11.55	10.62	0.81	-69.79	35.66	-11.22	-9.25	-10.55	-8.69	-11.01	-8.54	-5.39	-5.50	-4.40	-4.83	-4.42	-4.11	22.33	-

Table S8. Atom-atom electrostatic interactions (kcal mol^{-1}) obtained at M06-2X/aug-cc-pVTZ level using NPA charges for 9_{eq}.

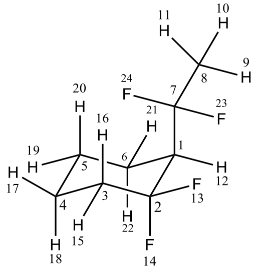

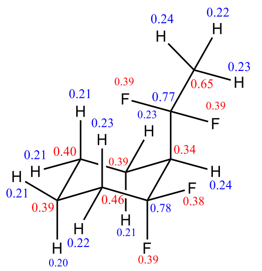

Table S9. Atom-atom electrostatic interactions (kcal mol^{-1}) obtained at

	C1	C2	C3	C4	C5	C6	C7	C8	H9	H10	H11	H12	F13	F14	H15	H16	H17	H18	H19	H20	H21	H22	F23	F24
C1	-	-	-	-	-	-	-	-	-	-	-	-	-	-	-	-	-	-	-	-	-	-	-	-
C2	-57.43	-	-	-	-	-	-	-	-	-	-	-	-	-	-	-	-	-	-	-	-	-	-	-
C3	20.19	-78.47	-	-	-	-	-	-	-	-	-	-	-	-	-	-	-	-	-	-	-	-	-	-
C4	14.71	-40.39	39.00	-	-	-	-	-	-	-	-	-	-	-	-	-	-	-	-	-	-	-	-	-
C5	17.39	-35.02	23.94	33.62	-	-	-	-	-	-	-	-	-	-	-	-	-	-	-	-	-	-	-	-
C6	28.88	-40.82	20.40	20.25	33.83	-	-	-	-	-	-	-	-	-	-	-	-	-	-	-	-	-	-	-
C7	-56.74	76.86	-35.71	-25.70	-30.98	-39.31	-	-	-	-	-	-	-	-	-	-	-	-	-	-	-	-	-	-
C8	28.30	-52.24	24.36	16.76	18.30	21.63	-110.63	-	-	-	-	-	-	-	-	-	-	-	-	-	-	-	-	-
H9	-7.31	13.79	-7.00	-5.05	-5.60	-6.35	27.37	-44.99	-	-	-	-	-	-	-	-	-	-	-	-	-	-	-	-
H10	-8.89	16.36	-7.26	-5.13	-5.57	-6.88	26.35	-43.94	9.32	-	-	-	-	-	-	-	-	-	-	-	-	-	-	-
H11	-9.02	20.09	-9.64	-6.19	-6.32	-7.05	28.13	-47.14	9.96	9.76	-	-	-	-	-	-	-	-	-	-	-	-	-	-
H12	-24.54	29.05	-10.49	-7.88	-8.95	-14.39	28.90	-18.74	4.85	6.95	5.78	-	-	-	-	-	-	-	-	-	-	-	-	-
F13	18.23	-72.55	24.93	13.51	12.08	13.58	-33.91	29.34	-7.46	-9.37	-13.11	-11.52	-	-	-	-	-	-	-	-	-	-	-	-
F14	18.93	-73.13	25.39	17.58	14.88	17.80	-26.85	19.53	-5.44	-6.63	-7.29	-12.63	22.93	-	-	-	-	-	-	-	-	-	-	-
H15	-7.30	27.46	-31.42	-13.35	-8.48	-7.50	13.55	-10.12	2.96	3.11	4.12	4.21	-11.23	-11.42	-	-	-	-	-	-	-	-	-	-
H16	-8.99	27.59	-31.74	-13.56	-10.57	-8.82	19.17	-13.22	3.88	3.71	5.40	4.67	-11.16	-8.98	9.56	-	-	-	-	-	-	-	-	-
H17	-5.95	15.76	-14.89	-25.02	-12.74	-7.91	11.51	-7.84	2.44	2.39	2.93	3.34	-5.97	-7.03	6.18	6.37	-	-	-	-	-	-	-	-
H18	-6.75	18.84	-14.40	-24.07	-12.36	-9.46	11.34	-7.71	2.32	2.45	2.83	3.91	-6.47	-10.09	6.14	4.99	8.05	-	-	-	-	-	-	-
H19	-6.74	13.83	-9.19	-12.56	-25.14	-12.71	12.63	-7.95	2.50	2.48	2.74	3.84	-5.12	-6.46	3.61	4.11	5.79	5.72	-	-	-	-	-	-
H20	-8.22	16.14	-11.34	-12.61	-25.26	-12.67	17.68	-10.23	3.23	2.99	3.52	4.25	-6.04	-6.47	4.08	5.88	5.92	4.62	8.28	-	-	-	-	-
H21	-11.86	17.01	-8.78	-8.46	-13.72	-27.24	21.45	-11.95	3.68	3.85	3.73	7.04	-6.41	-7.62	3.41	4.08	3.67	4.01	6.29	6.34	-	-	-	-
H22	-11.22	19.86	-9.70	-9.88	-12.98	-25.40	15.69	-9.78	2.89	3.27	3.27	7.08	-6.77	-10.69	3.84	3.97	3.91	5.44	6.02	4.83	9.09	-	-	-
F23	18.74	-26.76	13.30	10.70	13.88	18.19	-72.03	36.04	-11.76	-10.63	-9.28	-11.79	11.83	10.77	-5.29	-6.961	-4.94	-4.89	-6.11	-7.99	-12.37	-7.43	-	-
F24	18.56	-33.79	19.75	14.36	17.22	17.26	-73.07	36.40	-11.35	-8.77	-11.94	-9.44	14.79	11.86	-7.31	-12.35	-6.77	-5.91	-6.87	-11.53	-9.28	-6.90	23.13	-

M06-2X/aug-cc-pVTZ level using NPA charges for 10$_{ax}$.

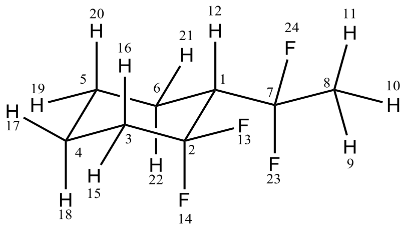

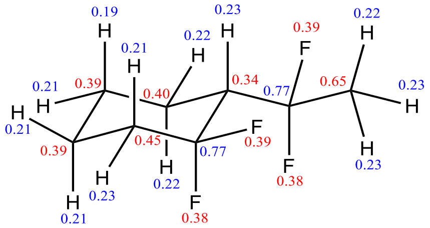

	C1	C2	C3	C4	C5	C6	C7	C8	H9	H10	H11	H12	F13	F14	H15	H16	H17	H18	H19	H20	H21	H22	F23	F24
C1	-	-	-	-	-	-	-	-	-	-	-	-	-	-	-	-	-	-	-	-	-	-	-	-
C2	-56.65	-	-	-	-	-	-	-	-	-	-	-	-	-	-	-	-	-	-	-	-	-	-	-
C3	19.70	-76.07	-	-	-	-	-	-	-	-	-	-	-	-	-	-	-	-	-	-	-	-	-	-
C4	14.78	-40.10	38.42	-	-	-	-	-	-	-	-	-	-	-	-	-	-	-	-	-	-	-	-	-
C5	16.98	-33.79	22.95	33.00	-	-	-	-	-	-	-	-	-	-	-	-	-	-	-	-	-	-	-	-
C6	28.95	-40.66	20.21	20.73	33.39	-	-	-	-	-	-	-	-	-	-	-	-	-	-	-	-	-	-	-
C7	-56.28	76.99	-29.31	-22.98	-25.44	-40.50	-	-	-	-	-	-	-	-	-	-	-	-	-	-	-	-	-	-
C8	27.89	-54.29	21.37	15.82	16.24	21.99	-111.09	-	-	-	-	-	-	-	-	-	-	-	-	-	-	-	-	-
H9	-7.21	14.07	-6.01	-4.65	-4.84	-6.45	27.45	-44.91	-	-	-	-	-	-	-	-	-	-	-	-	-	-	-	-
H10	-8.88	20.88	-8.00	-5.75	-5.58	-7.27	28.06	-46.61	9.85	-	-	-	-	-	-	-	-	-	-	-	-	-	-	-
H11	-8.74	17.41	-7.22	-5.20	-5.34	-6.85	26.53	-44.13	9.33	9.71	-	-	-	-	-	-	-	-	-	-	-	-	-	-
H12	-23.58	28.15	-12.30	-8.96	-10.57	-14.11	28.02	-17.25	4.57	5.26	6.27	-	-	-	-	-	-	-	-	-	-	-	-	-
F13	18.23	-72.44	24.73	13.70	11.95	13.78	-33.36	3-	-7.51	-12.37	-10.67	-11.71	-	-	-	-	-	-	-	-	-	-	-	-
F14	18.19	-72.16	24.36	16.90	13.81	17.05	-34.18	24.98	-6.85	-10.61	-7.31	-9.05	22.69	-	-	-	-	-	-	-	-	-	-	-
H15	-7.36	27.65	-31.07	-13.59	-8.40	-7.68	12.47	-9.69	2.78	3.78	3.27	4.64	-11.42	-11.49	-	-	-	-	-	-	-	-	-	-
H16	-8.43	25.85	-29.00	-12.85	-9.81	-8.40	12.85	-9.66	2.73	3.50	3.44	6.21	-10.90	-8.33	9.10	-	-	-	-	-	-	-	-	-
H17	-5.92	15.61	-14.56	-25.18	-12.40	-8.02	9.92	-7.12	2.14	2.59	2.38	3.84	-6.04	-6.81	6.28	5.98	-	-	-	-	-	-	-	-
H18	-6.86	18.89	-14.25	-24.59	-12.24	-9.85	11.60	-8.02	2.39	2.98	2.55	3.93	-6.54	-9.68	6.24	4.75	8.18	-	-	-	-	-	-	-
H19	-6.82	13.85	-9.14	-12.81	-24.90	-13.02	11.65	-7.62	2.35	2.63	2.48	4.30	-5.24	-6.21	3.71	3.97	5.90	5.83	-	-	-	-	-	-
H20	-7.60	14.83	-10.30	-11.57	-22.32	-11.68	11.65	-7.72	2.30	2.60	2.64	5.58	-5.72	-5.74	3.82	5.22	5.35	4.27	7.66	-	-	-	-	-
H21	-11.69	16.74	-8.53	-8.48	-13.25	-27.26	21.33	-11.67	3.59	3.69	3.68	6.96	-6.42	-7.27	3.43	3.81	3.64	4.09	6.35	5.70	-	-	-	-
H22	-11.34	20.05	-9.78	-10.33	-12.98	-26.38	20.41	-11.46	3.46	3.95	3.41	5.48	-6.90	-10.22	4.00	3.85	4.05	5.82	6.21	4.52	9.27	-	-	-
F23	17.97	-31.29	12.79	10.75	11.61	18.33	-71.56	35.42	-11.10	-11.49	-8.57	-8.97	13.00	17.19	-5.66	-5.36	-4.65	-5.82	-5.62	-5.03	-9.61	-11.45	-	-
F24	18.80	-27.06	11.98	9.95	11.78	17.96	-72.83	36.26	-11.74	-9.28	-10.79	-12.22	12.25	11.93	-5.13	-5.53	-4.46	-4.94	-5.63	-5.67	-11.65	-8.61	22.73	-

Table S10. Atom-atom electrostatic interactions (kcal mol^{-1}) obtained at M06-2X/aug-cc-pVTZ level using NPA charges for 10_{eq}.

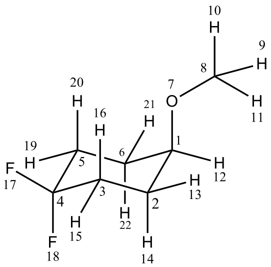 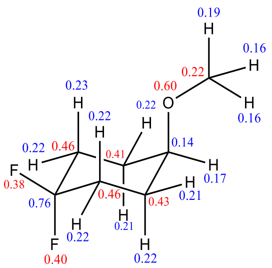

Table S11. Atom-atom electrostatic interactions (kcal mol^{-1}) obtained at M06-2X/aug-cc-pVTZ level using NPA charges for 11_{ax}.

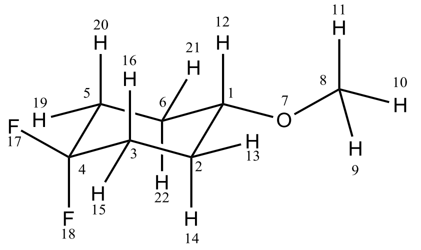

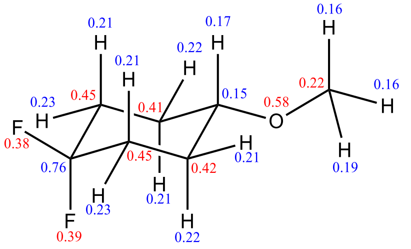

	C1	C2	C3	C4	C5	C6	O7	C8	H9	H10	H11	H12	H13	H14	H15	H16	F17	F18	H19	H20	H21	H22
C1	-	-	-	-	-	-	-	-	-	-	-	-	-	-	-	-	-	-	-	-	-	-
C2	-12.64	-	-	-	-	-	-	-	-	-	-	-	-	-	-	-	-	-	-	-	-	-
C3	-8.24	42.43	-	-	-	-	-	-	-	-	-	-	-	-	-	-	-	-	-	-	-	-
C4	11.89	-43.39	-76.77	-	-	-	-	-	-	-	-	-	-	-	-	-	-	-	-	-	-	-
C5	-8.22	21.93	27.36	-76.58	-	-	-	-	-	-	-	-	-	-	-	-	-	-	-	-	-	-
C6	-12.07	22.89	20.88	-41.13	40.17	-	-	-	-	-	-	-	-	-	-	-	-	-	-	-	-	-
O7	-19.08	34.50	30.63	-43.69	31.21	34.09	-	-	-	-	-	-	-	-	-	-	-	-	-	-	-	-
C8	-4.18	10.47	9.20	-12.27	7.84	8.05	30.86	-	-	-	-	-	-	-	-	-	-	-	-	-	-	-
H9	2.86	-7.14	-5.83	7.99	-5.10	-5.54	-15.59	-10.81	-	-	-	-	-	-	-	-	-	-	-	-	-	-
H10	2.65	-6.81	-6.58	9.28	-6.06	-5.91	-18.80	-12.79	5.83	-	-	-	-	-	-	-	-	-	-	-	-	-
H11	2.61	-8.19	-7.52	9.18	-5.41	-5.21	-15.25	-10.64	4.87	5.74	-	-	-	-	-	-	-	-	-	-	-	-
H12	7.11	-11.30	-7.55	11.16	-7.52	-10.74	-16.61	-4.80	4.00	3.11	2.90	-	-	-	-	-	-	-	-	-	-	-
H13	4.39	-27.30	-14.77	15.48	-8.06	-8.17	-15.17	-5.66	4.01	3.57	4.99	4.82	-	-	-	-	-	-	-	-	-	-
H14	4.62	-28.24	-15.34	19.98	-9.94	-10.68	-12.83	-4.03	2.95	2.78	3.07	5.08	8.68	-	-	-	-	-	-	-	-	-
H15	2.93	-14.58	-31.32	26.82	-9.86	-7.70	-11.19	-3.68	2.42	2.71	3.10	2.98	6.19	6.51	-	-	-	-	-	-	-	-
H16	3.59	-14.45	-30.78	26.50	-11.95	-8.87	-16.38	-5.06	2.97	3.71	4.32	3.33	6.15	5.21	9.31	-	-	-	-	-	-	-
F17	-4.16	14.63	24.63	-70.78	24.54	13.89	17.09	5.12	-3.32	-4.06	-3.89	-4.19	-5.91	-6.86	-10.97	-10.91	-	-	-	-	-	-
F18	-5.13	19.30	25.65	-72.97	25.54	18.23	17.70	5.26	-3.62	-3.99	-3.92	-5.30	-7.11	-10.91	-11.60	-8.90	23.00	-	-	-	-	-
H19	2.92	-8.09	-9.86	26.75	-31.10	-13.82	-11.37	-3.09	2.07	2.46	2.14	2.97	3.15	3.95	3.94	4.35	-10.91	-11.56	-	-	-	-
H20	3.68	-9.56	-12.30	27.23	-31.56	-14.14	-17.25	-4.24	2.63	3.41	2.89	3.41	3.77	4.10	4.48	6.33	-11.22	-9.14	9.53	-	-	-
H21	4.60	-8.92	-8.40	16.00	-15.25	-26.85	-16.80	-4.08	2.87	3.16	2.52	5.00	3.53	4.20	3.27	3.83	-6.12	-7.30	6.35	6.63	-	-
H22	4.47	-10.82	-9.58	19.18	-14.82	-26.04	-12.68	-3.42	2.48	2.54	2.30	4.93	3.93	6.00	3.80	3.86	-6.61	-10.41	6.32	5.20	8.64	-

Table S12. Atom-atom electrostatic interactions (kcal mol^{-1}) obtained at M06-2X/aug-cc-pVTZ level using NPA charges for 11_{eq}.

	C1	C2	C3	C4	C5	C6	O7	C8	H9	H10	H11	H12	H13	H14	H15	H16	F17	F18	H19	H20	H21	H22
C1	-	-	-	-	-	-	-	-	-	-	-	-	-	-	-	-	-	-	-	-	-	-
C2	-12.85	-	-	-	-	-	-	-	-	-	-	-	-	-	-	-	-	-	-	-	-	-
C3	-8.29	41.60	-	-	-	-	-	-	-	-	-	-	-	-	-	-	-	-	-	-	-	-
C4	12.09	-42.95	-75.50	-	-	-	-	-	-	-	-	-	-	-	-	-	-	-	-	-	-	-
C5	-8.31	21.61	26.76	-75.74	-	-	-	-	-	-	-	-	-	-	-	-	-	-	-	-	-	-
C6	-12.35	22.82	20.67	-41.17	39.96	-	-	-	-	-	-	-	-	-	-	-	-	-	-	-	-	-
O7	-19.00	33.36	22.95	-34.84	23.37	33.17	-	-	-	-	-	-	-	-	-	-	-	-	-	-	-	-
C8	-4.27	10.33	7.47	-10.69	6.74	8.07	29.93	-	-	-	-	-	-	-	-	-	-	-	-	-	-	-
H9	2.70	-8.18	-5.72	7.83	-4.64	-5.26	-14.94	-10.75	-	-	-	-	-	-	-	-	-	-	-	-	-	-
H10	2.70	-6.74	-5.26	7.89	-5.03	-5.92	-18.21	-12.75	5.78	-	-	-	-	-	-	-	-	-	-	-	-	-
H11	2.87	-6.86	-5.37	7.60	-4.84	-5.49	-14.90	-10.63	4.84	5.72	-	-	-	-	-	-	-	-	-	-	-	-
H12	6.40	-9.99	-8.26	11.74	-8.26	-9.56	-14.30	-4.30	2.65	2.75	3.52	-	-	-	-	-	-	-	-	-	-	-
H13	4.47	-27.10	-14.60	15.42	-7.98	-8.16	-14.68	-5.57	5.01	3.52	3.81	4.28	-	-	-	-	-	-	-	-	-	-
H14	4.66	-27.76	-14.90	19.53	-9.68	-10.53	-15.57	-4.71	3.86	3.28	2.93	3.58	8.50	-	-	-	-	-	-	-	-	-
H15	3.03	-14.72	-31.27	27.14	-9.91	-7.84	-9.50	-3.25	2.59	2.36	2.32	3.05	6.28	6.52	-	-	-	-	-	-	-	-
H16	3.51	-13.82	-29.15	25.48	-11.40	-8.54	-9.92	-3.38	2.54	2.39	2.59	4.14	5.97	4.95	9.11	-	-	-	-	-	-	-
F17	-4.24	14.54	24.28	-70.65	24.33	13.92	13.32	4.32	-3.18	-3.27	-3.15	-4.48	-5.91	-6.74	-11.11	-10.55	-	-	-	-	-	-
F18	-5.15	18.83	24.93	-72.06	24.99	18.02	16.59	5.08	-3.79	-3.85	-3.47	-4.71	-6.96	-10.48	-11.59	-8.46	22.69	-	-	-	-	-
H19	3.04	-8.19	-9.90	27.19	-31.42	-14.12	-9.69	-2.87	1.98	2.22	2.04	3.05	3.20	3.95	4.07	4.26	-11.10	-11.62	-	-	-	-
H20	3.50	-8.88	-11.34	25.45	-29.21	-13.21	-10.03	-3.05	2.07	2.27	2.30	4.11	3.53	3.77	4.25	5.69	-10.55	-8.45	9.12	-	-	-
H21	4.69	-8.87	-8.27	15.95	-15.07	-26.89	-16.22	-4.09	2.55	3.15	2.86	4.46	3.52	4.15	3.32	3.67	-6.11	-7.20	6.47	6.14	-	-
H22	4.66	-11.00	-9.66	19.53	-14.94	-26.52	-16.08	-3.96	2.68	3.02	2.55	3.58	3.99	6.02	3.94	3.78	-6.73	-10.46	6.53	4.94	8.81	-

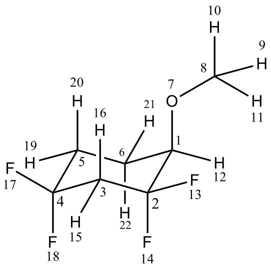

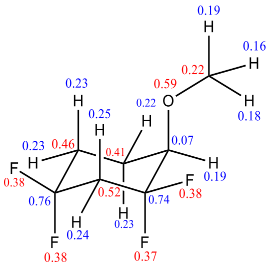

Table S13. Atom-atom electrostatic interactions (kcal mol^{-1}) obtained at M06-2X/aug-cc-pVTZ level using NPA charges for 12_{ax}.

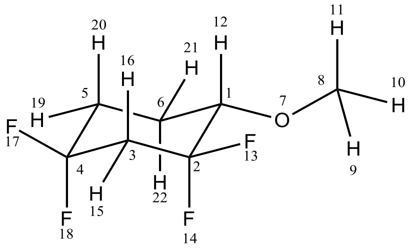

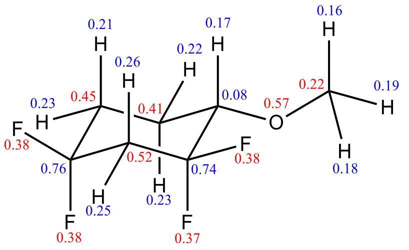

	C1	C2	C3	C4	C5	C6	O7	C8	H9	H10	H11	H12	F13	F14	H15	H16	F17	F18	H19	H20	H21	H22
C1	-	-	-	-	-	-	-	-	-	-	-	-	-	-	-	-	-	-	-	-	-	-
C2	11.81	-	-	-	-	-	-	-	-	-	-	-	-	-	-	-	-	-	-	-	-	-
C3	-5.00	-85.66	-	-	-	-	-	-	-	-	-	-	-	-	-	-	-	-	-	-	-	-
C4	6.30	75.24	-87.65	-	-	-	-	-	-	-	-	-	-	-	-	-	-	-	-	-	-	-
C5	-4.41	-38.58	31.38	-76.68	-	-	-	-	-	-	-	-	-	-	-	-	-	-	-	-	-	-
C6	-6.56	-40.98	24.41	-41.89	40.97	-	-	-	-	-	-	-	-	-	-	-	-	-	-	-	-	-
O7	-10.21	-60.90	34.69	-43.38	31.23	34.08	-	-	-	-	-	-	-	-	-	-	-	-	-	-	-	-
C8	-2.21	-16.72	9.40	-11.50	7.68	8.22	30.17	-	-	-	-	-	-	-	-	-	-	-	-	-	-	-
H9	1.50	10.91	-6.00	7.71	-5.26	-5.97	-15.61	-10.83	-	-	-	-	-	-	-	-	-	-	-	-	-	-
H10	1.41	11.53	-7.16	9.07	-6.07	-6.03	-18.58	-12.64	5.85	-	-	-	-	-	-	-	-	-	-	-	-	-
H11	1.62	14.80	-8.08	9.26	-5.75	-5.98	-17.18	-12.03	5.57	6.47	-	-	-	-	-	-	-	-	-	-	-	-
H12	4.31	22.58	-9.80	12.58	-8.52	-12.28	-18.50	-5.68	4.60	3.52	4.32	-	-	-	-	-	-	-	-	-	-	-
F13	-3.86	-68.31	28.08	-25.95	13.76	14.00	26.67	9.08	-5.74	-6.20	-9.60	-9.30	-	-	-	-	-	-	-	-	-	-
F14	-3.87	-67.78	27.56	-31.70	16.10	17.76	20.61	6.26	-4.46	-4.48	-5.52	-9.61	21.51	-	-	-	-	-	-	-	-	-
H15	1.71	28.22	-39.11	28.87	-10.72	-8.61	-12.08	-3.60	2.36	2.80	3.21	3.72	-11.61	-11.83	-	-	-	-	-	-	-	-
H16	2.12	28.78	-39.45	29.42	-13.19	-9.96	-17.92	-4.87	2.95	3.86	4.18	4.19	-12.21	-9.36	11.25	-	-	-	-	-	-	-
F17	-2.19	-25.24	27.99	-69.83	24.23	13.94	16.90	4.77	-3.19	-3.95	-3.87	-4.68	10.09	11.01	-11.52	-12.18	-	-	-	-	-	-
F18	-2.58	-31.41	28.18	-70.58	24.62	17.76	16.78	4.78	-3.35	-3.75	-3.92	-5.68	11.27	16.71	-12.18	-9.54	21.82	-	-	-	-	-
H19	1.61	14.56	-11.62	27.56	-32.09	-14.48	-11.68	-3.14	2.23	2.55	2.37	3.45	-5.55	-6.67	4.40	4.95	-10.99	-11.50	-	-	-	-
H20	2.02	17.15	-14.22	27.54	-32.04	-14.58	-17.67	-4.22	2.78	3.49	3.06	3.93	-6.65	-6.77	4.89	7.02	-11.25	-8.91	9.94	-	-	-
H21	2.52	16.00	-9.80	16.34	-15.59	-27.92	-16.70	-4.33	3.34	3.28	3.01	5.75	-6.19	-7.16	3.65	4.30	-6.16	-7.17	6.71	6.81	-	-
H22	2.56	20.47	-11.96	20.76	-15.89	-28.41	-13.41	-3.67	2.76	2.72	2.82	5.94	-7.10	-10.91	4.55	4.62	-7.00	-10.75	6.92	5.64	9.45	-

Table S14. Atom-atom electrostatic interactions (kcal mol^{-1}) obtained at M06-2X/aug-cc-pVTZ level using NPA charges for 12_{eq}.

	C1	C2	C3	C4	C5	C6	O7	C8	H9	H10	H11	H12	F13	F14	H15	H16	F17	F18	H19	H20	H21	H22
C1		-	-	-	-	-	-	-	-	-	-	-	-	-	-	-	-	-	-	-	-	-
C2	12.98	-	-	-	-	-	-	-	-	-	-	-	-	-	-	-	-	-	-	-	-	-
C3	-5.49	-83.91	-	-	-	-	-	-	-	-	-	-	-	-	-	-	-	-	-	-	-	-
C4	6.96	73.99	-86.84	-	-	-	-	-	-	-	-	-	-	-	-	-	-	-	-	-	-	-
C5	-4.83	-37.70	30.93	-75.87	-	-	-	-	-	-	-	-	-	-	-	-	-	-	-	-	-	-
C6	-7.29	-40.36	24.25	-41.84	40.64	-	-	-	-	-	-	-	-	-	-	-	-	-	-	-	-	-
O7	-10.96	-56.85	25.88	-34.06	22.96	33.04	-	-	-	-	-	-	-	-	-	-	-	-	-	-	-	-
C8	-2.46	-17.53	8.42	-10.51	6.68	8.12	29.02	-	-	-	-	-	-	-	-	-	-	-	-	-	-	-
H9	1.71	15.05	-7.07	8.48	-5.08	-5.86	-16.31	-11.96	-	-	-	-	-	-	-	-	-	-	-	-	-	-
H10	1.55	11.29	-5.87	7.68	-4.95	-5.92	-17.50	-12.45	6.29	-	-	-	-	-	-	-	-	-	-	-	-	-
H11	1.71	12.20	-6.25	7.69	-4.93	-5.66	-14.81	-10.79	5.49	5.70	-	-	-	-	-	-	-	-	-	-	-	-
H12	4.22	19.90	-10.61	13.14	-9.32	-10.88	-15.78	-4.78	3.24	3.07	4.05	-	-	-	-	-	-	-	-	-	-	-
F13	-4.32	-68.12	28.25	-26.18	13.80	14.09	24.60	9.69	-9.39	-6.06	-7.20	-8.58	-	-	-	-	-	-	-	-	-	-
F14	-4.19	-66.12	26.82	-30.63	15.37	16.96	25.23	7.78	-7.14	-5.36	-4.96	-6.43	21.30	-	-	-	-	-	-	-	-	-
H15	1.93	28.43	-39.34	29.25	-10.79	-8.74	-10.25	-3.50	3.07	2.50	2.57	3.74	-11.95	-11.91	-	-	-	-	-	-	-	-
H16	2.23	27.19	-37.42	28.15	-12.56	-9.54	-10.71	-3.67	3.02	2.57	2.88	5.06	-11.95	-8.80	10.98	-	-	-	-	-	-	-
F17	-2.42	-24.92	27.73	-69.66	23.99	13.93	12.86	4.20	-3.41	-3.15	-3.15	-4.98	10.22	10.72	-11.69	-11.68	-	-	-	-	-	-
F18	-2.84	-30.64	27.61	-69.76	24.12	17.58	15.54	4.79	-3.95	-3.59	-3.37	-5.07	11.20	15.92	-12.19	-9.04	21.54	-	-	-	-	-
H19	1.82	14.62	-11.74	27.99	-32.37	-14.75	-9.78	-2.93	2.24	2.25	2.14	3.54	-5.71	-6.54	4.55	4.84	-11.18	-11.55	-	-	-	-
H20	2.06	15.75	-13.18	25.64	-29.53	-13.51	-9.94	-3.06	2.30	2.27	2.37	4.70	-6.29	-6.11	4.64	6.29	-10.50	-8.21	9.46	-	-	-
H21	2.84	16.01	-9.86	16.51	-15.61	-28.23	-16.53	-4.23	2.92	3.26	3.00	5.17	-6.33	-6.98	3.76	4.17	-6.22	-7.18	6.91	6.37	-	-
H22	2.90	20.42	-12.02	21.01	-15.95	-28.72	-16.88	-4.20	3.16	3.17	2.77	4.30	-7.20	-10.46	4.68	4.48	-7.09	-10.79	7.13	5.31	9.72	-

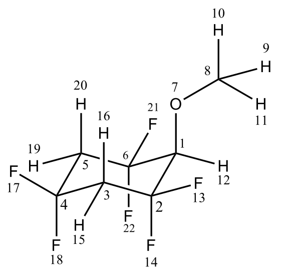

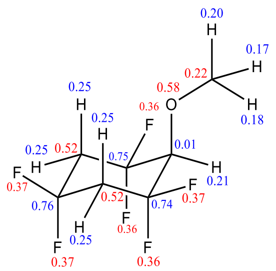

Table S15. Atom-atom electrostatic interactions (kcal mol^{-1}) obtained at M06-2X/aug-cc-pVTZ level using NPA charges for 13$_{ax}$.

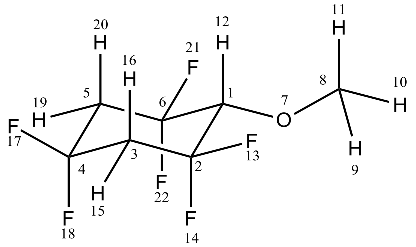

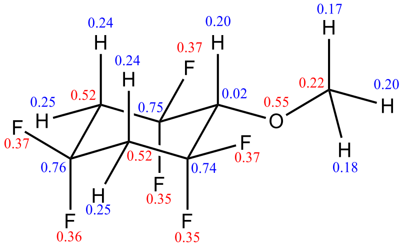

	C1	C2	C3	C4	C5	C6	O7	C8	H9	H10	H11	H12	F13	F14	H15	H16	F17	F18	H19	H20	F21	F22
C1	-	-	-	-	-	-	-	-	-	-	-	-	-	-	-	-	-	-	-	-	-	-
C2	1.67	-	-	-	-	-	-	-	-	-	-	-	-	-	-	-	-	-	-	-	-	-
C3	-0.71	-85.54	-	-	-	-	-	-	-	-	-	-	-	-	-	-	-	-	-	-	-	-
C4	0.89	75.00	-87.40	-	-	-	-	-	-	-	-	-	-	-	-	-	-	-	-	-	-	-
C5	-0.71	-43.89	35.74	-87.40	-	-	-	-	-	-	-	-	-	-	-	-	-	-	-	-	-	-
C6	1.68	73.65	-44.17	75.55	-86.00	-	-	-	-	-	-	-	-	-	-	-	-	-	-	-	-	-
O7	-1.43	-60.29	34.35	-42.52	34.64	-61.12	-	-	-	-	-	-	-	-	-	-	-	-	-	-	-	-
C8	-0.31	-16.11	9.07	-11.26	8.70	-15.15	29.23	-	-	-	-	-	-	-	-	-	-	-	-	-	-	-
H9	0.22	11.04	-6.19	8.13	-6.50	11.95	-16.11	-11.31	-	-	-	-	-	-	-	-	-	-	-	-	-	-
H10	0.21	11.75	-7.29	9.24	-7.09	11.38	-18.65	-12.85	6.31	-	-	-	-	-	-	-	-	-	-	-	-	-
H11	0.23	14.06	-7.54	8.87	-6.41	10.97	-16.73	-11.80	5.79	6.57	-	-	-	-	-	-	-	-	-	-	-	-
H12	0.67	24.56	-10.73	13.82	-10.75	24.77	-19.77	-6.23	5.16	3.96	4.97	-	-	-	-	-	-	-	-	-	-	-
F13	-0.54	-67.15	27.47	-25.39	15.42	-24.92	26.34	8.50	-5.54	-6.24	-8.66	-9.92	-	-	-	-	-	-	-	-	-	-
F14	-0.52	-65.48	26.40	-30.22	17.54	-30.12	19.44	5.87	-4.36	-4.40	-5.18	-10.03	20.25	-	-	-	-	-	-	-	-	-
H15	0.25	28.97	-40.07	29.64	-12.57	15.95	-12.26	-3.57	2.49	2.93	3.08	4.17	-11.60	-11.67	-	-	-	-	-	-	-	-
H16	0.31	29.21	-39.96	29.78	-15.26	18.46	-18.13	-4.74	3.09	3.98	3.88	4.66	-12.19	-9.12	11.70	-	-	-	-	-	-	-
F17	-0.31	-24.89	27.57	-68.84	27.56	-25.03	16.45	4.63	-3.34	-3.99	-3.65	-5.08	9.76	10.37	-11.62	-12.23	-	-	-	-	-	-
F18	-0.35	-30.02	27.04	-68.15	27.04	-30.27	15.76	4.51	-3.38	-3.68	-3.65	-5.97	10.56	15.17	-12.05	-9.31	20.70	-	-	-	-	-
H19	0.25	15.84	-12.57	29.64	-40.07	29.11	-12.35	-3.40	2.64	2.84	2.54	4.18	-5.93	-6.96	4.90	5.42	-11.62	-12.04	-	-	-	-
H20	0.31	18.46	-15.32	29.85	-40.09	29.45	-18.44	-4.52	3.28	3.86	3.21	4.70	-7.07	-7.01	5.44	7.66	-12.24	-9.33	11.74	-	-	-
F21	-0.53	-24.32	15.11	-24.93	26.92	-66.43	25.99	7.29	-6.31	-5.65	-4.99	-9.80	9.52	10.32	-5.82	-6.87	9.56	10.40	-11.41	-11.92	-	-
F22	-0.53	-30.29	17.76	-30.60	26.51	-65.92	19.60	5.64	-4.60	-4.30	-4.36	-10.09	10.58	15.71	-7.06	-7.04	10.46	15.44	-11.66	-9.17	19.94	-

Table S16. Atom-atom electrostatic interactions (kcal mol^{-1}) obtained at M06-2X/aug-cc-pVTZ level using NPA charges for 13$_{eq}$.

	C1	C2	C3	C4	C5	C6	O7	C8	H9	H10	H11	H12	F13	F14	H15	H16	F17	F18	H19	H20	F21	F22
C1	-	-	-	-	-	-	-	-	-	-	-	-	-	-	-	-	-	-	-	-	-	-
C2	2.71	-	-	-	-	-	-	-	-	-	-	-	-	-	-	-	-	-	-	-	-	-
C3	-1.14	-84.14	-	-	-	-	-	-	-	-	-	-	-	-	-	-	-	-	-	-	-	-
C4	1.44	74.33	-86.61	-	-	-	-	-	-	-	-	-	-	-	-	-	-	-	-	-	-	-
C5	-1.14	-43.18	35.21	-86.43	-	-	-	-	-	-	-	-	-	-	-	-	-	-	-	-	-	-
C6	2.74	72.66	-43.59	74.85	-84.67	-	-	-	-	-	-	-	-	-	-	-	-	-	-	-	-	-
O7	-2.24	-56.05	25.31	-33.11	25.44	-57.60	-	-	-	-	-	-	-	-	-	-	-	-	-	-	-	-
C8	-0.51	-16.32	8.13	-10.33	7.73	-15.03	28.07	-	-	-	-	-	-	-	-	-	-	-	-	-	-	-
H9	0.37	14.17	-6.99	8.38	-5.93	10.74	-15.79	-11.82	-	-	-	-	-	-	-	-	-	-	-	-	-	-
H10	0.34	11.78	-6.16	8.08	-6.00	11.41	-18.01	-13.11	6.62	-	-	-	-	-	-	-	-	-	-	-	-	-
H11	0.35	10.73	-5.82	7.60	-5.91	11.17	-14.68	-10.92	5.52	6.13	-	-	-	-	-	-	-	-	-	-	-	-
H12	1.00	22.52	-12.09	14.93	-12.08	22.82	-17.36	-5.74	4.35	3.67	4.63	-	-	-	-	-	-	-	-	-	-	-
F13	-0.89	-67.20	27.58	-25.64	15.46	-25.08	24.15	8.69	-8.92	-6.21	-5.59	-9.53	-	-	-	-	-	-	-	-	-	-
F14	-0.84	-63.94	25.79	-29.65	17.00	-29.12	23.63	6.71	-5.67	-5.29	-4.26	-6.97	20.08	-	-	-	-	-	-	-	-	-
H15	0.41	29.17	-40.25	29.97	-12.62	16.07	-10.26	-3.42	3.01	2.68	2.42	4.35	-11.92	-11.68	-	-	-	-	-	-	-	-
H16	0.47	27.27	-37.47	28.12	-14.33	17.38	-10.53	-3.63	3.17	2.71	2.70	5.82	-11.64	-8.46	11.25	-	-	-	-	-	-	-
F17	-0.50	-24.70	27.35	-68.71	27.29	-24.90	12.38	4.12	-3.38	-3.28	-3.10	-5.62	9.87	10.19	-11.78	-11.58	-	-	-	-	-	-
F18	-0.56	-29.53	26.51	-67.32	26.44	-29.72	14.42	4.45	-3.59	-3.60	-3.19	-5.51	10.56	14.78	-12.03	-8.70	20.44	-	-	-	-	-
H19	0.41	15.98	-12.67	30.01	-40.21	29.49	-10.36	-3.23	2.46	2.60	2.47	4.36	-6.09	-6.92	5.04	5.21	-11.79	-12.05	-	-	-	-
H20	0.47	17.22	-14.36	28.13	-37.40	27.54	-10.59	-3.45	2.67	2.65	2.76	5.82	-6.73	-6.46	5.21	6.85	-11.60	-8.71	11.27	-	-	-
F21	-0.88	-24.38	15.20	-25.15	27.01	-66.74	24.22	7.16	-4.87	-5.61	-5.83	-9.42	9.74	10.10	-5.96	-6.61	9.69	10.34	-11.70	-11.46	-	-
F22	-0.84	-28.78	16.96	-29.51	25.71	-64.48	23.99	6.21	-4.46	-5.08	-4.37	-6.98	10.29	14.48	-6.88	-6.44	10.17	14.68	-11.71	-8.46	19.66	-

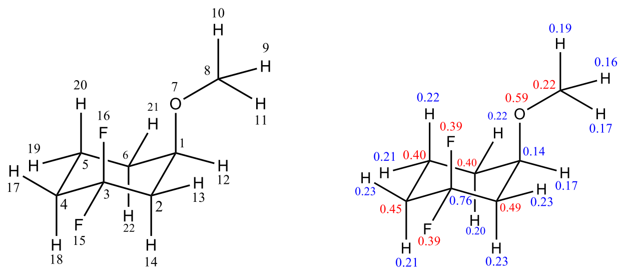

Table S17. Atom-atom electrostatic interactions (kcal mol^{-1}) obtained at M06-2X/aug-cc-pVTZ level using NPA charges for 14_{ax}.

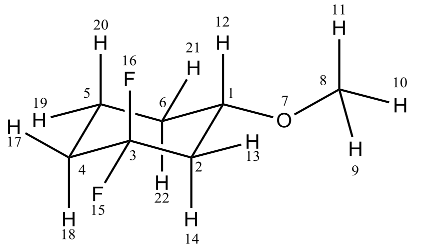

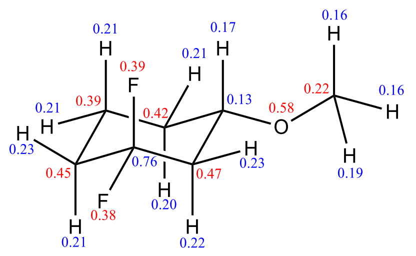

	C1	C2	C3	C4	C5	C6	O7	C8	H9	H10	H11	H12	H13	H14	F15	F16	H17	H18	H19	H20	H21	H22
C1	-	-	-	-	-	-	-	-	-	-	-	-	-	-	-	-	-	-	-	-	-	-
C2	-14.57	-	-	-	-	-	-	-	-	-	-	-	-	-	-	-	-	-	-	-	-	-
C3	13.75	-82.05	-	-	-	-	-	-	-	-	-	-	-	-	-	-	-	-	-	-	-	-
C4	-6.93	28.89	-75.59	-	-	-	-	-	-	-	-	-	-	-	-	-	-	-	-	-	-	-
C5	-7.23	22.05	-40.37	39.04	-	-	-	-	-	-	-	-	-	-	-	-	-	-	-	-	-	-
C6	-12.01	25.97	-34.78	23.85	34.73	-	-	-	-	-	-	-	-	-	-	-	-	-	-	-	-	-
O7	-19.01	38.81	-48.65	24.64	26.78	33.33	-	-	-	-	-	-	-	-	-	-	-	-	-	-	-	-
C8	-4.24	12.21	-15.22	7.21	6.95	8.04	30.71	-	-	-	-	-	-	-	-	-	-	-	-	-	-	-
H9	2.80	-8.16	9.46	-4.56	-4.33	-5.30	-14.92	-10.61	-	-	-	-	-	-	-	-	-	-	-	-	-	-
H10	2.67	-7.80	10.65	-5.35	-5.28	-5.85	-18.51	-12.90	5.65	-	-	-	-	-	-	-	-	-	-	-	-	-
H11	2.79	-1-	13.19	-5.75	-5.13	-5.52	-16.10	-11.50	5.04	6.13	-	-	-	-	-	-	-	-	-	-	-	-
H12	7.06	-12.89	12.45	-6.45	-6.48	-10.46	-16.19	-4.73	3.78	3.06	3.00	-	-	-	-	-	-	-	-	-	-	-
H13	4.72	-33.81	26.98	-9.81	-7.66	-8.69	-16.14	-6.25	4.34	3.86	5.75	5.04	-	-	-	-	-	-	-	-	-	-
H14	4.79	-33.54	26.98	-11.94	-8.86	-10.78	-13.03	-4.25	3.04	2.88	3.38	5.28	9.63	-	-	-	-	-	-	-	-	-
F15	-4.74	26.71	-71.26	24.52	13.79	12.39	17.39	6.00	-3.92	-4.36	-5.33	-4.86	-11.13	-11.40	-	-	-	-	-	-	-	-
F16	-5.85	26.72	-71.53	24.60	17.23	14.40	26.17	8.73	-5.00	-6.30	-8.21	-5.45	-11.43	-8.85	22.73	-	-	-	-	-	-	-
H17	2.61	-10.65	27.02	-31.01	-13.68	-8.65	-10.23	-3.15	1.98	2.43	2.56	2.57	4.02	4.45	-11.07	-11.47	-	-	-	-	-	-
H18	2.83	-12.08	25.18	-28.71	-12.80	-1-	-9.53	-2.90	1.91	2.16	2.29	2.84	4.16	5.87	-10.62	-8.25	8.90	-	-	-	-	-
H19	2.74	-8.59	15.32	-14.46	-25.26	-12.81	-10.50	-2.93	1.88	2.30	2.15	2.73	3.16	3.71	-5.93	-6.78	6.17	5.83	-	-	-	-
H20	3.64	-10.81	20.05	-15.30	-26.70	-13.64	-16.64	-4.23	2.50	3.33	3.10	3.29	4.04	4.11	-6.95	-10.42	6.63	5.00	8.65	-	-	-
H21	4.67	-10.32	14.13	-9.44	-13.35	-26.72	-16.86	-4.15	2.77	3.19	2.73	4.95	3.83	4.34	-5.39	-6.43	3.80	4.02	6.04	6.44	-	-
H22	4.19	-11.55	15.17	-10.62	-12.04	-23.97	-11.67	-3.22	2.24	2.37	2.28	4.55	3.91	5.67	-5.89	-5.95	3.89	5.26	5.44	4.71	8.11	-

Table S18. Atom-atom electrostatic interactions (kcal mol^{-1}) obtained at M06-2X/aug-cc-pVTZ level using NPA charges for 14_{eq}.

	C1	C2	C3	C4	C5	C6	O7	C8	H9	H10	H11	H12	H13	H14	F15	F16	H17	H18	H19	H20	H21	H22
C1	-	-	-	-	-	-	-	-	-	-	-	-	-	-	-	-	-	-	-	-	-	-
C2	-13.72	-	-	-	-	-	-	-	-	-	-	-	-	-	-	-	-	-	-	-	-	-
C3	13.65	-78.10	-	-	-	-	-	-	-	-	-	-	-	-	-	-	-	-	-	-	-	-
C4	-6.82	27.52	-75.50	-	-	-	-	-	-	-	-	-	-	-	-	-	-	-	-	-	-	-
C5	-7.02	20.74	-40.03	38.60	-	-	-	-	-	-	-	-	-	-	-	-	-	-	-	-	-	-
C6	-12.32	25.87	-36.48	24.90	35.92	-	-	-	-	-	-	-	-	-	-	-	-	-	-	-	-	-
O7	-18.43	38.29	-39.70	20.50	20.08	33.01	-	-	-	-	-	-	-	-	-	-	-	-	-	-	-	-
C8	-4.13	9.26	-11.36	6.24	6.46	10.06	29.88	-	-	-	-	-	-	-	-	-	-	-	-	-	-	-
H9	2.61	-6.81	8.49	-4.63	-4.58	-6.63	-18.24	-12.70	-	-	-	-	-	-	-	-	-	-	-	-	-	-
H10	2.63	-6.02	7.83	-4.58	-4.97	-7.99	-14.92	-10.67	5.76	-	-	-	-	-	-	-	-	-	-	-	-	-
H11	2.80	-6.43	8.29	-4.49	-4.68	-6.73	-15.14	-10.75	5.79	4.89	-	-	-	-	-	-	-	-	-	-	-	-
H12	6.82	-12.05	15.21	-7.47	-7.90	-10.84	-15.70	-4.73	3.02	2.95	3.93	-	-	-	-	-	-	-	-	-	-	-
H13	4.81	-32.97	27.56	-10.05	-7.76	-9.35	-17.09	-4.34	3.34	2.70	3.12	5.24	-	-	-	-	-	-	-	-	-	-
H14	4.65	-31.51	26.48	-11.82	-8.67	-11.14	-16.75	-4.09	3.13	2.74	2.69	4.05	9.68	-	-	-	-	-	-	-	-	-
F15	-4.60	24.98	-70.30	24.11	13.44	12.72	15.58	4.60	-3.60	-3.18	-3.34	-5.23	-11.21	-10.87	-	-	-	-	-	-	-	-
F16	-6.00	25.88	-72.27	25.04	17.62	15.61	18.06	5.54	-4.14	-3.78	-4.29	-8.20	-11.84	-8.85	22.67	-	-	-	-	-	-	-
H17	2.57	-10.13	26.96	-31.02	-13.54	-9.05	-8.27	-2.63	2.00	1.94	1.94	3.03	4.11	4.40	-10.95	-11.59	-	-	-	-	-	-
H18	2.83	-11.75	25.53	-29.21	-12.87	-10.59	-9.28	-2.80	2.13	2.08	1.95	2.94	4.34	5.94	-10.49	-8.51	9.05	-	-	-	-	-
H19	2.74	-8.33	15.59	-14.70	-25.42	-13.64	-8.88	-3.00	2.19	2.40	2.16	3.13	3.30	3.74	-5.93	-7.08	6.27	6.02	-	-	-	-
H20	3.33	-9.58	18.83	-14.36	-24.70	-13.33	-9.68	-3.27	2.32	2.47	2.52	4.44	3.86	3.80	-6.45	-10.17	6.25	4.77	8.27	-	-	-
H21	4.35	-9.38	13.57	-9.05	-12.71	-26.79	-14.71	-5.47	3.50	4.97	3.73	4.71	3.76	4.10	-5.08	-6.38	3.65	3.91	5.92	5.79	-	-
H22	4.22	-11.27	15.47	-10.78	-12.14	-25.60	-14.62	-4.34	3.06	3.53	2.73	3.67	4.11	5.75	-5.87	-6.24	3.96	5.41	5.65	4.49	7.94	-

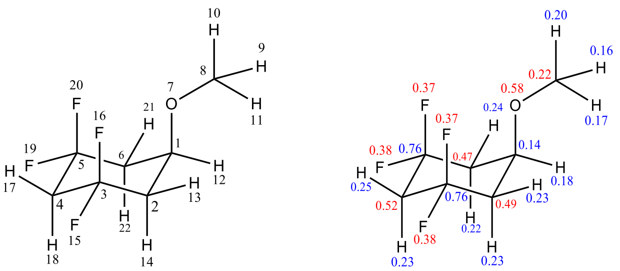

Table S19. Atom-atom electrostatic interactions (kcal mol^{-1}) obtained at M06-2X/aug-cc-pVTZ level using NPA charges for $\mathbf{15_{ax}}$.

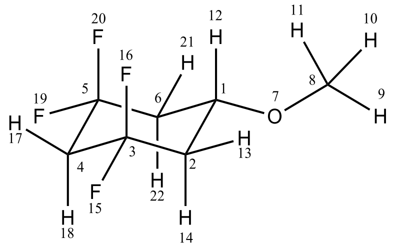

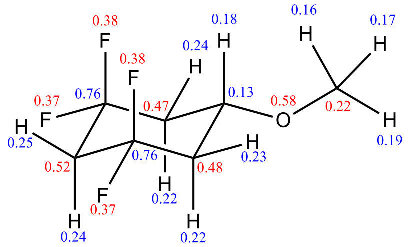

	C1	C2	C3	C4	C5	C6	O7	C8	H9	H10	H11	H12	H13	H14	F15	F16	H17	H18	F19	F20	H21	H22
C1	-	-	-	-	-	-	-	-	-	-	-	-	-	-	-	-	-	-	-	-	-	-
C2	-14.63 / -13.97	-	-	-	-	-	-	-	-	-	-	-	-	-	-	-	-	-	-	-	-	-
C3	13.53 / 13.57	-80.78 / -81.88	-	-	-	-	-	-	-	-	-	-	-	-	-	-	-	-	-	-	-	-
C4	-7.84 / -7.54	32.64 / 31.04	-86.40 / -86.48	-	-	-	-	-	-	-	-	-	-	-	-	-	-	-	-	-	-	-
C5	13.54 / 13.58	-41.78 / -42.18	76.69 / 76.82	-86.47 / -86.28	-	-	-	-	-	-	-	-	-	-	-	-	-	-	-	-	-	-
C6	-13.89 / -13.19	29.70 / 39.76	-40.25 / -40.24	31.45 / 31.55	-78.84 / -73.14	-	-	-	-	-	-	-	-	-	-	-	-	-	-	-	-	-
O7	-18.69 / -17.49	37.93 / 37.95	-48.87 / -38.51	23.29 / 27.53	-40.68 / -39.53	37.96 / 37.85	-	-	-	-	-	-	-	-	-	-	-	-	-	-	-	-
C8	-4.04 / -4.01	12.65 / 11.26	-15.69 / -12.62	7.20 / 8.29	-13.41 / -11.44	9.43 / 9.31	30.28 / 29.79	-	-	-	-	-	-	-	-	-	-	-	-	-	-	-
H9	2.79 / 2.56	-8.17 / -7.78	9.47 / 9.03	-5.23 / -5.40	8.21 / 8.63	-6.20 / -6.90	-14.65 / -18.98	-19.71 / -19.01	-	-	-	-	-	-	-	-	-	-	-	-	-	-
H10	2.71 / 2.77	-7.98 / -7.86	10.89 / 9.25	-6.27 / -5.28	10.11 / 8.51	-6.98 / -6.59	-18.60 / -15.40	-13.32 / -11.14	5.81 / 6.04	-	-	-	-	-	-	-	-	-	-	-	-	-
H11	2.76 / 2.56	-9.98 / -9.30	13.17 / 9.76	-6.59 / -5.29	9.62 / 7.89	-6.44 / -6.05	-15.80 / -14.91	-11.61 / -10.83	5.06 / 5.90	6.29 / 5.03	-	-	-	-	-	-	-	-	-	-	-	-
H12	7.20 / 7.19	-13.30 / -13.59	12.84 / 16.68	-7.64 / -9.44	12.82 / 16.67	-13.68 / -13.11	-16.38 / -16.98	-4.92 / -5.22	3.91 / 3.35	3.24 / 4.40	3.09 / 3.28	-	-	-	-	-	-	-	-	-	-	-
H13	4.82 / 4.58	-34.82 / -34.12	27.82 / 27.76	-11.63 / -11.53	15.07 / 14.98	-10.45 / -10.32	-16.35 / -16.00	-6.51 / -5.99	4.50 / 3.91	4.09 / 4.12	5.95 / 5.49	5.38 / 5.64	-	-	-	-	-	-	-	-	-	-
H14	4.76 / 4.50	-33.69 / -32.89	27.11 / 26.62	-13.87 / -13.59	17.34 / 17.10	-12.64 / -12.53	-12.85 / -16.14	-4.31 / -4.83	3.07 / 3.45	3.08 / 3.07	3.40 / 3.92	5.49 / 4.45	1 / 9.72	-	-	-	-	-	-	-	-	-
F15	-4.60 / -4.37	26.19 / 25.52	-70.03 / -69.42	27.86 / 27.46	-26.02 / -25.70	14.15 / 13.93	16.73 / 14.97	5.94 / 5.16	-3.86 / -3.84	-4.38 / -3.74	-5.24 / -4.16	-4.93 / -5.62	-11.27 / -11.08	-11.27 / -10.83	-	-	-	-	-	-	-	-
F16	-5.58 / -5.54	25.65 / 25.83	-69.09 / -69.88	27.11 / 27.54	-31.01 / -31.80	16.11 / 16.41	24.7 / 17.01	8.43 / 5.94	-4.81 / -4.25	-6.19 / -4.68	-7.86 / -4.53	-5.41 / -8.60	-11.33 / -11.51	-8.58 / -8.58	21.50 / 21.51	-	-	-	-	-	-	-
H17	2.85 / 2.74	-11.62 / -11.46	29.25 / 29.22	-39.14 / -39.05	29.30 / 29.18	-11.10 / -11.04	-11.03 / -9.02	-3.48 / -2.92	2.18 / 2.23	2.73 / 2.20	2.81 / 2.16	2.91 / 3.70	4.53 / 4.49	4.89 / 4.80	-11.72 / -11.58	-12.07 / -12.24	-	-	-	-	-	-
H18	3.09 / 2.99	-13.41 / -13.34	28.01 / 28.25	-37.01 / -37.38	27.98 / 28.21	-12.71 / -12.84	-10.34 / -10.07	-3.24 / -3.10	2.13 / 2.37	2.46 / 2.20	2.55 / 2.30	3.25 / 3.56	4.79 / 4.78	6.59 / 6.51	-11.81 / -11.68	-8.88 / -9.09	10.88 / 10.97	-	-	-	-	-
F19	-4.63 / -4.36	14.93 / 14.43	-26.14 / -25.67	28.00 / 27.40	-70.37 / -69.21	25.21 / 24.53	17.14 / 15.20	5.02 / 4.56	-3.26 / -3.59	-4.02 / -3.37	-3.69 / -3.15	-4.95 / -5.61	-5.70 / -5.54	-6.71 / -6.45	10.30 / 9.95	10.95 / 11.02	-11.74 / -11.55	-11.91 / -11.66	-	-	-	-
F20	-5.41 / -5.53	16.39 / 17.01	-30.31 / -31.82	26.72 / 27.54	-68.19 / -69.79	24.01 / 24.84	24.52 / 17.21	6.60 / 5.37	-3.92 / -4.05	-5.37 / -4.25	-4.81 / -3.66	-5.26 / -8.59	-6.43 / -6.71	-6.42 / -6.61	10.71 / 11.04	15.32 / 16.60	-11.98 / -12.25	-8.74 / -9.09	21.23 / 21.45	-	-	-
H21	5.08 / 4.78	-11.38 / -11.10	15.70 / 15.49	-12.06 / -11.88	28.92 / 28.44	-34.60 / -33.94	-18.40 / -17.49	-4.64 / -4.52	3.07 / 3.50	3.64 / 3.32	3.04 / 2.81	5.57 / 5.87	4.37 / 4.27	4.82 / 4.73	-5.88 / -5.73	-6.92 / -6.94	4.71 / 4.63	4.92 / 4.93	-11.62 / -11.32	-11.66 / -11.80	-	-
H22	4.65 / 4.51	-12.83 / -13.01	16.75 / 17.12	-13.40 / -13.59	26.59 / 26.59	-31.59 / -31.66	-12.89 / -16.63	-3.66 / -4.12	2.54 / 3.19	2.74 / 2.77	2.57 / 2.77	5.36 / 4.45	4.52 / 4.57	6.35 / 6.49	-6.43 / -6.46	-6.39 / -6.61	4.76 / 4.80	6.32 / 6.51	-11.25 / -10.82	-8.26 / -8.57	10.10 / 10.05	-

Table S20. Atom-atom electrostatic interactions (kcal mol^{-1}) obtained at M06-2X/aug-cc-pVTZ level using NPA charges for 15_{eq}.

Table S21. Popelier criterea parameters from QTAIM calculations run at the M06-2X/aug-cc-pVTZ wavefunction density for the molecules with axial hydrogens in positions 3 and/or 5 for compounds 1-15.

Compound	Anomer	Hydrogen	*q*(A)	*E*(A)	*μ1*(A)	*V*(A)
**1**	**ax**	1	0.006	-0.001	-0.006	-1.564
		2	-0.002	-0.001	0.000	0.061
	**eq**	1	-0.022	-0.009	0.001	1.223
		2	-0.013	-0.004	-0.005	-0.865
**8**	**ax**	1	0.023	0.007	-0.006	-2.413
		2	0.006	0.002	0.001	-0.515
	**eq**	1	-0.003	-0.002	-0.001	-0.123
		2	-0.024	-0.006	0.010	2.609
**11**	**ax**	1	0.026	0.007	-0.008	-2.769
		2	0.015	0.003	-0.008	-2.021
	**eq**	1	-0.002	-0.002	-0.001	-0.028
		2	-0.003	-0.002	-0.001	-0.028
**12**	**ax**	1	0.019	0.006	-0.005	-1.958
		2	0.005	0.002	0.001	-0.525
	**eq**	1	-0.003	-0.002	-0.001	-0.064
		2	-0.023	-0.007	0.008	2.293
**13**	**ax**	1	0.003	0.002	0.001	-0.209
		2	0.002	0.001	0.002	-0.175
	**eq**	1	-0.024	-0.008	0.008	2.153
		2	-0.024	-0.008	0.007	2.123
**14**	**ax**	1	-	-	-	-
		2	0.026	0.007	-0.007	-2.648
	**eq**	1	-	-	-	-
		2	-0.002	-0.002	-0.001	-0.084

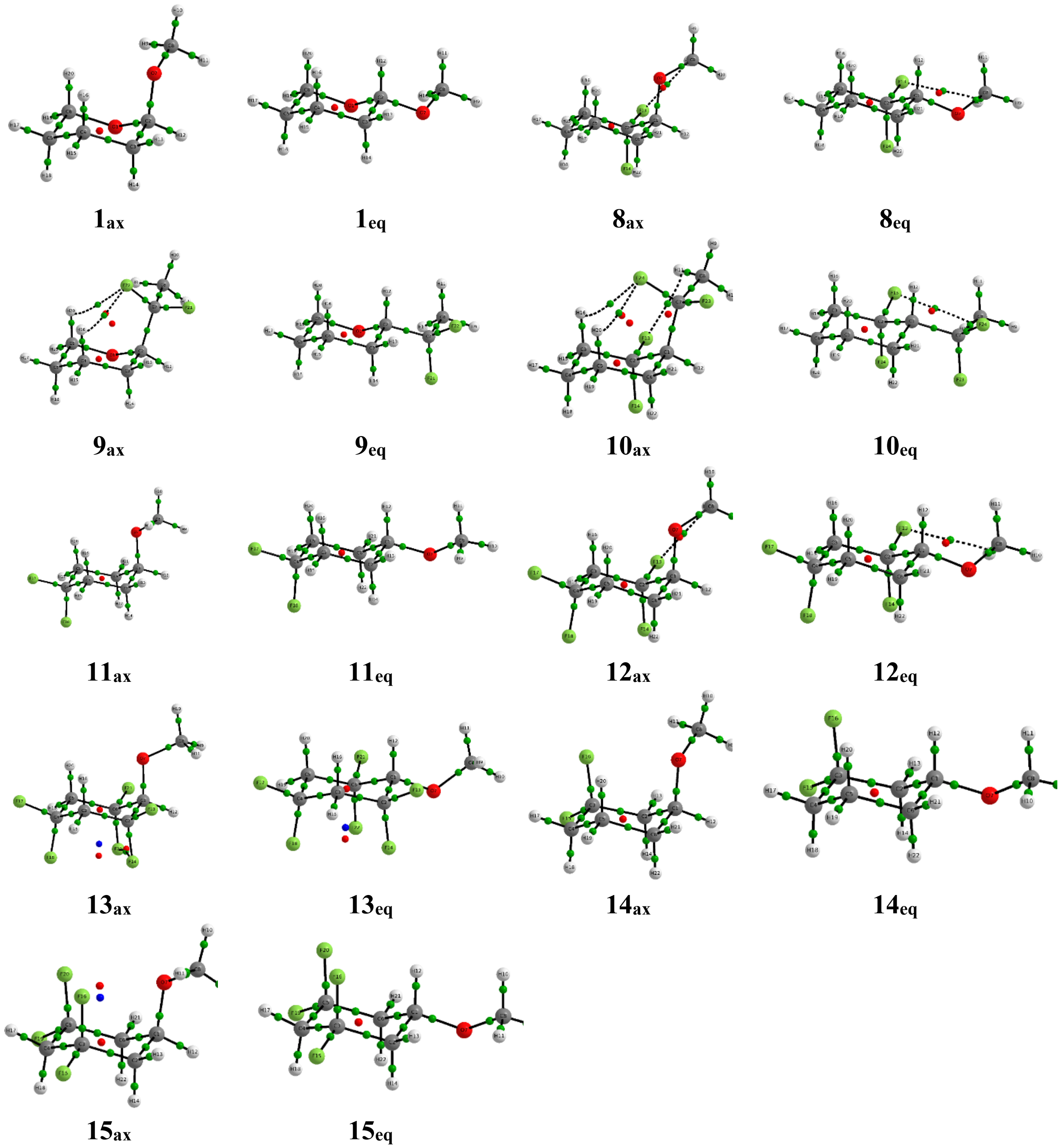

Figure S1. QTAIM molecular graphs for molecules 1-15. bond critical points (green spheres), ring critical points (red spheres) and cage critical points (blue shperes).

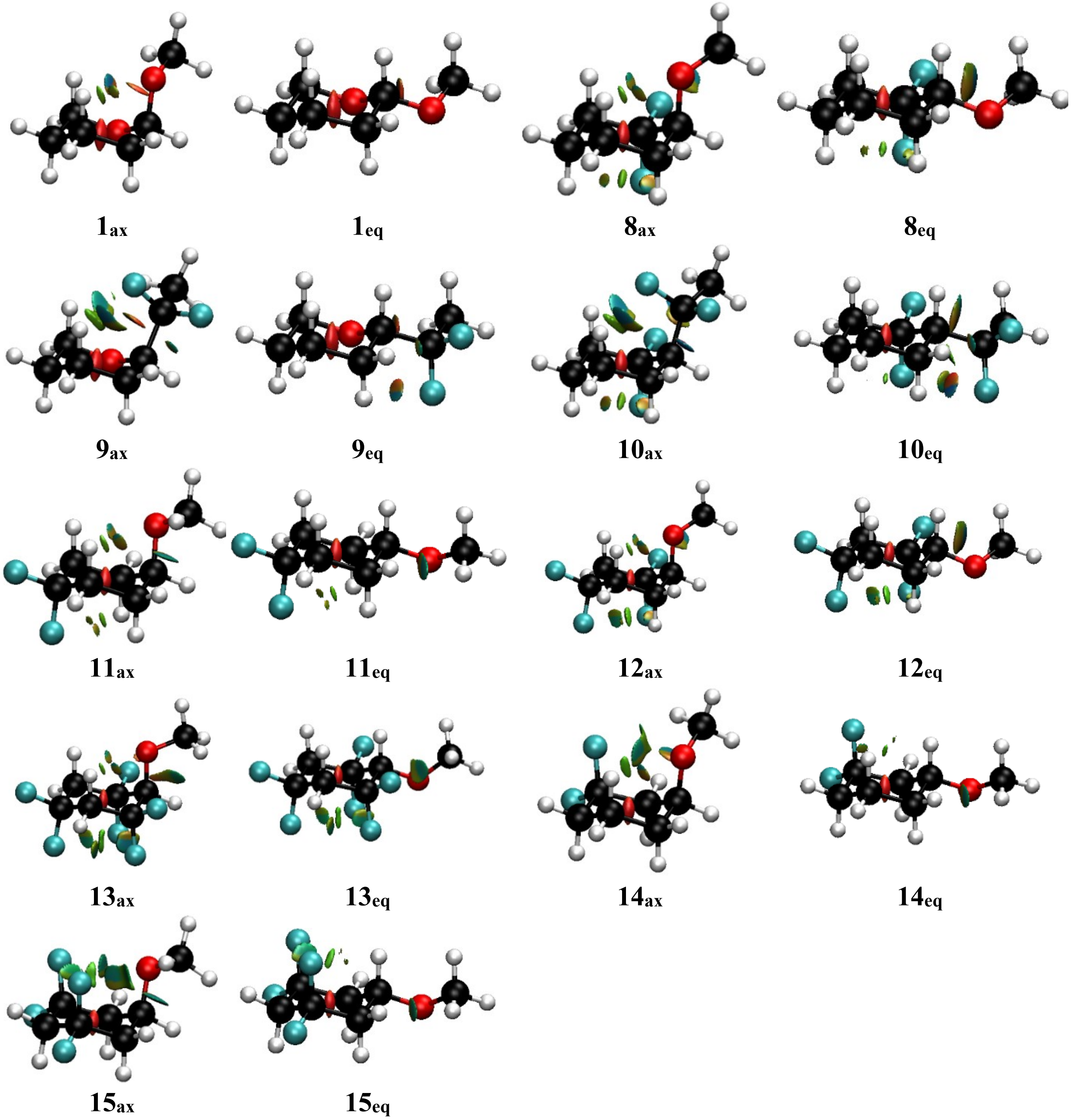

Figure S2. Isosurfaces from NCI for molecules 1-15, using reduced density gradient (RDG) = 0.5 and blue-green-red color scale ranging from $-0.02 < \mathrm{sign}(\lambda_2)\rho(\mathrm{r}) < +0.02$ au.

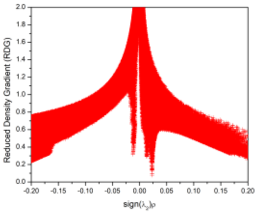
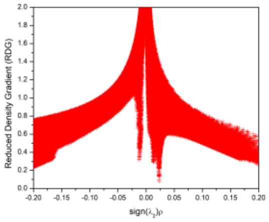
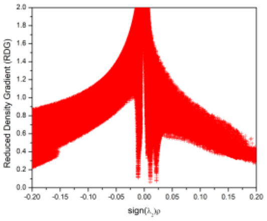
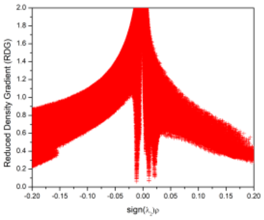

1ax **1eq** **8ax** **8eq**

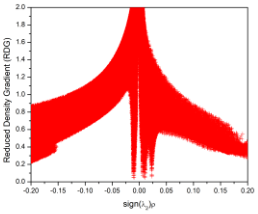
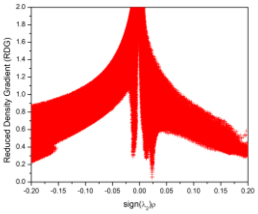
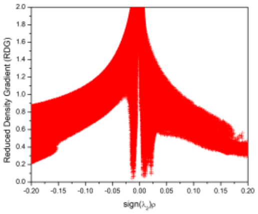
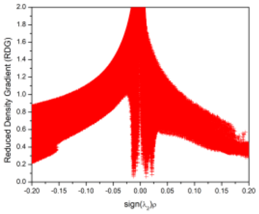

9ax **9eq** **10ax** **10eq**

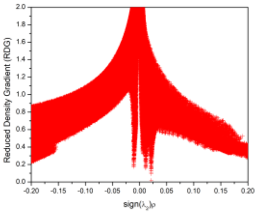
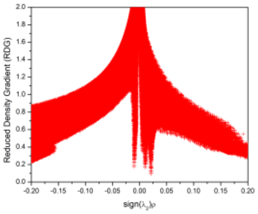
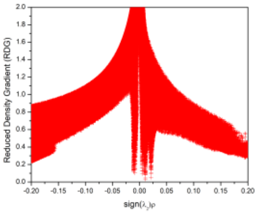
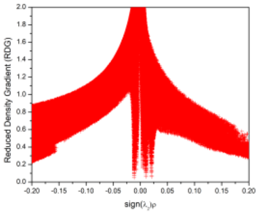

11ax **11eq** **12ax** **12eq**

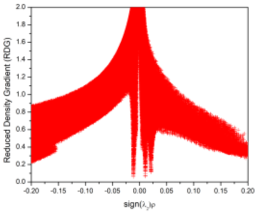
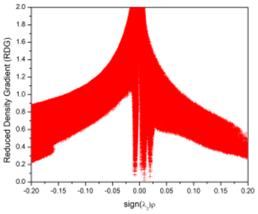
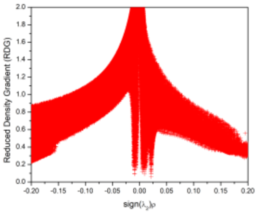
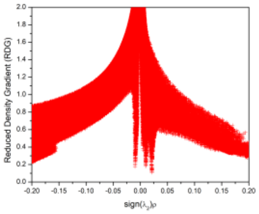

13ax **13eq** **14ax** **14eq**

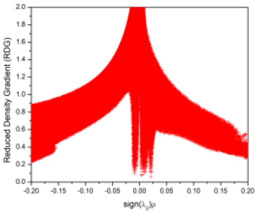
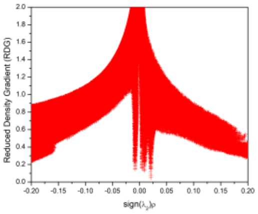

15ax **15eq**

Figure S3: Reduced RDG versus sign(λ_2)ρ plots for compounds 1-15.

Table S22. Calculated total relative energy *ΔE* and relative Gibbs energy values in kcal mol^{-1} and ax/eq population percentages obtained at DLPNO-CCSD(T) and M06-2X/aug-cc-pVTZ[levels for compounds 1-15. Relative Gibbs free energies (kcal mol^{-1}) and population percentages are also shown in different solvents using the polarizable continuum model at the M06-2X/aug-cc-pVTZ level. Dipole moments (*μ*) calculated in each medium at the M06-2X/aug-cc-pVTZ level are also given in Debyes. Negative energy values represent axial preference, and the positive ones equatorial preference.

	1	8	9	10	11	12	13	14	15
***ΔE* [DLPNO-CCSD(T)]**	-1.23	-1.54	2.37	1.02	-1.09	-2.42	-3.61	1.71	2.59
**M06-2X**									
ΔE	-1.39	-1.36	1.95	1.06	-1.08	-2.28	-3.81	0.80	2.53
**%*P*(Δ*E*) ax/eq[a]**	97.4/ 2.6	97.2/ 2.8	0.6/ 99.4	5.9/ 94.1	94.3/ 5.7	99.7/ 0.3	100.0/ 0.0	11.1/ 88.9	0.1/ 99.9
ΔG	-1.11	-1.34	2.26	0.93	-0.79	-1.96	-3.32	1.55	3.13
**%*P*(Δ*G*) ax/eq[a]**	94.7/ 5.3	97.0/ 3.0	0.3/ 99.7	8.1/ 91.9	88.7/ 11.3	99.4/ 0.6	100.0/ 0.0	1.7/ 98.3	0.0/ 100.0
***μ* (gas phase) ax/eq**	0.42/1.83	1.57/2.62	1.26/2.70	0.60/2.82	3.14/2.85	3.21/3.15	3.24/4.05	2.47/3.09	3.78/2.54
***ΔG*($CHCl_3$)**	-0.61	-0.49	2.55	1.56	-0.37	-1.06	-1.94	1.25	1.94
**%*P*(ΔG_{CHCl3}) ax/eq[a]**	83.1/ 16.9	78.2/ 21.8	0.1/ 99.9	1.7/ 98.3	72.4/ 27.6	99.4/ 0.6	100.0/ 0.0	1.7/ 98.3	0.0/ 100.0
***μ* ($CHCl_3$) ax/eq**	0.52/2.36	1.86/3.38	1.51/3.29	0.73/3.45	3.72/3.43	3.81/4.10	3.84/5.18	3.10/3.72	4.75/2.98

***ΔG*(CH_2Cl_2)**	-0.47	-0.11	2.66	1.77	-0.27	-0.66	-1.66	1.14	1.59
**%*P*(Δ*G*$_{CH2Cl2}$) ax/eq[a]**	77.3/ 22.7	57.1/ 42.9	0.1/ 99.9	1.0/ 99.0	66.9/ 33.1	84.8/ 15.2	98.7/ 1.3	4.9/ 95.1	1.6/ 98.4
***μ* (CH_2Cl_2) ax/eq**	0.55/2.50	1.92/3.85	1.56/3.43	0.76/3.60	3.83/3.56	3.93/4.39	3.97/5.49	3.26/3.86	5.00/3.07
***ΔG*(acetone)**	-0.36	-0.09	2.76	1.91	-0.19	-0.64	-1.30	1.11	1.41
**%*P*(Δ*G* acetone) ax/eq[a]**	71.9/ 28.1	55.8/ 44.2	0.1/ 99.9	0.7/ 99.3	62.1/ 37.9	84.1/ 15.9	96.7/ 3.3	5.3/ 94.7	2.5/ 97.5
***μ* (acetone) ax/eq**	0.56/2.61	1.96/4.02	1.59/3.53	0.77/3.72	3.92/3.67	4.02/4.85	4.06/5.73	3.38/3.96	5.19/3.14

[a] Populations were measured using a Boltzmann Constant of 0.001987 kcal mol^{-1} and Temperature of 193.15 K

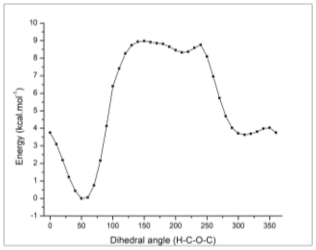

$\mathbf{1}_{\mathbf{ax}}$

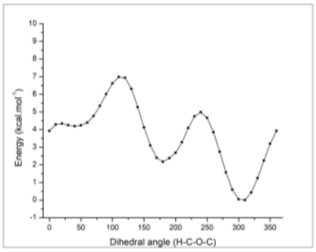

$\mathbf{1}_{\mathbf{eq}}$

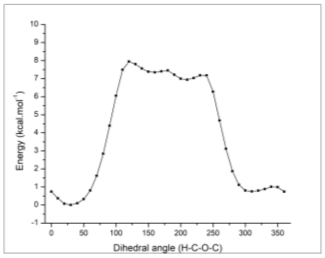

$\mathbf{8}\ _{\mathbf{ax}}$

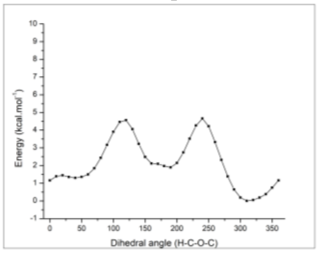

$\mathbf{8}_{\mathbf{eq}}$

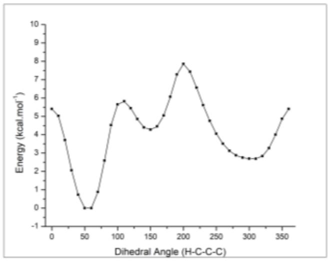

$\mathbf{9}_{\mathbf{ax}}$

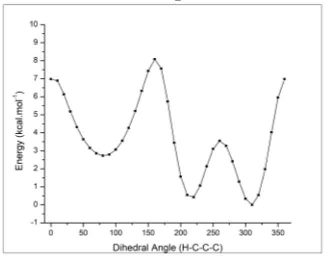

$\mathbf{9}_{\mathbf{eq}}$

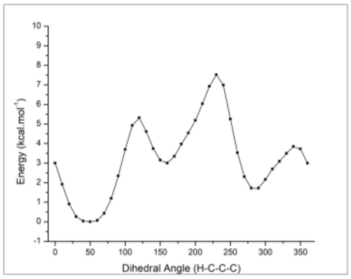

$\mathbf{10}_{\mathbf{ax}}$

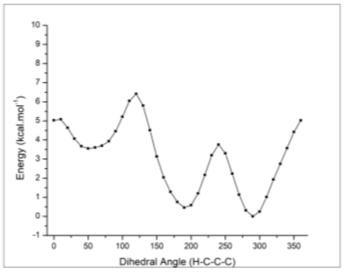

$\mathbf{10}_{\mathbf{eq}}$

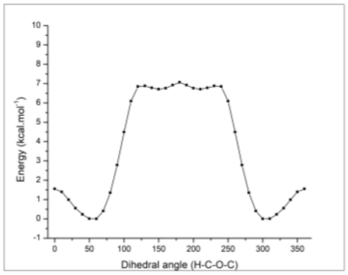

$\mathbf{11}_{\mathbf{ax}}$

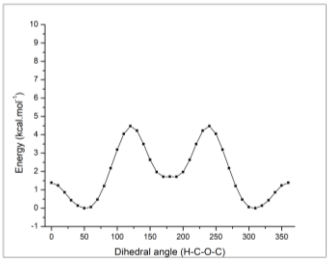

$\mathbf{11}_{\mathbf{eq}}$

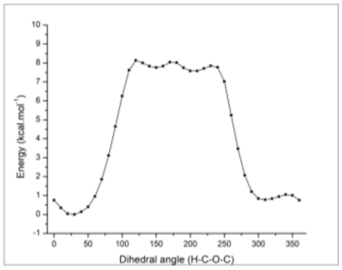

12_{ax}

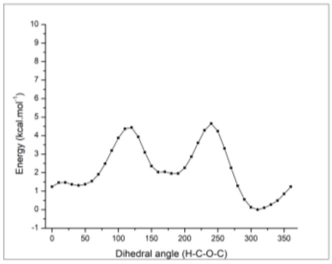

12_{eq}

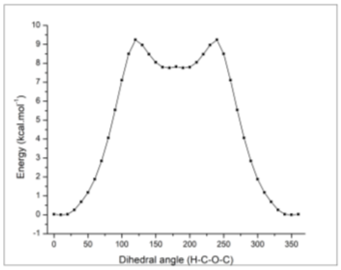

13_{ax}

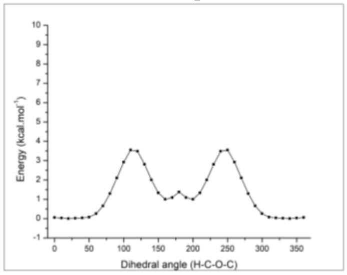

13_{eq}

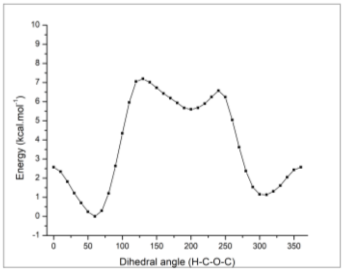

14_{ax}

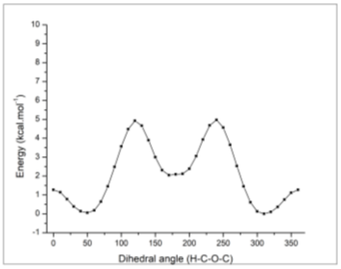

14_{eq}

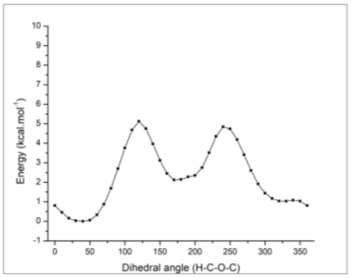

15_{ax}

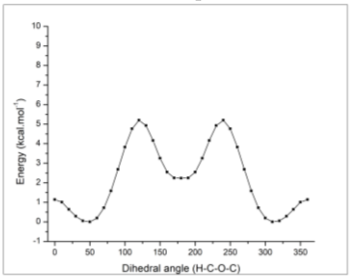

15_{eq}

Figure S4. Potential energy curves (kcal mol^{-1}) for the rotation of the H-C-O-C dihedral angle from 0-360° in steps of 10° for molecules 1-15 calculated at the M06-2X/aug-cc-pVTZ level.

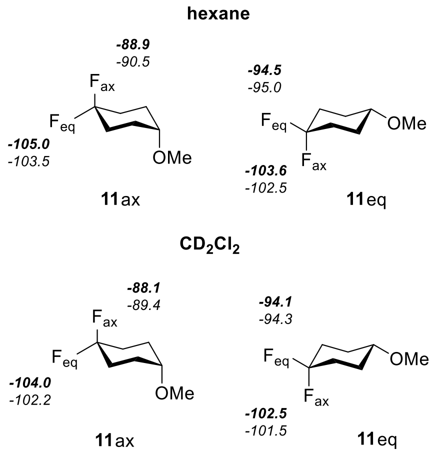

Figure S5. Calculated (in italics) and experimental (in bold) ^{19}F-NMR chemical shits in ppm for the anomers 8_{ax} and 8_{eq} and 11_{ax} and 11_{eq}. The calculated values were obtained at the mPW1PW91/6-31G(d) level by the GIAO method and corrected using a scaling factor of 0.84.

Table S23: Atomic charges obtained from QTAIM calculations from M06-2X/aug-cc-pVTZ wavefunctions for molecules 1-15.

Atomic Number	1_{ax}	1_{eq}	8_{ax}	8_{eq}	9_{ax}	9_{eq}	10_{ax}	10_{eq}	11_{ax}	11_{eq}	12_{ax}	12_{eq}	13_{ax}	13_{eq}	14_{ax}	14_{eq}	15_{ax}	15_{eq}
**1**	-1.235	-1.221	0.603	0.626	-1.226	-1.223	0.075	0.077	0.573	0.583	0.603	0.632	0.640	0.677	0.593	0.583	0.605	0.590
**2**	1.146	1.156	1.243	1.250	0.618	0.613	1.241	1.245	0.053	0.056	1.263	1.269	1.286	1.291	0.048	0.062	0.047	0.054
**3**	0.053	0.052	0.044	0.045	0.058	0.058	0.033	0.037	0.045	0.047	0.047	0.045	0.045	0.046	1.239	1.232	1.258	1.255
**4**	0.057	0.052	0.050	0.047	0.046	0.053	0.054	0.048	1.234	1.240	1.254	1.260	1.272	1.278	0.049	0.045	0.049	0.046
**5**	0.041	0.038	0.049	0.043	0.045	0.036	0.037	0.042	0.050	0.044	0.046	0.041	0.047	0.045	0.050	0.048	1.268	1.251
**6**	0.575	0.584	0.061	0.059	0.573	0.585	0.075	0.073	0.061	0.060	0.058	0.055	1.291	1.302	0.059	0.056	0.056	0.058
**7**	-1.256	-1.264	-1.248	-1.245	1.246	1.257	1.244	1.257	-1.243	-1.239	-1.247	-1.242	-1.245	-1.239	-1.238	-1.237	-1.229	-1.232
**8**	0.588	0.588	0.588	0.594	0.021	0.026	0.015	0.017	0.602	0.604	0.580	0.586	0.567	0.576	0.597	0.602	0.591	0.591
**9**	0.027	0.022	0.023	0.020	0.036	0.034	0.038	0.036	0.009	0.008	0.011	0.038	0.026	0.037	0.002	0.026	0.002	0.031
**10**	0.022	0.039	0.007	0.036	0.035	0.049	0.022	0.046	0.026	0.026	0.028	0.026	0.037	0.039	0.025	0.007	0.032	0.016
**11**	0.010	0.005	0.040	0.006	0.024	0.015	0.048	0.023	0.006	0.006	0.042	0.009	0.040	0.014	0.023	0.009	0.023	0.010
**12**	0.014	-0.009	0.017	0.000	0.024	0.001	0.025	0.018	-0.011	-0.026	0.026	0.002	0.059	0.026	-0.011	-0.005	-0.002	0.015
**13**	0.004	0.002	-0.735	-0.734	0.009	0.006	-0.733	-0.730	-0.017	-0.016	-0.729	-0.728	-0.722	-0.721	0.018	0.034	0.027	0.025
**14**	-0.015	0.006	-0.733	-0.733	-0.015	0.005	-0.724	-0.727	0.001	0.003	-0.726	-0.727	-0.720	-0.719	0.010	0.015	0.011	0.015
**15**	-0.015	-0.009	0.021	0.027	-0.013	-0.007	0.024	0.029	0.023	0.029	0.062	0.068	0.069	0.075	-0.729	-0.729	-0.723	-0.724
**16**	-0.008	-0.029	0.021	0.001	-0.002	-0.029	0.024	0.004	0.014	0.002	0.053	0.036	0.059	0.037	-0.731	-0.729	-0.724	-0.723
**17**	-0.008	-0.008	-0.009	-0.007	-0.007	-0.006	-0.007	-0.006	-0.731	-0.731	-0.725	-0.726	-0.720	-0.720	0.025	0.027	0.067	0.068
**18**	-0.014	-0.007	-0.013	-0.006	-0.013	-0.005	-0.011	-0.007	-0.731	-0.732	-0.724	-0.725	-0.719	-0.719	-0.002	0.004	0.034	0.039
**19**	0.011	0.016	-0.013	-0.006	0.012	0.016	-0.010	-0.005	0.020	0.028	0.029	0.037	0.069	0.075	-0.012	-0.005	-0.722	-0.724
**20**	0.003	-0.012	-0.006	-0.030	0.004	-0.012	-0.002	-0.030	0.024	0.001	0.030	0.004	0.060	0.037	0.013	-0.008	-0.722	-0.722
**21**	-	-	-0.002	0.002	-0.735	-0.735	0.010	0.008	0.003	0.004	0.008	0.014	-0.721	-0.718	0.005	-0.017	0.048	0.043
**22**	-	-	-0.010	0.004	-0.742	-0.734	-0.010	0.004	-0.011	0.005	0.013	0.027	-0.719	-0.718	-0.033	-0.019	0.004	0.017
**23**	-	-	-	-	-	-	-0.729	-0.731	-	-	-	-	-	-	-	-	-	-
**24**	-	-	-	-	-	-	-0.738	-0.727	-	-	-	-	-	-	-	-	-	-

Table S24: Atomic charges obtained from the Natural Population Analysis (NPA) at the M06-2X/aug-cc-pVTZ level for molecules 1-15.

Atomic Number	1_{ax}	1_{eq}	8_{ax}	8_{eq}	9_{ax}	9_{eq}	10_{ax}	10_{eq}	11_{ax}	11_{eq}	12_{ax}	12_{eq}	13_{ax}	13_{eq}	14_{ax}	14_{eq}	15_{ax}	15_{eq}
1	-0.593	-0.595	0.072	0.079	-0.581	-0.583	-0.340	-0.336	0.137	0.139	0.073	0.081	0.010	0.017	0.137	0.135	0.136	0.130
2	0.430	0.442	0.744	0.737	0.073	0.078	0.776	0.772	-0.426	-0.424	0.744	0.737	0.745	0.739	-0.490	-0.466	-0.489	-0.483
3	-0.425	-0.426	-0.458	-0.453	-0.423	-0.426	-0.459	-0.448	-0.458	-0.451	-0.525	-0.520	-0.524	-0.520	0.763	0.762	0.762	0.762
4	-0.392	-0.391	-0.391	-0.393	-0.393	-0.386	-0.391	-0.393	0.763	0.762	0.763	0.762	0.762	0.761	-0.451	-0.451	-0.518	-0.517
5	-0.417	-0.419	-0.394	-0.390	-0.418	-0.420	-0.396	-0.385	-0.456	-0.452	-0.457	-0.453	-0.524	-0.518	-0.398	-0.394	0.763	0.761
6	-0.044	-0.038	-0.406	-0.405	-0.040	-0.030	-0.394	-0.398	-0.405	-0.406	-0.412	-0.412	0.748	0.746	-0.401	-0.420	-0.468	-0.465
7	-0.596	-0.570	-0.591	-0.572	0.738	0.746	0.770	0.772	-0.597	-0.579	-0.593	-0.568	-0.580	-0.553	-0.588	-0.581	-0.576	-0.576
8	-0.223	-0.223	-0.217	-0.216	-0.653	-0.653	-0.652	-0.652	-0.219	-0.219	-0.217	-0.218	-0.215	-0.217	-0.221	-0.218	-0.223	-0.220
9	0.174	0.186	0.187	0.184	0.229	0.225	0.226	0.226	0.163	0.162	0.164	0.180	0.173	0.179	0.158	0.191	0.159	0.194
10	0.186	0.181	0.162	0.179	0.225	0.237	0.221	0.234	0.191	0.191	0.191	0.187	0.195	0.198	0.191	0.161	0.196	0.167
11	0.164	0.160	0.181	0.161	0.222	0.217	0.236	0.222	0.160	0.160	0.182	0.163	0.180	0.166	0.171	0.163	0.171	0.162
12	0.156	0.119	0.188	0.171	0.202	0.170	0.237	0.231	0.172	0.153	0.195	0.172	0.214	0.195	0.170	0.167	0.175	0.183
13	0.215	0.213	-0.384	-0.387	0.223	0.221	-0.385	-0.387	0.210	0.210	-0.377	-0.381	-0.370	-0.374	0.227	0.232	0.234	0.232
14	0.203	0.211	-0.389	-0.381	0.206	0.215	-0.390	-0.385	0.218	0.215	-0.374	-0.366	-0.359	-0.352	0.225	0.222	0.227	0.224
15	0.204	0.208	0.223	0.227	0.205	0.209	0.225	0.227	0.225	0.228	0.245	0.248	0.251	0.254	-0.386	-0.379	-0.379	-0.374
16	0.204	0.191	0.224	0.212	0.209	0.191	0.227	0.213	0.221	0.213	0.247	0.236	0.250	0.237	-0.387	-0.393	-0.372	-0.377
17	0.206	0.206	0.209	0.209	0.208	0.208	0.210	0.210	-0.382	-0.382	-0.376	-0.376	-0.371	-0.371	0.226	0.226	0.248	0.248
18	0.196	0.201	0.201	0.206	0.197	0.202	0.203	0.206	-0.396	-0.391	-0.381	-0.376	-0.366	-0.361	0.210	0.213	0.235	0.237
19	0.187	0.189	0.207	0.211	0.188	0.190	0.209	0.212	0.224	0.228	0.230	0.234	0.251	0.254	0.208	0.212	-0.380	-0.373
20	0.165	0.154	0.206	0.191	0.165	0.154	0.210	0.191	0.227	0.212	0.230	0.214	0.251	0.237	0.220	0.206	-0.365	-0.377
21	-	-	0.214	0.216	-0.387	-0.380	0.227	0.225	0.217	0.217	0.222	0.225	-0.363	-0.367	0.219	0.210	0.243	0.239
22	-	-	0.211	0.214	-0.393	-0.386	0.212	0.218	0.211	0.215	0.226	0.229	-0.360	-0.351	0.197	0.201	0.222	0.224
23	-	-	-	-	-	-	-0.387	-0.381	-	-	-	-	-	-	-	-	-	-
24	-	-	-	-	-	-	-0.393	-0.391	-	-	-	-	-	-	-	-	-	-

Table S25. Cartesian coordinates of the optimized geometries for the axial and equatorial geometries of compounds 1-15 obtained at the M06-2X/aug-cc-pVTZ level in the gas phase.

1_{ax}				1_{eq}			
O	-0.14983000	0.87073000	0.91441000	O	-0.16513000	-0.97637000	-0.31342000
C	-0.55466000	-0.45149000	0.65297000	C	-0.55511000	0.27338000	0.22226000
C	0.63122000	-1.38268000	0.46904000	C	0.42259000	1.36137000	-0.16974000
C	1.57502000	-0.84915000	-0.60557000	C	1.83186000	0.98411000	0.28195000
C	1.94813000	0.59564000	-0.28360000	C	2.18863000	-0.41016000	-0.22715000
C	0.68663000	1.42127000	-0.09643000	C	1.09866000	-1.39457000	0.16703000
O	-1.34377000	-0.53327000	-0.50262000	O	-1.80734000	0.59203000	-0.26765000
C	-2.52869000	0.22915000	-0.42518000	C	-2.82116000	-0.30821000	0.13167000
H	-2.31276000	1.29788000	-0.37292000	H	-3.76483000	0.10409000	-0.21497000
H	-3.10559000	0.01675000	-1.32142000	H	-2.66715000	-1.29427000	-0.30593000
H	-3.11361000	-0.05194000	0.45645000	H	-2.84682000	-0.40023000	1.22252000
H	-1.15423000	-0.72933000	1.52622000	H	-0.59494000	0.16819000	1.32296000
H	0.26111000	-2.37625000	0.21928000	H	0.09919000	2.30491000	0.26894000
H	1.15343000	-1.44455000	1.42574000	H	0.37897000	1.45893000	-1.25623000
H	2.46473000	-1.47517000	-0.67110000	H	2.55341000	1.72282000	-0.06561000
H	1.07498000	-0.88894000	-1.57517000	H	1.87495000	0.98848000	1.37540000
H	2.55454000	1.03266000	-1.07813000	H	3.14670000	-0.74222000	0.17530000
H	2.53344000	0.63108000	0.63864000	H	2.27048000	-0.39737000	-1.31613000
H	0.91320000	2.43396000	0.23095000	H	1.27437000	-2.38210000	-0.25497000
H	0.13366000	1.47764000	-1.04023000	H	1.06259000	-1.49211000	1.26123000
8_{ax}				8_{eq}			
C	-0.54007000	-0.38577000	0.63355000	C	0.44035000	0.64485000	-0.29449000
C	0.01381000	0.93229000	0.09807000	C	0.04510000	-0.79884000	0.01497000
C	0.96290000	0.76635000	-1.06067000	C	-1.33600000	-1.16560000	-0.47723000
C	2.10359000	-0.17925000	-0.68473000	C	-2.38541000	-0.19936000	0.06221000
C	1.55985000	-1.51366000	-0.17743000	C	-2.02146000	1.23647000	-0.30499000
C	0.61900000	-1.30252000	1.00788000	C	-0.63411000	1.59444000	0.21907000
O	-1.31105000	-1.02259000	-0.35909000	O	1.64923000	1.02646000	0.29799000
C	-2.69117000	-0.71696000	-0.33711000	C	2.82652000	0.46955000	-0.25250000
H	-3.16270000	-1.34271000	-1.09070000	H	3.64989000	1.10481000	0.06552000
H	-3.12167000	-0.95196000	0.64091000	H	2.99583000	-0.54741000	0.10036000
H	-2.87925000	0.33188000	-0.56731000	H	2.78080000	0.46070000	-1.34569000
H	-1.15465000	-0.15507000	1.51090000	H	0.51354000	0.71215000	-1.39019000
F	-1.01675000	1.75640000	-0.25915000	F	0.95439000	-1.65962000	-0.54367000
F	0.65024000	1.56737000	1.13285000	F	0.12045000	-1.00399000	1.36141000
H	1.32454000	1.75449000	-1.34445000	H	-1.52730000	-2.19568000	-0.17646000
H	0.38598000	0.35652000	-1.89051000	H	-1.31449000	-1.13380000	-1.56861000
H	2.74863000	-0.33174000	-1.54937000	H	-3.36564000	-0.46452000	-0.33256000
H	2.71307000	0.28669000	0.09292000	H	-2.43769000	-0.29609000	1.14872000
H	2.38109000	-2.16708000	0.11667000	H	-2.75787000	1.93062000	0.09925000
H	1.01578000	-2.01649000	-0.97931000	H	-2.04403000	1.34864000	-1.39326000
H	0.20467000	-2.24918000	1.35295000	H	-0.35002000	2.60729000	-0.06295000
H	1.16399000	-0.85154000	1.83944000	H	-0.62029000	1.54496000	1.31014000
9_{ax}				9_{eq}			
O	-0.54694000	1.14455000	-0.87721000	O	0.49667000	1.14871000	0.17473000
C	0.15567000	-0.07925000	-0.88234000	C	-0.05947000	-0.02189000	-0.38039000
C	-0.75534000	-1.29421000	-0.70045000	C	0.67639000	-1.27159000	0.08228000
C	-1.77459000	-1.12094000	0.42618000	C	2.15868000	-1.14928000	-0.26328000
C	-2.47024000	0.22965000	0.29177000	C	2.71247000	0.15983000	0.29249000
C	-1.43253000	1.33437000	0.22111000	C	1.85743000	1.32307000	-0.18137000
C	1.33954000	-0.04988000	0.08889000	C	-1.53499000	-0.03426000	-0.00282000
C	2.15672000	1.21057000	0.05907000	C	-2.29414000	1.22093000	-0.32700000
H	1.54625000	2.05011000	0.38065000	H	-3.33835000	1.07685000	-0.05786000
H	3.00794000	1.09126000	0.72595000	H	-1.87420000	2.05040000	0.23435000
H	2.50707000	1.39665000	-0.95394000	H	-2.21813000	1.43041000	-1.39220000
H	0.62202000	-0.13215000	-1.86784000	H	-0.02050000	0.04040000	-1.47865000
H	-0.13951000	-2.18004000	-0.54557000	H	0.22993000	-2.14968000	-0.38312000
H	-1.29288000	-1.43171000	-1.64076000	H	0.55116000	-1.36214000	1.16238000
H	-2.50069000	-1.93284000	0.38494000	H	2.71230000	-2.00218000	0.12812000
H	-1.27923000	-1.17933000	1.39497000	H	2.28113000	-1.16035000	-1.35067000
H	-3.13965000	0.41228000	1.13324000	H	3.74535000	0.31645000	-0.02134000
H	-3.06963000	0.25386000	-0.62172000	H	2.69638000	0.13426000	1.38421000
H	-1.89282000	2.30578000	0.05136000	H	2.16872000	2.26282000	0.27002000
H	-0.86753000	1.38086000	1.15866000	H	1.93368000	1.42244000	-1.27293000
F	2.13746000	-1.11929000	-0.22270000	F	-1.66485000	-0.30514000	1.32842000

F	0.92463000	-0.27770000	1.37741000	F	-2.10616000	-1.09599000	-0.65505000
10_{ax}				10_{eq}			
C	0.25232000	0.02733000	-0.75451000	C	-0.15865000	-0.30369000	-0.44567000
C	-0.76571000	0.97664000	-0.12957000	C	0.62305000	0.93943000	-0.04384000
C	-1.53215000	0.43001000	1.04937000	C	2.09665000	0.86790000	-0.37408000
C	-2.22383000	-0.87968000	0.67281000	C	2.74253000	-0.39363000	0.18665000
C	-1.22728000	-1.88845000	0.10614000	C	1.99775000	-1.62960000	-0.30653000
C	-0.45598000	-1.30125000	-1.07723000	C	0.52908000	-1.56319000	0.09723000
C	1.54213000	-0.16322000	0.04843000	C	-1.63619000	-0.28793000	-0.04280000
C	2.42897000	1.03731000	0.25379000	C	-2.44515000	0.95036000	-0.31764000
H	3.36881000	0.68601000	0.67483000	H	-3.48514000	0.72181000	-0.09360000
H	2.61927000	1.52751000	-0.69828000	H	-2.10377000	1.76527000	0.31385000
H	1.96358000	1.74372000	0.93235000	H	-2.34894000	1.24103000	-1.36084000
H	0.56696000	0.49778000	-1.68878000	H	-0.14655000	-0.32438000	-1.53885000
F	-0.17988000	2.16528000	0.20845000	F	0.10386000	2.04499000	-0.66770000
F	-1.66006000	1.29491000	-1.12154000	F	0.46659000	1.16550000	1.29574000
H	-2.24928000	1.19343000	1.35099000	H	2.55774000	1.77638000	0.01317000
H	-0.82916000	0.27565000	1.86723000	H	2.18325000	0.88530000	-1.46239000
H	-2.72771000	-1.28855000	1.54775000	H	3.79157000	-0.42639000	-0.10535000
H	-2.99371000	-0.66970000	-0.07266000	H	2.71244000	-0.36130000	1.27804000
H	-1.75661000	-2.78143000	-0.22708000	H	2.44911000	-2.53341000	0.10223000
H	-0.53414000	-2.20092000	0.88674000	H	2.08099000	-1.69579000	-1.39558000
H	0.27586000	-2.01216000	-1.45368000	H	-0.01591000	-2.43667000	-0.25665000
H	-1.15698000	-1.10094000	-1.88927000	H	0.45403000	-1.55571000	1.18560000
F	2.27734000	-1.11388000	-0.61224000	F	-1.75314000	-0.59679000	1.28215000
F	1.26730000	-0.71327000	1.27584000	F	-2.21989000	-1.33971000	-0.71014000
11_{ax}				11_{eq}			
C	-1.26523000	0.53962000	0.57865000	C	-1.21754000	0.22801000	-0.27729000
C	-0.58093000	-0.69928000	1.15887000	C	-0.60783000	-1.08645000	0.20866000
C	0.45755000	-1.25358000	0.18654000	C	0.79784000	-1.27627000	-0.35681000
C	1.45240000	-0.17788000	-0.18083000	C	1.67064000	-0.08658000	-0.03340000
C	0.82182000	1.08346000	-0.71901000	C	1.08135000	1.23506000	-0.46587000
C	-0.23554000	1.61197000	0.24960000	C	-0.32683000	1.40303000	0.09641000
O	-1.95255000	0.25857000	-0.62926000	O	-2.48506000	0.48117000	0.28774000
C	-3.03702000	-0.62518000	-0.49190000	C	-3.49670000	-0.40437000	-0.12491000
H	-3.68393000	-0.32986000	0.34150000	H	-3.37483000	-1.40000000	0.30952000
H	-3.60840000	-0.58179000	-1.41598000	H	-4.44355000	0.00892000	0.21379000
H	-2.70897000	-1.65647000	-0.33185000	H	-3.51873000	-0.49624000	-1.21658000
H	-1.98399000	0.92975000	1.31017000	H	-1.31758000	0.18552000	-1.37415000
H	-1.31387000	-1.46627000	1.40714000	H	-1.22916000	-1.93196000	-0.08455000
H	-0.09588000	-0.41334000	2.09376000	H	-0.57350000	-1.06357000	1.30019000
H	1.00400000	-2.09349000	0.61414000	H	1.27557000	-2.16980000	0.04346000
H	-0.02218000	-1.58609000	-0.73476000	H	0.76587000	-1.37103000	-1.44401000
F	2.34876000	-0.66996000	-1.08653000	F	2.90098000	-0.25189000	-0.60422000
F	2.19348000	0.13544000	0.93480000	F	1.89279000	-0.05403000	1.31997000
H	1.61205000	1.81436000	-0.88740000	H	1.74529000	2.03145000	-0.13180000
H	0.36380000	0.84121000	-1.67738000	H	1.06688000	1.24436000	-1.55742000
H	-0.74115000	2.47207000	-0.18703000	H	-0.77656000	2.32520000	-0.26850000
H	0.24056000	1.93720000	1.17588000	H	-0.28865000	1.46653000	1.18559000
12_{ax}				12_{eq}			
C	-1.11286000	-0.28678000	0.67619000	C	-1.00417000	-0.64912000	-0.33420000
C	-0.52030000	0.96453000	0.03080000	C	-0.52255000	0.77059000	-0.02151000
C	0.61023000	0.67155000	-0.93058000	C	0.88459000	1.05705000	-0.50887000
C	1.65615000	-0.23081000	-0.30542000	C	1.86986000	-0.01747000	-0.09874000
C	1.08452000	-1.46062000	0.35911000	C	1.40162000	-1.41609000	-0.42243000
C	0.00076000	-1.06957000	1.36217000	C	0.01823000	-1.66050000	0.16831000
O	-1.69256000	-1.10569000	-0.31048000	O	-2.22209000	-0.96077000	0.27207000
C	-3.07399000	-0.89223000	-0.53477000	C	-3.36667000	-0.29344000	-0.22655000
H	-3.63837000	-1.03377000	0.39123000	H	-3.44355000	0.72020000	0.16561000
H	-3.39104000	-1.63589000	-1.26104000	H	-4.22781000	-0.87311000	0.09590000
H	-3.27080000	0.10548000	-0.92684000	H	-3.34858000	-0.25062000	-1.31940000
H	-1.86202000	0.04212000	1.40390000	H	-1.09139000	-0.70764000	-1.42987000
F	-1.49406000	1.65084000	-0.63457000	F	-1.35592000	1.68435000	-0.60954000
F	-0.09432000	1.79074000	1.02676000	F	-0.60970000	0.98938000	1.31404000
H	1.07034000	1.60512000	-1.25047000	H	1.20579000	2.02334000	-0.12254000
H	0.18908000	0.15625000	-1.79219000	H	0.86574000	1.10047000	-1.59748000
F	2.54590000	-0.59663000	-1.27266000	F	3.06187000	0.22848000	-0.71533000
F	2.37704000	0.48176000	0.61154000	F	2.11410000	0.07684000	1.23977000
H	1.90265000	-1.99247000	0.84237000	H	2.13426000	-2.11807000	-0.02709000
H	0.66552000	-2.09807000	-0.41881000	H	1.38742000	-1.51785000	-1.50921000
H	-0.43163000	-1.96060000	1.81310000	H	-0.33772000	-2.65663000	-0.08548000
H	0.42867000	-0.45959000	2.15793000	H	0.05403000	-1.59157000	1.25634000
13_{ax}				13_{eq}			
C	-1.12065000	0.13512000	0.28297000	C	-1.02604000	0.11228000	-0.34654000
C	-0.36563000	-1.18762000	0.43702000	C	-0.33436000	-1.20068000	0.03386000
C	0.76597000	-1.33985000	-0.55686000	C	1.06574000	-1.33450000	-0.53016000
C	1.69077000	-0.13711000	-0.55478000	C	1.92231000	-0.12420000	-0.21463000
C	0.97829000	1.19808000	-0.66173000	C	1.26813000	1.19665000	-0.56719000

C	-0.14717000	1.31284000	0.34311000	C	-0.13776000	1.31119000	-0.00970000
O	-1.72670000	0.15920000	-0.97680000	O	-2.24614000	0.25813000	0.29425000
C	-3.12982000	-0.04705000	-0.96049000	C	-3.37455000	-0.13255000	-0.46920000
H	-3.62311000	0.74256000	-0.38933000	H	-3.34552000	-1.19640000	-0.70693000
H	-3.46288000	-0.00588000	-1.99333000	H	-4.24704000	0.07857000	0.14177000
H	-3.37678000	-1.02106000	-0.53585000	H	-3.43223000	0.45077000	-1.39219000
H	-1.83798000	0.23444000	1.10266000	H	-1.13739000	0.10723000	-1.43866000
F	-1.25317000	-2.20417000	0.25457000	F	-1.09329000	-2.23137000	-0.44214000
F	0.09113000	-1.30212000	1.70674000	F	-0.31056000	-1.33389000	1.37641000
H	1.33698000	-2.23700000	-0.32461000	H	1.53306000	-2.22889000	-0.12132000
H	0.32658000	-1.43062000	-1.54869000	H	0.99631000	-1.43261000	-1.61315000
F	2.54943000	-0.25348000	-1.60674000	F	3.08977000	-0.22782000	-0.91090000
F	2.46191000	-0.15573000	0.56391000	F	2.26170000	-0.13288000	1.09826000
H	1.69241000	2.00428000	-0.50393000	H	1.87167000	2.01641000	-0.18102000
H	0.54561000	1.27913000	-1.65759000	H	1.21696000	1.27427000	-1.65281000
F	-0.84249000	2.45489000	0.11196000	F	-0.71869000	2.42398000	-0.53466000
F	0.34676000	1.41107000	1.60174000	F	-0.09421000	1.49047000	1.32766000
14_{ax}				14_{eq}			
C	1.10890600	0.44603600	0.73836900	C	-1.02437000	-0.16888000	0.12649000
C	-0.03501000	-0.51245600	1.08866500	C	0.14142000	-0.98977000	-0.40647000
C	-1.13038000	-0.51051700	0.04646100	C	1.46490000	-0.38316000	-0.00843000
C	-1.62988400	0.86652000	-0.32039100	C	1.61288000	1.07131000	-0.38493000
C	-0.46749300	1.78495000	-0.69039700	C	0.43755000	1.87883000	0.16022000
C	0.56473000	1.83576700	0.43433300	C	-0.89670000	1.29183000	-0.29905000
O	1.84662000	0.05223700	-0.39921700	O	-2.19496000	-0.78463000	-0.36629000
C	2.46886000	-1.20513200	-0.29952100	C	-3.38406000	-0.33715000	0.23802000
H	2.98547100	-1.31520400	0.66090100	H	-4.18225000	-0.99319000	-0.10020000
H	3.20050400	-1.26479300	-1.10157400	H	-3.62973000	0.68905000	-0.04751000
H	1.75241900	-2.02148700	-0.41716000	H	-3.31503000	-0.39306000	1.32967000
H	1.78721800	0.48860300	1.60051000	H	-1.02525000	-0.21929000	1.22448000
H	0.31253300	-1.53581300	1.22727400	H	0.09313000	-2.01281000	-0.03615000
H	-0.47947700	-0.18708000	2.03042200	H	0.08747000	-1.01435000	-1.49612000
F	-2.17721600	-1.26825000	0.50213900	F	2.48322000	-1.10909000	-0.55636000
F	-0.68698700	-1.14913800	-1.08069400	F	1.61688000	-0.50421000	1.35198000
H	-2.34451600	0.75949400	-1.13605600	H	2.56742000	1.42759000	0.00123000
H	-2.16388000	1.25787800	0.54817800	H	1.64333000	1.12690000	-1.47455000
H	-0.84511700	2.78450100	-0.90215200	H	0.52351000	2.91601000	-0.16045000
H	0.01198200	1.41683900	-1.59762600	H	0.48014000	1.87874000	1.25153000
H	1.39839800	2.48168600	0.16112200	H	-1.71858000	1.87778000	0.11224000
H	0.11096200	2.24296900	1.34177700	H	-0.97523000	1.33524000	-1.38955000
15_{ax}				15_{eq}			
C	-0.84426000	-0.86866000	-0.92551000	C	-1.18622000	-0.40896000	0.07553000
C	-0.97690000	0.65344000	-1.05036000	C	-0.83954000	0.98058000	-0.46354000
C	-0.15681000	1.39204000	-0.01795000	C	0.56874000	1.37254000	-0.08018000
C	1.29779000	0.97265000	0.01981000	C	1.62358000	0.35593000	-0.46587000
C	1.46395000	-0.53405000	0.06486000	C	1.23134000	-1.04341000	-0.03612000
C	0.62215000	-1.28461000	-0.94772000	C	-0.17634000	-1.43469000	-0.41812000
O	-1.40239000	-1.39455000	0.25251000	O	-2.44893000	-0.84246000	-0.36986000
C	-2.78089000	-1.16006000	0.41334000	C	-3.53581000	-0.18859000	0.24472000
H	-3.32886000	-1.37677000	-0.51089000	H	-4.43815000	-0.70510000	-0.07136000
H	-3.12730000	-1.83031000	1.19550000	H	-3.45673000	-0.23858000	1.33509000
H	-2.98236000	-0.13084000	0.71906000	H	-3.60737000	0.85940000	-0.05768000
H	-1.34881000	-1.31631000	-1.79149000	H	-1.16980000	-0.38236000	1.17204000
H	-2.01303000	0.97770000	-0.96565000	H	-1.51043000	1.74592000	-0.07581000
H	-0.61721000	0.95578000	-2.03502000	H	-0.91495000	0.96887000	-1.55245000
F	-0.20878000	2.73301000	-0.28329000	F	0.88423000	2.56733000	-0.65750000
F	-0.71904000	1.23938000	1.21252000	F	0.61663000	1.58513000	1.26800000
H	1.78872000	1.42012000	0.88245000	H	2.57720000	0.62622000	-0.01560000
H	1.77346000	1.33793000	-0.88989000	H	1.72348000	0.36376000	-1.55064000
F	2.78253000	-0.81979000	-0.16967000	F	2.11512000	-1.92828000	-0.57896000
F	1.20870000	-0.98290000	1.31968000	F	1.37325000	-1.15542000	1.31752000
H	0.71041000	-2.35023000	-0.74276000	H	-0.39220000	-2.41594000	0.00097000
H	1.04333000	-1.08247000	-1.93333000	H	-0.23713000	-1.50165000	-1.50568000

3 References

[1] Gaussian 16, Revision C.01, M. J. Frisch, G. W. Trucks, H. B. Schlegel, G. E. Scuseria, M. A. Robb, J. R. Cheeseman, G. Scalmani, V. Barone, G. A. Petersson, H. Nakatsuji, X. Li, M. Caricato, A. V. Marenich, J. Bloino, B. G. Janesko, R. Gomperts, B. Mennucci, H. P. Hratchian, J. V. Ortiz, A. F. Izmaylov, J. L. Sonnenberg, D. Williams-Young, F. Ding, F. Lipparini, F. Egidi, J. Goings, B. Peng, A. Petrone, T. Henderson, D. Ranasinghe, V. G. Zakrzewski, J. Gao, N. Rega, G. Zheng, W. Liang, M. Hada, M. Ehara, K. Toyota, R. Fukuda, J. Hasegawa, M. Ishida, T. Nakajima, Y. Honda, O. Kitao, H. Nakai, T. Vreven, K. Throssell, J. A. Montgomery, Jr., J. E. Peralta, F. Ogliaro, M. J. Bearpark, J. J. Heyd, E. N. Brothers, K. N. Kudin, V. N. Staroverov, T. A. Keith, R. Kobayashi, J. Normand, K. Raghavachari, A. P. Rendell, J. C. Burant, S. S. Iyengar, J. Tomasi, M. Cossi, J. M. Millam, M. Klene, C. Adamo, R. Cammi, J. W. Ochterski, R. L. Martin, K. Morokuma, O. Farkas, J. B. Foresman, and D. J. Fox, Gaussian, Inc., Wallingford CT, **2016**.

[2] F. Neese, *Wiley Interdiscip. Rev. Comput. Mol. Sci.* **2012**, *2*, 73–78.

[3] E. D. Glendening, J. K. Badenhoop, A. E. Reed, J. E. Carpenter, J. A. Bohmann, C. M. Morales, C. R. Landis, F. Weinhold, (Theoretical Chem. Institute, Univ. Wisconsin, Madison, WI, **2018**); http//nbo7.chem.wisc.edu/**.**

[4] AIMALL (Version 19.10.12), T. A. Keith, *TK Gristmill Software, Overl. Park KS, USA,* **2019**.

[5] E. R. Johnson, S. Keinan, P. Mori-Sánchez, J. Contreras-García, A. J. Cohen, W. Yang, *J. Am. Chem. Soc.* **2010**, *132*, 6498–6506.
